# Understanding the Metabolism of Proteolysis Targeting Chimeras (PROTACs): The Next Step toward Pharmaceutical Applications

**DOI:** 10.1021/acs.jmedchem.0c00793

**Published:** 2020-10-07

**Authors:** Laura Goracci, Jenny Desantis, Aurora Valeri, Beatrice Castellani, Michela Eleuteri, Gabriele Cruciani

**Affiliations:** † Department of Chemistry, Biology and Biotechnology, University of Perugia, 06123 Perugia, Italy; ‡ Molecular Horizon, srl, Bettona 06084, Italy; § Montelino Therapeutics, LLC, 7 Powdermill Lane, Southborough, Massachusetts 01772 Unites States

## Abstract

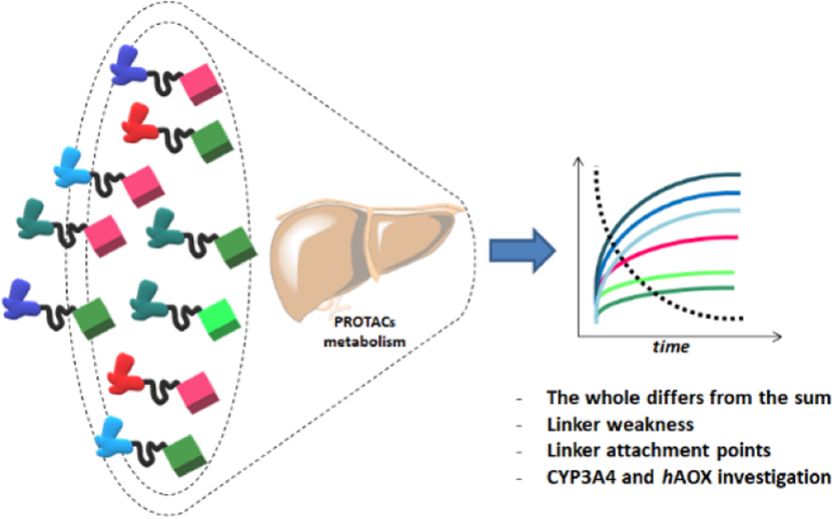

Hetero-bifunctional PROteolysis TArgeting Chimeras (PROTACs) represent a new emerging class of small molecules designed to induce polyubiquitylation and proteasomal-dependent degradation of a target protein. Despite the increasing number of publications about the synthesis, biological evaluation, and mechanism of action of PROTACs, the characterization of the pharmacokinetic properties of this class of compounds is still minimal. Here, we report a study on the metabolism of a series of 40 PROTACs in cryopreserved human hepatocytes at multiple time points. Our results indicated that the metabolism of PROTACs could not be predicted from that of their constituent ligands. Their linkers’ chemical nature and length resulted in playing a major role in the PROTACs’ liability. A subset of compounds was also tested for metabolism by human cytochrome P450 3A4 (CYP3A4) and human aldehyde oxidase (*h*AOX) for more in-depth data interpretation, and both enzymes resulted in active PROTAC metabolism.

## Introduction

Rational drug design represents an essential approach to optimize time and cost in drug discovery and development,^1^ but it remains a challenging task. Indeed, not only is drug potency a critical feature, but also absorption, distribution, metabolism, and excretion (ADME) properties require optimization by modulating the chemical structure of the candidate. Drugs undergo biotransformations, and thus the optimization of the drug structure *per se* could be useless when significant metabolic liability, generating novel compounds (metabolites), occurs. In the last two decades, many efforts have been made to decode and predict the metabolic fate of drugs,^2^ and *in silico* models,^3−6^*in vitro* assays,^2^ and hybrid approaches (i.e., innovative assays associated with software-assisted data processing)^7,8^ have been developed to identify the “soft spots” of drugs. Despite signs of progress in the field, all available ADME tools have been calibrated mainly using the chemical space of small molecules, witnessing the outstanding impact that the Lipinski rules^9^ have had in pharmaceutical research in the past. In the comfortable space of small molecules, the accuracy and sensitivity of the models are usually very good.^10−14^ Nowadays, the chemical space of the drugs is quickly expanding, ranging from peptides or peptidomimetics^15^ to Proteolysis Targeting Chimeras (PROTACs)^16−26^ and their analogues.^27−29^ PROTACs can be defined as hetero-bifunctional molecules that induce a ligand to bind with the protein of interest (POI), another ligand to recruit an E3 ubiquitin ligase, and a linker to concatenate the two ligands.^17^ The formation of the ternary complex composed of the POI, the PROTAC, and the E3 ligase allows the E2 ubiquitin-conjugating enzyme to transfer ubiquitin to the surface of the POI, inducing its proteasomal-dependent degradation.^30^ One of the main advantages of PROTACs is that they can degrade proteins regardless of their function, thus turning into druggable also the “undruggable”, due to their innovative mechanism of action.^16^ Degradation by PROTACs is a catalytic process, due to the dissociation of the complex after polyubiquitination of the POI, indicating that PROTACs can be recycled for successive rounds of degradation and thus used at reduced doses.^31^ Therefore, PROTACs represent an innovative class of compounds that overcome traditional limitations, opening a new therapeutic modality and, at the same time, breaking the rules used so far with the potential to revolutionize drug discovery. As extensively reviewed,^32−36^ hundreds of PROTAC molecules have been developed so far, targeting a wide range of different disease-related protein targets. The entry in phase I clinical trial in 2019 of the first two oral PROTACs (ARV-110 and ARV-471) for the treatment of metastatic castration-resistant cancer and metastatic breast cancer (NCT0888612 and NCT04072952) has focused attention even more on this innovative therapeutic paradigm.^37,38^ Despite their intriguing capabilities, PROTACs are characterized by a high molecular weight (600–1400 Da),^39^ making the delivery and bioavailability of PROTACs the most significant hurdles to overcome on the way to the clinic.^40^ Thus, better understanding and prediction of the ADME properties of PROTACs represent an urgent need for their rational design. To date, the evaluation of the ADME properties of this class of compounds is still minimal, with only a few studies on their experimental physical–chemical properties available^41−43^ and only one paper about PROTAC metabolism has been published.^35^ Nevertheless, preliminary studies on small subsets of PROTACs, whose log *P* was experimentally measured, indicate that traditional *in silico* tools for property prediction may fail,^41^ likely due to their peculiar structural features compared with traditional druglike compounds used for generation of predictive models. Therefore, there is an urgent need to collect experimental physicochemical and ADME data on PROTACs to shed light on their peculiar behavior and to be used for modeling purposes. Here, focusing on the human metabolism, a collection of 40 PROTACs (compounds **1**–**40**, Supporting Information Table S1) was studied, assessing their metabolism in cryopreserved human hepatocytes at multiple time points. Their enzymatic biotransformations were also compared with those of the constituent ligands (compounds **41**–**46**, Supporting Information Table S2). Both metabolic rate (half-life value) and soft spot identification were investigated. In addition, a subset of compounds was also tested for metabolism by human CYP3A4 and *h*AOX for deeper data interpretation, representing the principal isoenzyme involved in liver metabolism (including large substrates)^44,45^ and one of the emerging enzymes in metabolism studies,^46−49^ respectively. The complete data set with more experimental details is provided in the Supporting Information.

## Results and Discussion

### Chemistry

**Scheme 1:**
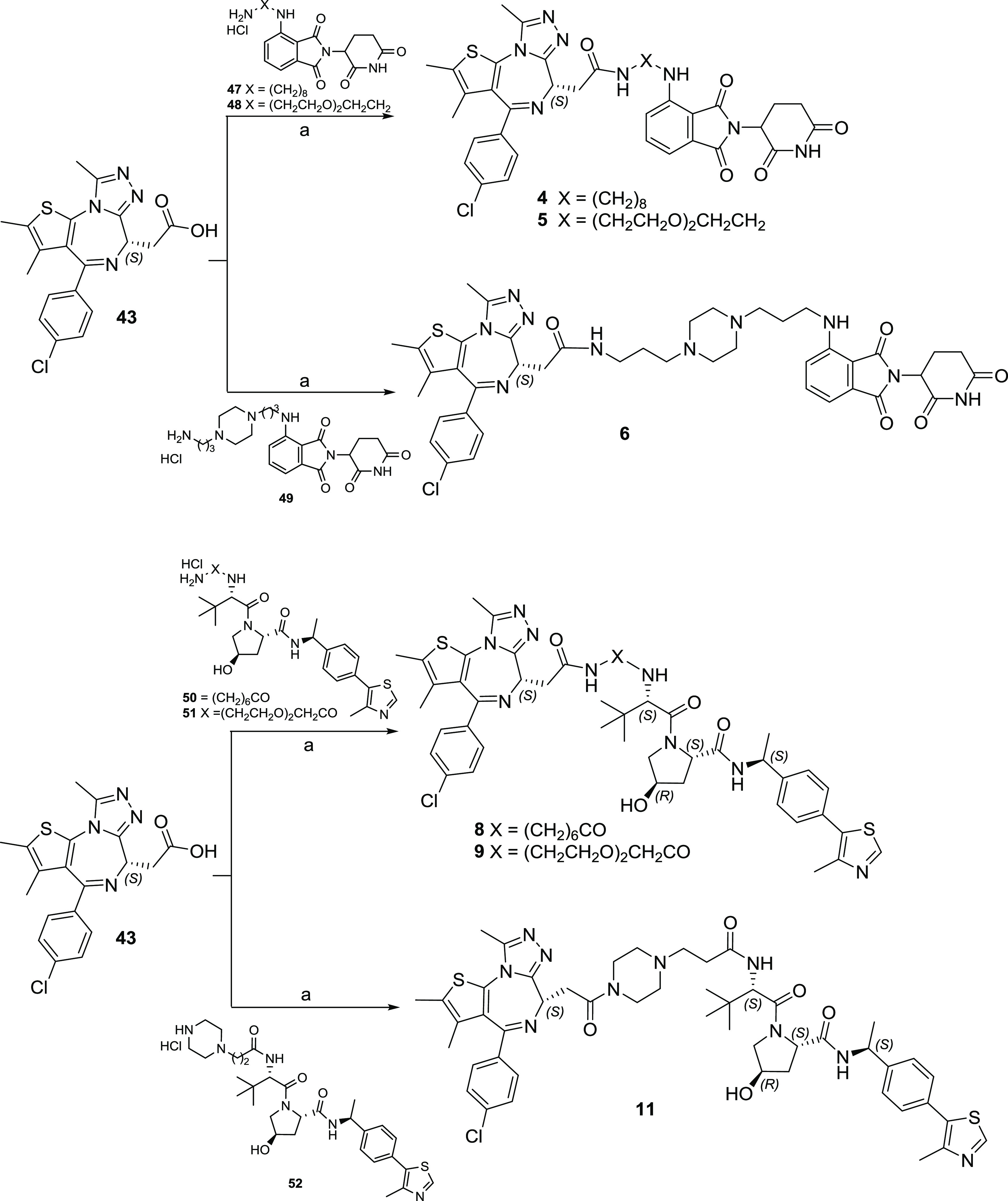
Syntheses of JQ1-Based PROTACs **4**–**6, 8, 9**, and **11** Reagents and conditions: (a) HATU, DIPEA, dry DMF, room temperature (rt).

The synthesis of JQ1-based PROTACs **4**–**6, 8**, **9**, and **11** was accomplished according to Scheme 1. Briefly, derivative **43** was coupled by amidation reaction with the appropriated E3 ligase ligand properly functionalized with linkers of different lengths in the presence of 1-[bis(dimethylamino)methylene]-1*H*-1,2,3-triazolo[4,5-*b*]pyridinium 3-oxid hexafluorophosphate (HATU) and *N,N*-diisopropylethylamine (DIPEA) at room temperature in dimethylformamide (DMF).

**Scheme 2:**
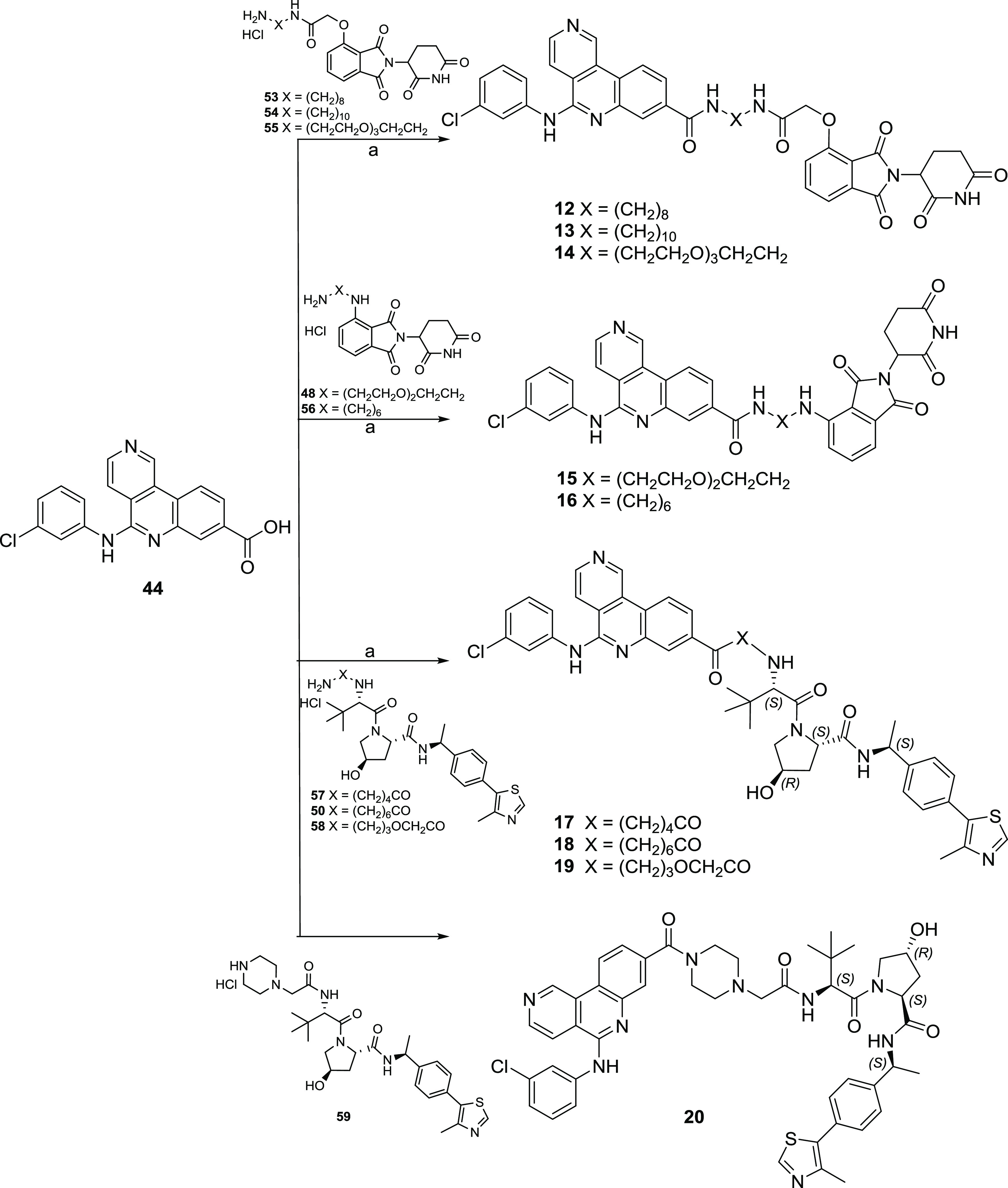
Syntheses of CX4945-Based PROTACs **12**–**20** Reagents and conditions: (a) HATU, DIPEA, dry DMF, rt.

Analogously, as shown in Scheme 2, CX4945-based PROTACs **12**–**20** were obtained by HATU-mediated amidation reaction between derivative **44** and the appropriated E3 ligase-linker intermediate.

**Scheme 3:**
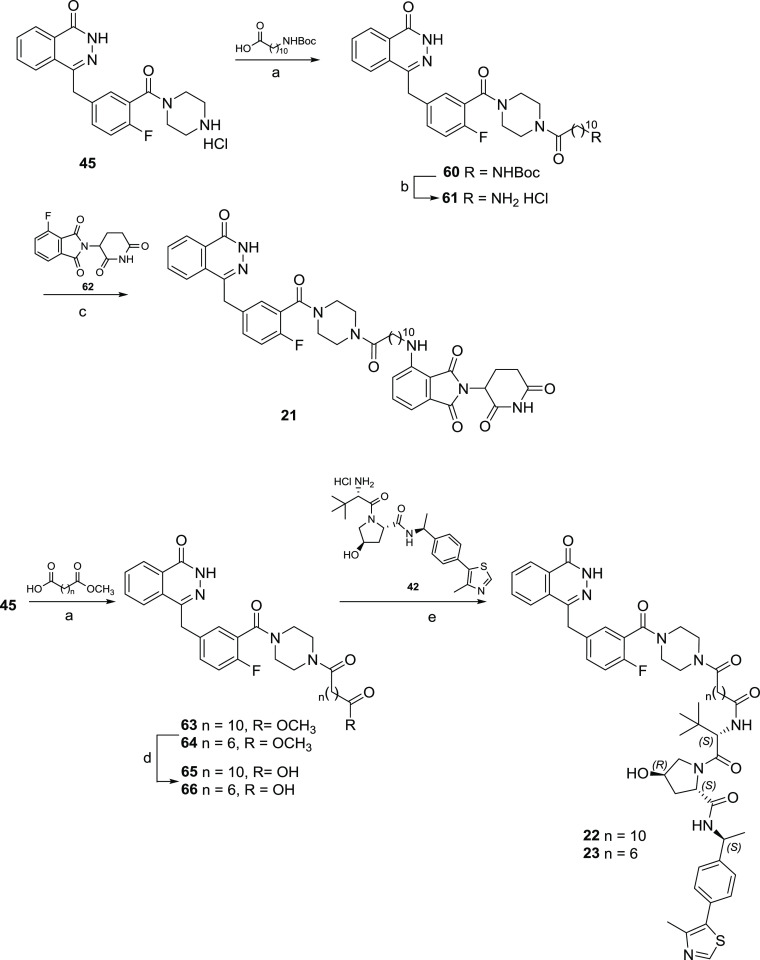
Syntheses of Olaparib-Based PROTACs **21**–**23** Reagents and conditions: (a) HBTU, Et_3_N, dry DMF, rt; (b) 4.0N HCl in dioxane, rt; (c) DIPEA, dry DMF, 70 °C; (d) LiOH monohydrate, tetrahydrofuran (THF):H_2_O (2:1), rt; (e) HATU, DIPEA, dry DMF, rt.

**Scheme 4:**
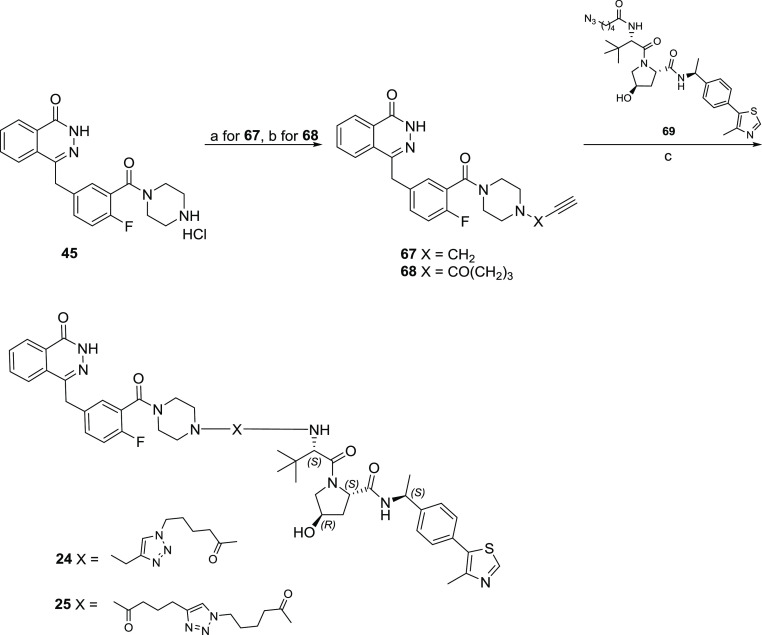
Synthesis of Olaparib-Based PROTACs **24** and **25** Reagents and conditions: (a) 3-bromoprop-1-yne, K_2_CO_3_, KI, ACN, reflux; (b) HBTU, Et_3_N, dry DMF, rt; (c) CuSO_4_, sodium ascorbate, DMF/*t*BuOH/H_2_O (1:1:1), rt.

The olaparib-based PROTACs **21**–**25** were synthesized as depicted in Schemes 3 and 4. For cereblon (CRBN)-addressing PROTAC **21**, derivative **45**(50) was first reacted with 11-((*tert*-butoxycarbonyl)amino)undecanoic acid by *N*,*N*,*N*′,*N*′-tetramethyl-*O*-(1*H*-benzotriazol-1-yl)uronium hexafluorophosphate (HBTU)-mediated amidation reaction leading to Boc-protected intermediate **60**, which after Boc-deprotection reaction gave the key intermediate **61**. Then, compound **62** was reacted with fluorothalidomide **62**(51) in the presence of DIPEA at 70 °C in DMF (Scheme 3). For the von Hippel-Lindau (VHL)-addressing PROTACs **22** and **23**, derivative **45**(50) was first reacted with the appropriate dicarboxylic acid monomethyl ester linker by amidation reaction in the presence of HBTU and Et_3_N at room temperature in dry DMF to furnish intermediates **63**–**64**. The successive basic hydrolysis of methyl esters **63**–**64** gave intermediates **65**–**66**, which in turn were finally coupled with derivative **42**(52) by HATU-mediated amidation reaction to afford PROTACs **22** and **23**, respectively (Scheme 3).

For the VHL-addressing PROTACs **24** and **25** (Scheme 4), an alkynyl group was introduced by reacting derivative **45**(50) with propargyl bromide, generating intermediate **67**, or by coupling it with hex-5-ynoic acid, generating intermediate **68**. Thus, final PROTACs **24** and **25** were obtained through the copper-assisted click reaction coupling the alkynyl derivatives **67** and **68** with azide-containing VHL derivative **69**.

**Scheme 5:**
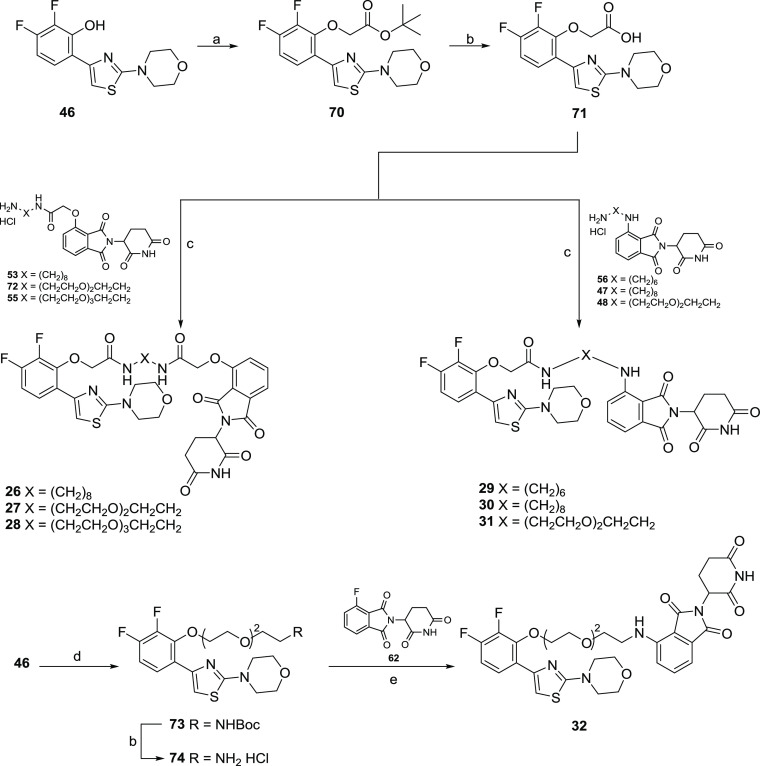
Syntheses of AR Ligand-Based PROTACs **21**–**27** Reagents and conditions: (a) *tert*-butyl bromoacetate, K_2_CO_3_, ACN, rt; (b) 4.0N HCl in dioxane, rt; (c) HATU, DIPEA, dry DMF, rt; (d) PPh_3_, DIAD, dry THF, 0 °C-rt; (e) DIPEA, dry DMF, 70 °C.

The syntheses of AR ligand-based CRBN-addressing PROTACs **26**–**32** are shown in Scheme 5. A substitution reaction between derivative **46**(53) and *tert*-butyl 2-bromoacetate in the presence of K_2_CO_3_ in acetonitrile at room temperature gave intermediate **70**, which after acidic hydrolysis afforded the key intermediate **71**. The successive amidation reaction in the presence of HATU and DIPEA at room temperature in DMF between compound **71** and the appropriated thalidomide-linker intermediate furnished PROTACs **26**–**31**. For the synthesis of PROTAC **32**, a first Mitsunobu reaction between derivative **46**(53) and the hydroxyl poly(ethylene glycol) (PEG)-linker gave the Boc-protected intermediate **73**. The Boc-deprotection reaction of **73** furnished intermediate **74**, which was then reacted with fluothalidomide **62**(51) in the presence of DIPEA at 70 °C in DMF to afford PROTAC **32**.

### Data Set Selection

**Figure 1:**
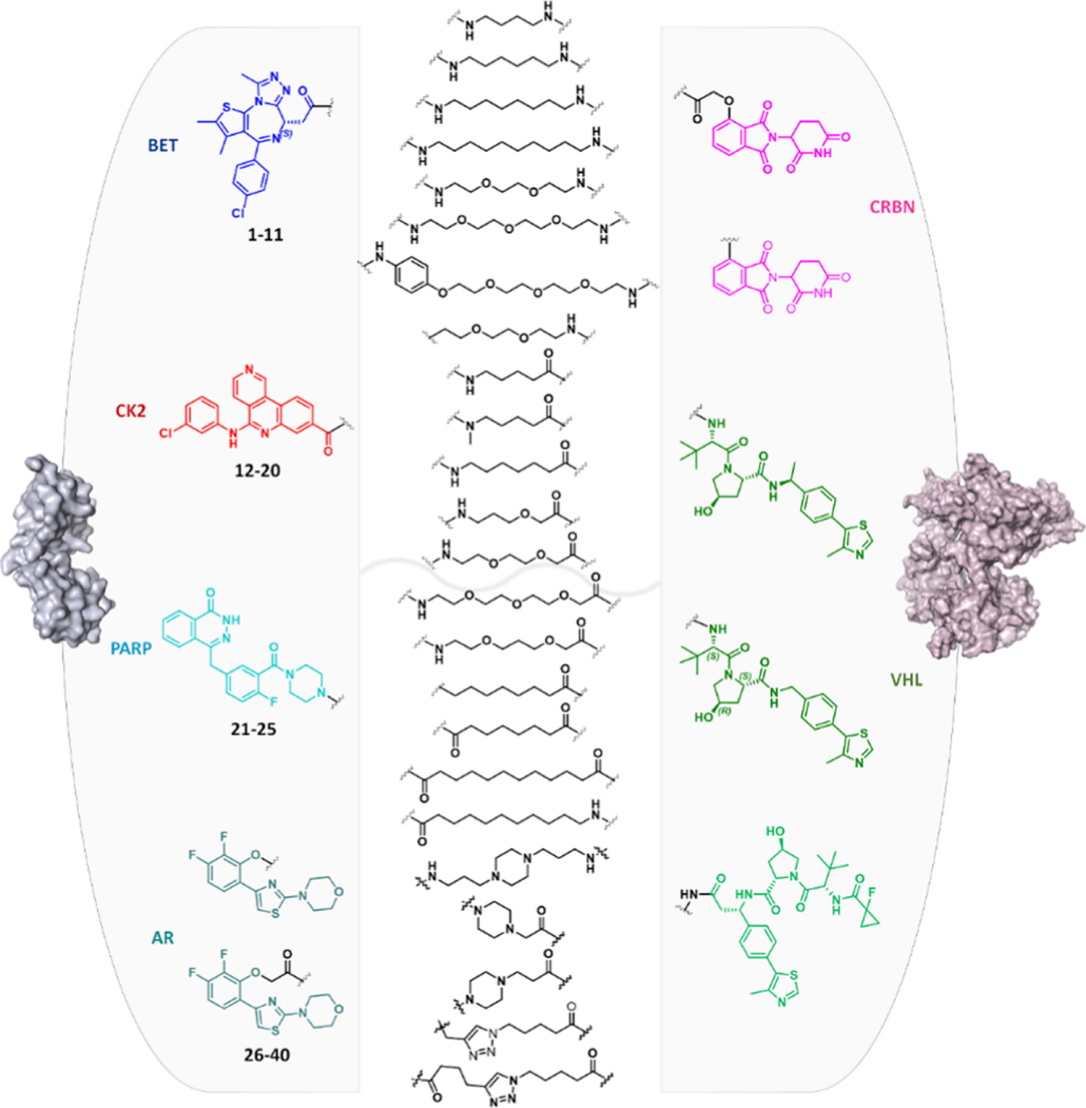
Scheme of building blocks characterizing the data set of tested PROTACs.

With the aim of covering a large chemical diversity, various combinations of ligands for four target proteins, ligands for two E3 ligases, and nineteen linkers were selected to give a final data set of 40 PROTACs (Figure 1). In particular, concerning the selection of ligands for target proteins, the bromodomain and extra-terminal (BET) inhibitor (+)-JQ1,^54^ the casein kinase 2 (CK2) inhibitor CX4945,^55^ the FDA-approved poly(ADP-ribose) polymerase (PARP) inhibitor olaparib,^50^ and an androgen receptor DNA-binding domain binder^53^ were used. Concerning the ligands for E3 ligases, binders for cereblon (CRBN) and von Hippel-Lindau (VHL) were selected, with these being two of the four most commonly used E3 ligases in PROTAC synthesis, together with cell inhibitor of apoptosis protein (cIAP) and mouse double minute 2 homolog (MDM2).^56^ Finally, aliphatic, polyethylene glycol (PEG)-based, and cyclic linkers were variably combined, modulating their length and anchor point (Figure 1). The chemical structures of the entire data set are provided in the Supporting Information (Table S1). Among them, five compounds were commercially available (Supporting Information Table S1), entries **1 (dBet1)**,^57^**2 (dBet6)**,^58^**3 (ARV-825)**,^59^**7 (MZ1)**,^60^ and **10 (ARV-771)**,^52^ 15 were kindly provided by Montelino Therapeutics Inc. (Supporting Information Table S1, entries **26**–**40**), while the others were designed and synthesized in house to increase chemical and structural variability.

### Optimization of the Metabolic Stability Assay for PROTACs

**Figure 2:**
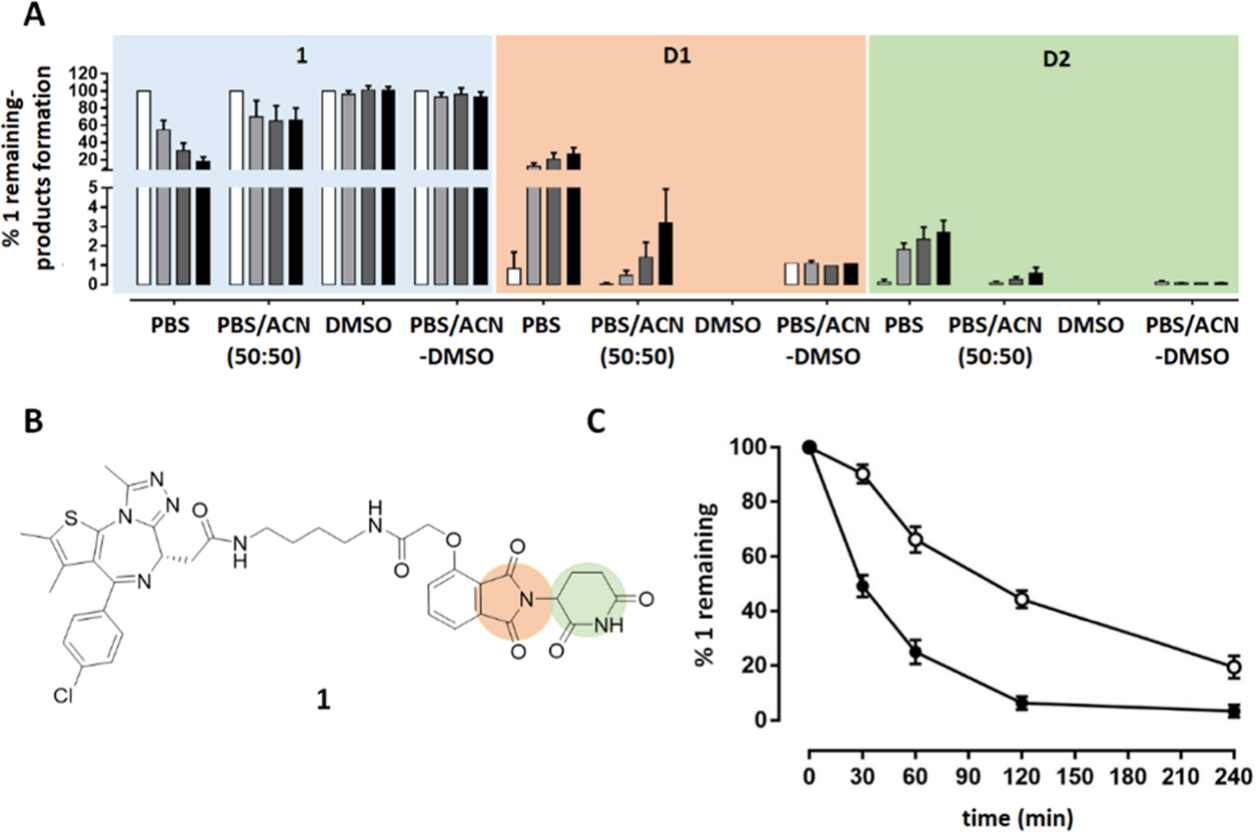
Nonenzymatic stability of **1** (**dBet1**) in the autosampler during LC–MS analysis acquisitions. (A) Percentage of the remaining **1** and percentage of formation of the degradation products resulted by the hydrolysis of the phthalimide moiety (D1, orange) or of the glutarimide moiety (D2, green) at the four time points (0, 3, 6, 12 h) in the different solutions. (B) Chemical structure of **1** (**dBet1**), with highlighted phthalimide (orange) and glutarimide (green) rings. (C) Comparison of the metabolic profiles of **1** (**dBet1**) in cryopreserved human hepatocytes for 4 h using the PBS/ACN (filled circles) or the PBS/ACN-DMSO (empty circles) protocol.

Metabolic stability of PROTACs has been rarely discussed in the literature, with only one paper published to date.^35^ In that paper, Zhou et al.^35^ evaluated the metabolism in mouse liver microsomes (phase I metabolism only) of one PROTAC, known to degrade the BET proteins with thalidomide as the CRBN ligand, after 20 and 40 min incubation times. The major metabolite detected was proposed to be a hydroxylated product, with the site of transformation occurring in the alkyl linker. In the present study, the aim was to test a diverse data set of PROTACs for their metabolic stability in cryopreserved human hepatocytes at multiple time points within a time range of 4 h. Differently from liver microsomes, cryopreserved human hepatocytes contain all phase I and II metabolic enzymes, with all necessary cofactors, and are compatible with longer incubation times.^61^ In commonly used protocols for metabolic stability assays, enzymatic reactions are quenched at the desired time of incubation by adding an organic solvent (e.g., acetonitrile) to the enzyme-containing solution to induce protein precipitation^62−64^ and, after centrifugation, the water-containing supernatant is collected and analyzed by liquid chromatography–mass spectrometry (LC–MS). However, such a protocol would not prevent the nonenzymatic degradation of PROTACs in the autosampler during LC–MS analysis.^65^ Therefore, it is especially critical for high-throughput screenings, in which a large number of samples are collected in the autosampler simultaneously and analyzed in a long sequence of analysis.^65^ In our studies on PROTAC metabolism, we reasoned that this could be a critical point to be addressed also taking into account that the rapid degradation of thalidomide^66^ and thalidomide-containing PROTACs in aqueous solution has already been reported elsewhere.^43^ Therefore, studies on the potential nonenzymatic degradation of substrates in the autosampler during LC–MS analysis were conducted on the commercial PROTAC **1** (**dBet1**), a potent Bromodomain-containing protein 4 (BRD4) protein degrader that is composed of (+)-JQ1 linked to thalidomide through an aliphatic linker. Compound **1** (**dBet1**) was incubated in three different solvents: (1) in pure phosphate buffer at pH = 7.4 (PBS); (2) in a mixture of PBS/acetonitrile (1:1 v/v), which is the most common composition of the supernatant injected in the LC–MS instrument in metabolism assays (named here PBS/ACN); and (3) in pure dimethyl sulfoxide (DMSO). A fourth condition entailed the incubation of **1** (**dBet1**) in PBS/acetonitrile (1:1, v/v) and immediately after the sample was dried under a nitrogen stream (to remove the solvent) and redissolved in DMSO before injection in the LC–MS system. The latter protocol is here named as PBS/ACN-DMSO and was designed to evaluate whether the removal of the first solvent and the resuspension in DMSO could prevent further degradation in the autosampler. Thus, the nonenzymatic stability of **1** (**dBet1**) in the four solvents and solvent mixtures was analyzed by LC–MS for 12 h at 37 °C, with injections at 0, 3, 6, and 12 h. Figure 2 illustrates the results of the stability of **1** (**dBet1**). As expected, compound **1** (**dBet1**) rapidly degrades when stored in the autosampler in pure PBS (Figure 2A). Degradation was also observed in the presence of the PBS/ACN solution, although to a lower extent, and it occurred during the first three hours, becoming constant with time. In DMSO, the solution of **1** (**dBet1**) became very stable over time. When the PBS/ACN solution was removed by a nitrogen stream and then replaced with an equal volume of DMSO (protocol PBS/ACN-DMSO), the substrate degradation within the first three hours was reduced to about 10%, remaining rather constant with time. The formation of two degradation products was also monitored (Figure 2A), corresponding to the hydrolysis of the phthalimide (degradation product D1) or glutarimide groups (degradation product D2) in the thalidomide moiety (Figure 2A,B). When the PBS/ACN-DMSO protocol was used, the formation of the degradation product was observed in a limited amount, and their concentration was constant over time, indicating that they formed during the PBS/ACN solution removal and not during storage in the autosampler.

Finally, protocols PBS/ACN and PBS/ACN-DMSO were compared in a real metabolism assay of **1** (**dBet1**). The study of the metabolic stability of this PROTAC in cryopreserved human hepatocytes was conducted at five time points (0, 30, 60, 120, and 240 min). Monitoring metabolic stability by a kinetic approach allows not only the half-life calculation for the substrate but also reduction in false positives in the characterization of metabolites. Samples for each time point were analyzed by LC–MS/MS, and raw data were analyzed using Mass-MetaSite software^7,67^ in the WebMetabase platform.^68−70^Figure 2C shows that the PBS/ACN-DMSO protocol significantly increases the stability of **1** (**dBet1**) during analysis. Therefore, the final method for the metabolic stability assay used in this study included the PBS/ACN-DMSO protocol (see the Methods section for the whole procedure), to reduce the risk of further degradation of the substrate in the autosampler during analysis.

### Metabolic Stability of Constituent Ligands for Tested PROTACs

Before performing an extensive study of the metabolic stability of the 34 PROTACs, the ligands used in PROTACs’ design and synthesis were tested for their metabolism in cryopreserved human hepatocytes, to have a reference on the behavior of the units connected in the final PROTAC structures. The same experimental protocol was used for both PROTACs and ligands (see the methods section). Metabolic stability was first expressed in terms of the half-life (t1/2) of the parent compound, as it represents a commonly used parameter to judge the intrinsic stability of a compound (Supporting Information Table S2).^61^ Concerning the structure of the tested ligands, an olaparib analogue lacking the carboxycyclopropyl moiety (compound **45**, Supporting Information Table S2) was used a reference for the PROTACs (**21**–**25**, Supporting Information Table S1) targeting PARP. This carboxycyclopropyl moiety, which represents a solution to improve oral absorption,^71^ was assumed to only slightly affect the binding with the target^72^ and was removed to allow the use of the unbound nitrogen of the piperazine ring as the anchor point for the linker. As free compounds, ligands used in this study for PROTAC design (**41**–**46**, Supporting Information Table S2) were characterized by good metabolic stability (t1/2 higher than 90 min), with the exception of the AR ligand (compound **46**) showing a t1/2 of less than 20 min (Supporting Information Table S2).

### Metabolic Stability of PROTACs in Cryopreserved Human Hepatocytes

As for the free ligands, metabolic stability of the whole set of PROTACs in cryopreserved human hepatocytes was studied over a four-hour incubation period (see the Method section). An example of the complete kinetic behavior of the disappearance of the substrate and the appearance of metabolites with time is shown for compound **7** (**MZ1**) in Figure S1 (Supporting Information). Based on observed kinetic data for the entire data set, the half-life for each PROTAC was calculated, and results are shown in the Supporting Information Table S1.

The analysis of the half-lives led to a number of observations. First, for PROTACs bearing ligands targeting BET, CK2, and PARP (compounds **1**–**25** in the Supporting Information Table S1), the use of the thalidomide moiety as the E3 ligase binder led to lower t1/2 values compared to PROTACs bearing the VHL ligand (i.e., **5** versus **10**, **16** versus **18**, **21** versus **22** in the Supporting Information Table S1). PROTAC **1** (**dBet1**) was the only exception to this trend, possibly due to the very short linker, which could hamper the interaction with metabolism-devoted enzymes. This point will be further discussed in the following paragraphs. The lower half-life values of thalidomide-containing PROTACs suggest that these compounds, in addition to enzymatic transformation, might also undergo partial nonenzymatic degradation during incubation time (the instability of thalidomide was previously shown in Figure 2), but a further analysis of this phenomenon was beyond the scope of this paper. Concerning the PROTAC series containing the AR ligand **46** (compounds **26**–**40** in the Supporting Information Table S1), they generally showed a higher susceptibility to metabolism in cryopreserved human hepatocytes, with all t1/2 values lower than 100 min independent of the linker or the E3 ligase binder used. This trend, associated with the low metabolic stability of AR ligand **46** as previously discussed (Supporting Information Table S2), suggested that the primary site(s) of metabolism in this series is probably related to metabolic liabilities in the compound **46** moiety rather than in the E3 ligase part of the molecules. Therefore, for a deeper understanding of the t1/2 values, the soft spot analysis was performed.

### PROTAC Soft Spot Identification

While metabolic stability data expressed as the half-life of the parent compound represent a valuable parameter to judge the intrinsic stability of a compound,^61^ the identification of soft spots in a molecule is crucial for the rational design of new and more stable compounds.^61^ Although it is now always possible to identify the exact site of metabolism by LC–MS/MS analysis, our study allowed us to devise some general indications, which we believe will be useful in the design of new PROTACs. Soft-spot analysis data are provided in the Supporting Information Table S3.

#### Whole Differs from the Sum of Its Parts

Due to their composed structure, the first natural comparison to make was whether soft spots in a PROTAC could be predicted from the soft spots of the free ligands. As an example, in Table 1, the observed metabolism of compounds **4** and **37**, which are composed of totally different building blocks, is compared with that of the corresponding constituent ligands.

**Table 1:**
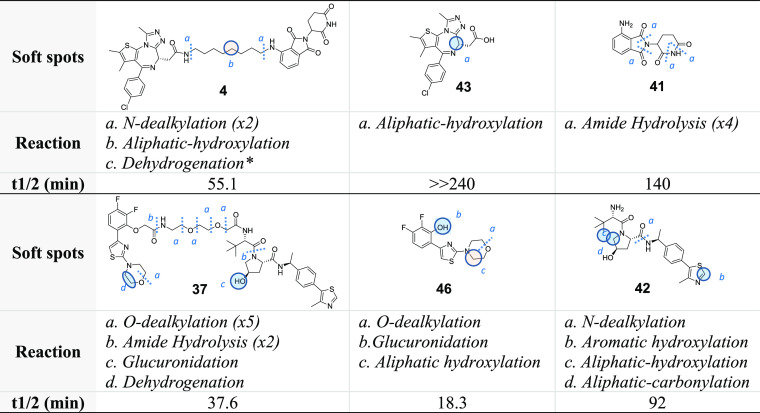
Half-Life for PROTACs and Single Ligand Components upon Incubation in Cryopreserved Human Hepatocytes

Concerning PROTAC **4**, enzymatic degradation of the thalidomide moiety was not observed, although a low metabolic stability of pomalidomide **41** was detected, due to the opening of the phthalimide and glutarimide rings. Similarly, the JQ1 moiety in PROTAC **4** did not undergo the aliphatic hydroxylation observed for ligand **43**, probably due to an increase of the steric hindrance of the JQ1 moiety site of metabolism (however, for other JQ1-based PROTACs, traces of this metabolic route were observed, see the Supporting Information Table S3). Nevertheless, **4** was highly metabolized in cryopreserved human hepatocytes, with the soft spots being identified in the linker and especially in its connection points with the ligands. Concerning PROTAC **37**, the liability of the morpholine ring present in the AR ligand **46** was confirmed, as well as that of two out of four points of the VHL ligand structure **42**. However, the PEG-like linker also played an important role in the enzymatic degradation of **37**, being subjected to *O*-dealkylation and amide hydrolysis reactions. The preserved liability of the AR ligand **46** moiety is also in agreement with what was discussed in the previous paragraph for the AR degrader series (**26**–**40**). In conclusion, translating the well-known Aristotelian concept (“The Whole is Greater than the Sum of its Parts”) to PROTACs’ metabolism, one can derive that “the whole differs from the sum of its parts”. Although this statement may seem trivial, it has a strong impact on medicinal chemists because it confirms that PROTACs represent totally independent chemical entities and that their metabolism cannot be predicted from the one of the ligands used for their design and synthesis. Table 1 also shows that in PROTACs the most labile soft spots are represented by the linker and the chemical connections used to join it to the ligands.

#### Linker Effect

**Figure 3:**
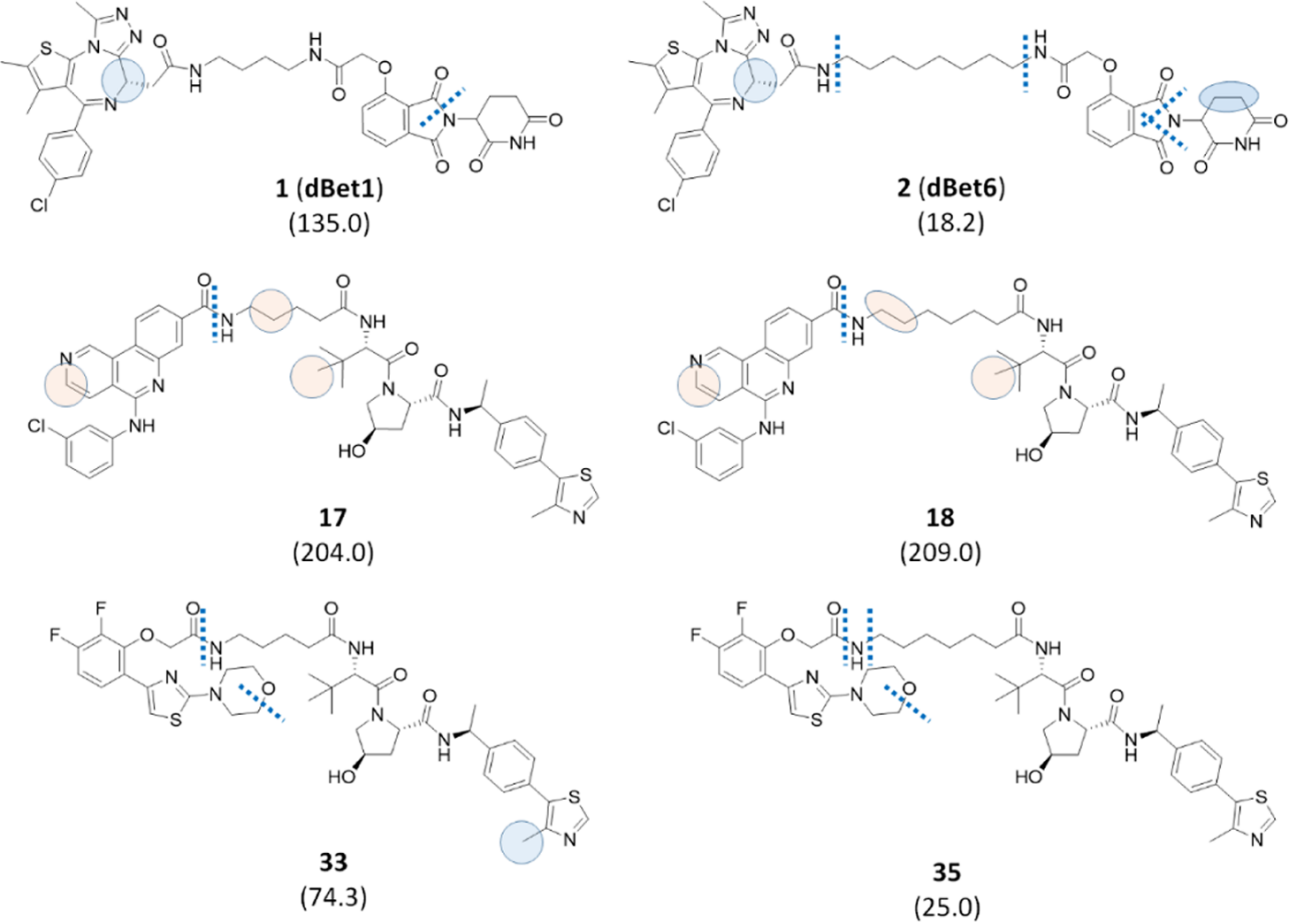
Effect of the length of linear linkers on metabolic stability. The half-life values associated with each compound and expressed in minutes are reported in brackets. Bond cleavages are illustrated as dotted lines, while circles represent atoms subjected to oxidation in a well-defined position by MS/MS fragmentation (blue) or in a defined moiety by MS/MS fragmentation (pink). In the case of pink circles, the displayed position was suggested by MetaSite predictions. Finally, ellipses indicate dehydrogenation reactions, and the same color code for circles was used.

The linker commonly plays an important role in the biological activities and physicochemical properties of PROTACs. With time, the chemical nature of linkers has been variably modified, changing from initial peptide linkers^73^ to (un)saturated alkane or PEG-like chains, to variably functionalized linkers.^74^ In particular, PROTACs containing PEG-based linkers usually display a better solubility profile when compared to those bearing alkyl linkers or even triazole-containing linkers.^75^ Indeed, introducing PEG moieties (possessing a good safety profile)^76^ represents a commonly used strategy for improving pharmaceutical properties of small molecules. Recently, piperidine-containing linkers have also proved to be a good option to improve solubility.^74^ Various bonds have been used to connect a linker to the two ligands, including amide bonds, ether bonds, alkylamines, carbon–carbon bonds, and click-chemistry products.^73^ Finally, the length of the linker has been extensively changed to adapt the PROTAC to its biological function. In fact, on the one hand, if a linker is too short, the simultaneous binding of the two ligands with their targets will be hampered, and the formation of the ternary complex will not occur. On the other hand, if the linker is too long, the PROTAC will not efficiently move the target and the E3 ligase closer to each other, and thus the target protein will not be ubiquitinated.^73,75,77,78^ In the previous section, we discussed an example showing a significant metabolic liability of the linkers (Table 1). Therefore, one may ask how the tailoring of the linker may affect the metabolic stability. We already mentioned that the short linker in PROTAC **1** (**dBet1**) could be a reason for its high metabolic stability. Indeed, the comparison of the t1/2 value of **1** (**dBet1**) with that for **2** (**dBet6**) indicated that the extension of the linker from four to eight methylene units reduces the t1/2 value from 135 to 18.2 min, respectively (Figure 3).

This finding could suggest generalizing that, in the case of linear linkers, a shorter aliphatic linker can be responsible for increased metabolic stability, probably due to steric hindrance of the PROTAC entering into the catalytic site of the metabolic enzymes. The hypothesis was verified comparing two additional PROTACs with a linker composed of four methylene units to long-linker analogues (Figure 3). In particular, the AR-based PROTACs (compounds **33** and **35**, Figure 3) confirmed the expected trend, although with a reduced difference in half-life values possibly due to the reduced length difference between the two linkers (from four to six methylene units). However, replacing the AR ligand unit in compounds **33** and **35** with the CX4945 (**44**) moiety, the increased length of the linear linker did not show the hypothesized effect, with compounds **17** and **18** having equal t1/2 values. Soft spots suggest that, while for the pairs **1**/**2** and **33**/**35** the longer linker seems more prone to the *N*-dealkylation reaction, in the case of **17**/**18** metabolism is very similar. Thus, this and other possible comparisons of data in Table S1 (Supporting Information) suggest that a very short linker can be commonly associated with improved metabolic stability. However, our data show that, in some cases, PROTACs composed of longer linkers could also be metabolically stable.

Similarly, the nature of the linear linker and its binding moiety seem not to heavily affect the metabolic stability. In Table 2, four PROTACs are displayed, containing the AR ligand and pomalidomide moieties as ligands connected by either an aliphatic or a PEG linker of the same length. In compounds **26** and **27**, the hydroxyl thalidomide is linked by an acetamide moiety, while in compounds in **30** and **31**, the linker is directly connected through an amine group. Despite this, compounds **27** and **31** share a similar metabolic stability, compound **30** appears only slightly more stable than the two PEG-containing PROTACs, and compound **26** resulted in being very unstable, with a t1/2 of only 8.4 min. Intriguingly, the greater instability of **26** is not related to different soft spots, if compared to **30** (Table 2), suggesting that the different anchor point between the linker and the thalidomide might modify the affinity with the metabolic enzyme(s). As revealed by the comparison of the two PROTACs with an aliphatic linker, compounds **27** and **31**, both possessing a PEG-like linker, also share a similar soft spot pattern (Table 2), which is mainly due to *O*-dealkylation reactions. It is noteworthy that, although in PEG-like PROTACs the number of soft spots is higher due to the multiple *O*-dealkylation reactions, the overall metabolic rate is not necessarily negatively affected. However, from the medicinal chemistry perspective, a higher number of soft spots make the design of more stable compounds by protection strategies more challenging.

**Table 2:**
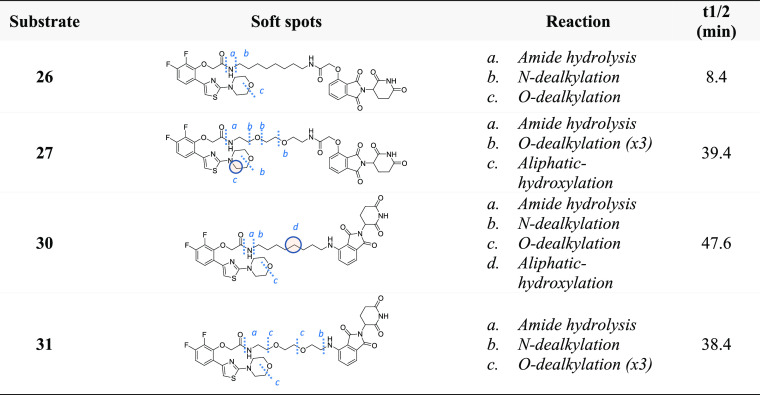
Soft Spots for PROTACs **26**, **27**, **30**, and **31**

**Figure 4:**
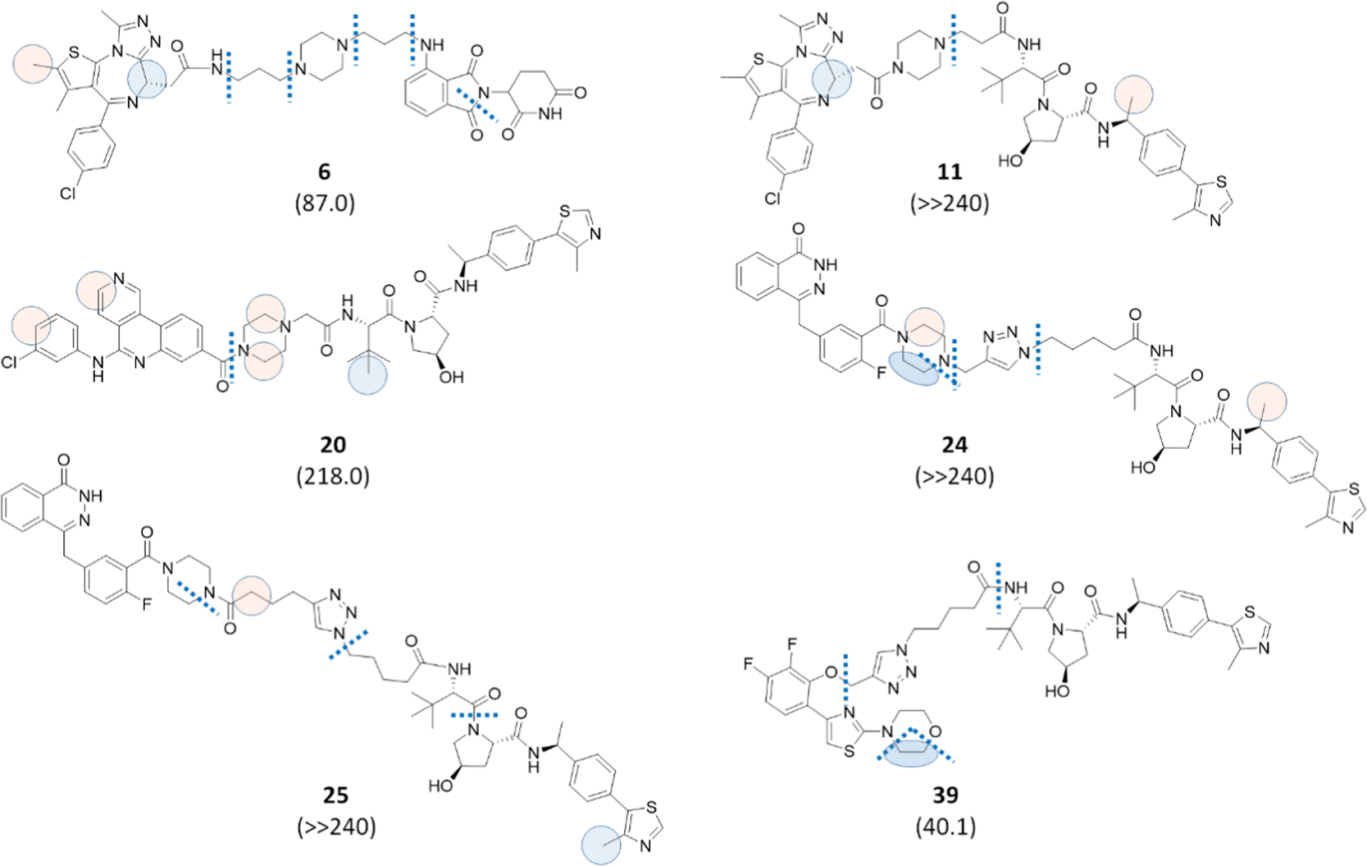
Effect of the cyclic linkers on metabolic stability. The half-life values associated with each compound and expressed in minutes are reported in brackets. Bond cleavages are illustrated as dotted lines, while circles represent atoms subjected to oxidation in a well-defined position by MS/MS fragmentation (blue) or in a defined moiety by MS/MS fragmentation (pink). In the case of pink circles, the displayed position was suggested by MetaSite predictions. Finally, ellipses indicate dehydrogenation reactions, and the same color code for circles was used.

Finally, six PROTACs with various target and E3 ligase binders and bearing cyclic linkers were synthesized to evaluate their effect on metabolic stability. Among them, compounds **6**, **11**, and **20** were characterized by the presence of a piperazine moiety in the linker, while compounds **24**, **25**, and **39** were endowed with a triazole ring. A comparison with similar PROTACs bearing linear linkers (Table S1, Supporting Information) suggested that, with the exception of **39**, the presence of cyclic linkers resulted in a higher metabolic stability. Soft spots for compounds **6**, **11**, **20**, **24**, **25**, and **39** are illustrated in Figure 4. In the BET series (Table S1, entries 1–11 in the Supporting Information), compounds **6** and **11** show higher t1/2 values in the pomalidomide-containing PROTACs and VHL-containing PROTACs, respectively. In addition, the significant increase of stability observed for compound **11** can be explained by the short length of its linker and by the attachment of piperazine to JQ1 through an amide group, which hampers a second *N*-dealkylation reaction (Figure 4). In the CK2 series (Table S1, entries 12–20 in the Supporting Information), VHL-containing compounds with linear linkers **17**–**19** were all endowed with short linkers and were characterized by a high metabolic stability. Therefore, compound **20**, bearing a short piperazine-containing linker, was synthesized and tested, and again this compound turned out to be the most stable in the series, although the t1/2 value was slightly higher than the one for the linear analogue **17** (218 and 207 min, respectively). In the PARP series (Table S1, entries 21–25 in the Supporting Information), a click-chemistry approach was applied to connect the linker to the target binder through a triazole ring to give compounds **24** and **25**. The triazole-containing PROTACs displayed a much greater metabolic stability when compared to their linear analogues (**23** and **22**, respectively). Indeed, as shown in Table S1, t1/2 values for compounds **24** and **25** were greater than 240 min, with about 84% substrate left. Interestingly, an *N*-dealkylation reaction occurred at the triazole, similar to that observed for the piperazine-containing linkers (Figure 4). Finally, as anticipated, compound **39** turned out to be the only PROTAC endowed with a cyclic moiety with a lower metabolic stability when compared with its linear analogue **33**, considering the triazole ring as a bioisosteric substitution of the amide linkage (Table S1, Supporting Information). Among the detected soft spots (Figure 4), the main site of metabolism was localized at the attachment point of the AR ligand, where the occurrence of an *O*-dealkylation reaction is likely favored by the presence of two aromatic moieties nearby.

#### Effect of the Linker’s Site of Attachment

**Figure 5:**
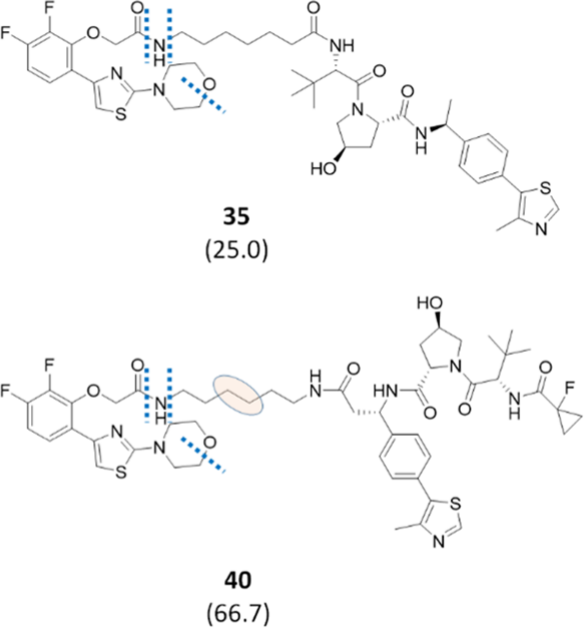
Effect of the linker’s site of attachment on PROTAC stability. Half-life values expressed in minutes are shown in brackets. Bond cleavages are illustrated as dotted lines, while the pink ellipse indicates a dehydrogenation reaction occurring in the linker moiety (the exact position of the soft spot was not elucidated by MS/MS fragmentation, and the displayed position reflects the most probable soft spot according to MetaSite predictions).

In the PROTAC design, the site of attachment of the linker to the ligands is typically selected by analyzing the solvent-exposed areas on ligand-protein binding structures.^73^Figure 5 shows that the selection of the site of attachment might have an impact on the overall metabolic degradation of the PROTAC. Indeed, compound **35** was less stable than **40**, although the identified soft spots were comparable.

### PROTAC Degradation by CYP3A4

**Figure 6:**
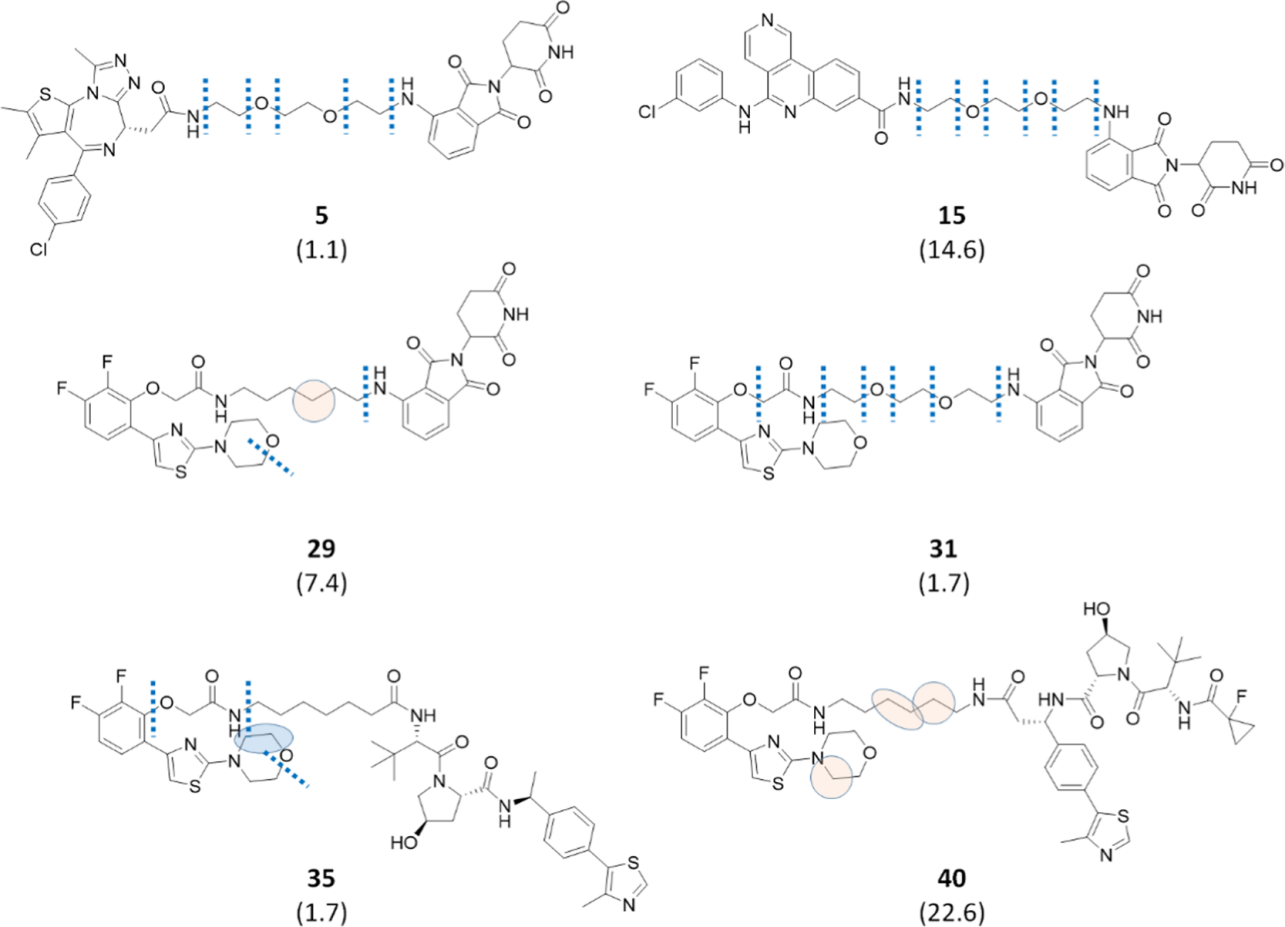
Soft-spot identification of six PROTACs tested for metabolism by CYP3A4. Half-life values expressed in minutes are shown in brackets. Bond cleavages are illustrated as dotted lines. The pink ellipse indicates that a dehydrogenation reaction occurred in the linker. Since the MS/MS fragmentation was not enough to define the exact position of the dehydrogenation’s soft spot, its most probable position was suggested by MetaSite predictions.

CYP3A4 represents a major isozyme in the human liver and is known to metabolize a larger variety of xenobiotics.^44^ An important feature of CYP3A4 is its plasticity, which allows it to accommodate an extensive substrate in its binding site.^45^ Based on these considerations, we assumed that CYP3A4 could be responsible for most of the phase I metabolism observed in the cryopreserved human hepatocyte data. Therefore, six PROTACs with variable combinations of ligands and linkers were selected, and their metabolism by incubation in the presence of recombinant CYP3A4 for 60 min was studied. Figure 6 shows the soft spots detected, while t1/2 values are provided in brackets. In the experimental conditions used (see the Methods section), all tested PROTACs were significantly metabolized, with half-lives well below 30 min. In addition, the already discussed instability at the level of the linker was proved to be caused by CYP3A4.

### PROTAC Degradation by *h*AOX

**Figure 7:**
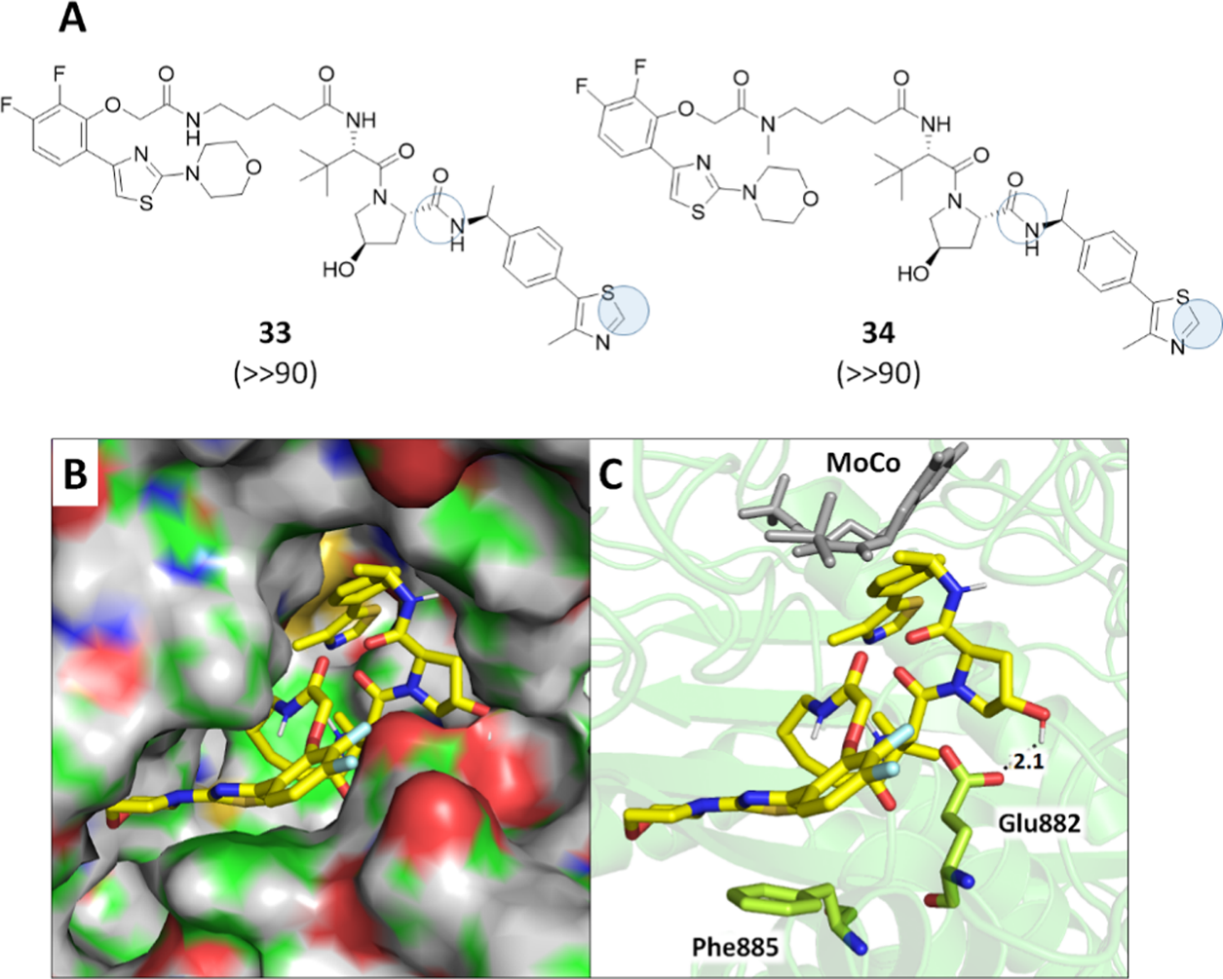
Metabolism of **33** and **34** in human liver cytosol. (A) Soft-spot identification, with filled circles indicating metabolism by *h*AOX and empty circles indicating metabolism by other enzymes. Half-life values expressed in minutes are shown in brackets. (B) Pose of **33** in the *h*AOX cavity according to MetaSite prediction with protein in the surface mode. (C) Pose of **33** in the *h*AOX cavity according to MetaSite prediction highlighting the main interacting residues and the molybdenum pyranopterin cofactor (MoCo).

In addition to CYP-mediated metabolism, increasing importance has been attributed to human aldehyde oxidase (*h*AOX), a cytosolic drug-metabolizing enzyme expressed in the human liver.^12,79−81^ Indeed, strategies designed to reduce CYP-mediated metabolism have resulted in increasing drug reactivity toward AOX.^46,47^ As a consequence, several compounds have failed due to undetected *h*AOX-mediated oxidation (e.g., BIBX1382, RO-1, FK3453, carbazeran).^48,49^ Two reactions are reported to be catalyzed by *h*AOX: (1) the oxidation of a wide range of azaaromatic scaffolds at the unsubstituted carbon in *ortho* to the nitrogen (usually the most electron-deficient);^82^ and (2) the hydrolysis of amides although a few examples have been reported so far.^80,81,83^ Since the PROTACs commonly contain amide groups and heteroaromatic rings, two PROTACs were selected to be screened for *h*AOX metabolism. Compounds **33** and **34** were selected as each one contained three amide groups that might be liability spots for *h*AOX metabolism. In addition, they also contain one 4-aryl or 5-aryl substituted thiazole ring (in the AR ligand moiety (**46**) and the VHL ligand moiety (**42**), respectively). Although five-term moieties are commonly considered not prone to be metabolized by *h*AOX unless it is fused with a phenyl ring to give a benzothiazole,^84−86^ one exception has been reported by Arora et al.,^87^ showing that 2H-oxazoles substituted at the C-4 or C-5 position with variably decorated phenyl rings can undergo oxidation by mouse cytosolic AOX to give the corresponding 2-oxazolones. Therefore, we hypothesized that a similar oxidation pattern could occur in the selected PROTACs although this reaction has not been reported for substituted thiazoles to date. Compounds **33** and **34** were therefore incubated in human liver cytosol for 30, 60, and 90 min in the absence and presence of hydralazine, a selective inhibitor of *h*AOX,^88^ and the kinetics data are illustrated in Figure S2 (Supporting Information). Two reactions occurred for both compounds, the hydrolysis of an amide and an oxidation, both in the VHL ligand moiety (Figure 7A).

While the hydrolysis of the amide was observed also in the presence of hydralazine, indicating that an enzyme other than *h*AOX is responsible for this cleavage, the hydroxylated product was formed only in the absence of the selective inhibitor (Supporting Information Figures S2 and S3). In addition, the MS/MS fragment ion with *m*/*z* 218.0637 revealed that the soft spot for oxidation is located in the 4-methyl-5-phenyl-thiazole moiety that, in its unoxidized form, shows a fragment ion with *m*/*z* of 202.0685 (Supporting Information, Figure S2). Based on the findings by Arora et al.,^87^ it is likely to assume that the metabolites formed for both **33** and **34** are the corresponding 2-thiazolones on the VHL moiety. This finding is noteworthy since VHL is one of the most common E3 ligases exploited for the PROTAC strategy and, therefore, additional larger-scale studies are currently in progress. Finally, MetaSite software was used to generate the most probable binding mode of **33** in the *h*AOX cavity. Figure 7B illustrates that **33** nicely fits the *h*AOX cavity and that the pose exposing the 4-methyl-5-phenyl-thiazole toward the molybdenum pyranopterin cofactor (MoCo) is stabilized by several favored interactions, including a H-bond between the hydroxyl group of the pyrrolidine moiety and Glu882 and a π–π stacking between the thiazole ring in the AR ligand moiety and Phe885. These two residues were recently hypothesized to stabilize other *h*AOX substrates (Figure 7C).^80,82^

## Conclusions

This study represents the first analysis of the metabolic stability of PROTACs applied to a collection of compounds with large chemical variability. Metabolism assays were first performed in cryopreserved human hepatocytes that, containing all hepatic drug-metabolizing enzymes and cofactors at physiological levels, represent the “gold standard” even for the early screening of metabolic stability. Additional studies were conducted to evaluate whether CYP3A4 and *h*AOX could be involved in the observed metabolic biotransformations. A comprehensive analysis of the data in terms of half-life values and soft spot identification allowed us to highlight general trends in PROTAC metabolism. The linker resulted in being the most liable moiety in a PROTAC molecule. Its instability is mainly localized at the attachment points to ligands, involving *N*-dealkylation and amide hydrolysis reactions. Such reactions also occurred in CYP3A4 incubation, indicating that this isoform can play an essential role in PROTAC degradation. In the case of PEG-like linkers, a large number of *O*-dealkylation reactions was observed, indicating that multiple fragmentation points are possible. Nevertheless, the most significant number of soft spots in PEG-like linker-based PROTACs compared to aliphatic-based ones seems not to negatively affect the overall metabolic stability of a compound, with the half-life values being comparable or even better. From the medicinal chemistry perspective, however, the soft spot protection strategies might be more challenging for PEG-like-based PROTACs. The length of the linker also played a role in metabolic stability, with 4-unit linkers being very stable compared to longer ones. Unfortunately, longer linkers are mostly used in PROTAC design for activity optimization, thus limiting the application of this evidence. The use of linkers endowed with cyclic moieties may represent a good strategy to increase metabolic stability; however, we demonstrated that exceptions are possible, as in the case of compound **39**. Concerning the E3 ligands, thalidomide-based PROTACs suffered from nonenzymatic degradation in aqueous solutions. Nevertheless, the protocol developed in this study reduces this degradation during storage in the autosampler for LC–MS analysis. This will not prevent the eventually occurring nonenzymatic hydrolysis of the thalidomide moiety during incubation in cryopreserved human hepatocytes, but it will improve reproducibility of the results. When the VHL ligand is used, we discovered that PROTACs could undergo *h*AOX metabolism at the 5-phenyl-thiazole moiety. This finding not only represents the first evidence of the metabolism on a substituted thiazole by *h*AOX based on our knowledge but also indicates that further studies are required to verify the affinity of the hydroxylated metabolism with VHL aimed at evaluating the impact on PROTAC efficiency. The metabolic degradation of PROTACs by *h*AOX on a large scale is currently under investigation. To conclude, we believe that the study herein reported represents a solid base to start considering metabolism in rational PROTAC design.

## Experimental Section

### Chemistry

#### General

Unless otherwise noted, starting materials, reagents, and solvents were purchased from commercial suppliers and were used as received without further purification.

Compound **41** was purchased from Fluorochem, compound **42** was prepared according to the reported procedure,^52^ while compounds **43** and **44** were purchased from Ambeed and Fluorochem, respectively. PROTACs **26**–**40** were kindly provided by Montelino Therapeutics Inc.

Reactions were routinely monitored by thin-layer chromatography (TLC) performed on a silica gel 60 F_254_ (layer 0.2 mm) precoated aluminum foil (with a fluorescent indicator UV254) (Sigma-Aldrich). Developed plates were air-dried and visualized under UV light (254/365 nm) or using KMnO_4_, ninhydrin, or phosphomolybdic acid stain solutions. Flash column chromatography was performed on Merck silica gel 60 (mesh 230–400). ^1^H NMR and ^13^C NMR spectra were recorded at room temperature at 400 and 101 MHz, respectively, on a Bruker Avance 400 spectrometer using tetramethylsilane (TMS) or residual solvent peak as the internal standard. Chemical shifts are reported in ppm (δ), and the coupling constants (*J*) are given in Hertz (Hz). Peak multiplicities are abbreviated as follows: s (singlet), bs (broad singlet), d (doublet), dd (double doublet), t (triplet), dt (double triplet), q (quartet), p (pentet), and m (multiplet). High-resolution mass spectroscopy (HRMS) analyses were carried out on the Agilent Technologies 6540 UHD Accurate Mass Q-TOF LC–MS system. The purity of all final compounds was confirmed to be >95% by ultraperformance liquid chromatography-tandem mass spectrometry (UPLC-MS). The analyses were carried out according to the method listed below. The mobile phase was a mixture of water (solvent A) and acetonitrile (solvent B), both containing formic acid at 0.1%. Method: Acquity UPLC BEH C18 1.7 μm (C18, 150 mm × 2.1 mm) column at 40 °C using a flow rate of 0.65 mL/min in a 10 min gradient elution. Gradient elution was as follows: 99.5:0.5 (A/B) to 5:95 (A/B) over 8 min, 5:95 (A/B) for 2 min, and then reversion back to 99.5:0.5 (A/B) over 0.1 min. The UV detection is an averaged signal from a wavelength of 190–640 nm, and mass spectra are recorded on a mass spectrometer using positive-mode electrospray ionization. The chemical names were generated using ChemBioDraw 12.0 from CambridgeSoft.

#### General Procedure A: HATU-Mediated Amidation

Under a nitrogen atmosphere, to a stirred solution of the appropriate carboxylic acid (1.0 equiv), suitable amine (1.0 equiv), and DIPEA (4.0 equiv) in dry DMF, HATU (1.25 equiv) was added and the reaction mixture was stirred at room temperature (1–18 h). The mixture was poured in ice-water, yielding a precipitate collected by filtration. When no precipitate formed, the mixture was extracted with EA (×3) and the reunited organic phases were washed with water (×3) and brine (×3), dried over Na_2_SO_4_, and evaporated to dryness. The crude was purified as described below.

##### 2-((*S*)-4-(4-Chlorophenyl)-2,3,9-trimethyl-6*H*-thieno[3,2-*f*][1,2,4]triazolo[4,3-*a*][1,4]diazepin-6-yl)-*N*-(8-((2-(2,6-dioxopiperidin-3-yl)-1,3-dioxoisoindolin-4-yl)amino)octyl)acetamide (**4**)^51^

General Procedure A (1 h) was followed using **43** (0.021 g, 0.053 mmol) and **47** (0.023 g, 0.053 mmol) to afford the title compound as a yellow solid (0.019 g, 45% yield) after purification by flash column chromatography on SiO_2_ (DCM/acetone/MeOH, 92:4:4). ^1^H NMR (400 MHz, DMSO-*d*_6_) δ 11.09 (s, 1H), 8.15 (t, *J* = 5.3 Hz, 1H), 7.61–7.53 (m, 1H), 7.48 (d, *J* = 8.5 Hz, 2H), 7.42 (d, *J* = 8.4 Hz, 2H), 7.07 (d, *J* = 8.7 Hz, 1H), 7.01 (d, *J* = 7.1 Hz, 1H), 6.52 (t, *J* = 5.9 Hz, 1H), 5.04 (dd, *J* = 13.0, 5.3 Hz, 1H), 4.53–4.44 (m, 1H), 3.31–3.01 (m, 7H), 2.93–2.81 (m, 1H), 2.63–2.55 (m, 4H), 2.40 (s, 3H), 2.06–1.97 (m, 1H), 1.61 (s, 3H), 1.59–1.49 (m, 2H), 1.49–1.38 (m, 2H), 1.32 (d, *J* = 25.3 Hz, 8H). ^13^C NMR (101 MHz, DMSO-*d*_6_) δ 173.26, 170.55, 169.77, 169.41, 167.77, 163.42, 155.60, 150.25, 146.90, 137.19, 136.73, 135.70, 132.73, 132.66, 131.17, 130.58 (2C), 130.25, 130.03, 128.90 (2C), 117.62, 110.83, 109.49, 54.41, 49.01, 42.30, 38.88, 38.16, 31.45, 29.71, 29.22, 29.16, 26.81, 26.76, 22.62, 14.51, 13.14, 11.76. HRMS (ESI) *m*/*z* [M + H]+ calcd for C_40_H_43_ClN_8_O_5_S 783.28384, found 783.28533. UPLC retention time: 5.943 min.

##### 2-((*S*)-4-(4-Chlorophenyl)-2,3,9-trimethyl-6*H*-thieno[3,2-*f*][1,2,4]triazolo[4,3-*a*][1,4]diazepin-6-yl)-N-(2-(2-(2-((2-(2,6-dioxopiperidin-3-yl)-1,3-dioxoisoindolin-4-yl)amino)ethoxy)ethoxy)ethyl)acetamide (**5**)^89^

General Procedure A (1 h) was followed using **43** (0.023 g, 0.057 mmol) and **48**(90) (0.025 g, 0.057 mmol) to afford the title compound as a yellow solid (0.013 g, 29% yield) after purification by flash column chromatography on SiO_2_ (DCM/acetone/MeOH, 92:3:5). ^1^H NMR (400 MHz, DMSO-*d*_6_) δ 11.09 (s, 1H), 8.26 (t, *J* = 5.2 Hz, 1H), 7.58 (t, *J* = 7.8 Hz, 1H), 7.48 (d, *J* = 8.4 Hz, 2H), 7.42 (d, *J* = 8.4 Hz, 2H), 7.14 (d, *J* = 8.6 Hz, 1H), 7.03 (d, *J* = 7.0 Hz, 1H), 6.62 (t, *J* = 5.4 Hz, 1H), 5.06 (dd, *J* = 12.8, 5.2 Hz, 1H), 4.50 (t, *J* = 6.9 Hz, 1H), 3.69–3.40 (m, 10H), 3.31–3.21 (m, 5H), 2.92–2.80 (m, 1H), 2.59 (s, 3H), 2.41 (s, 3H), 2.07–1.97 (m, 1H), 1.62 (s, 3H), 1.30–1.22 (m, *J* = 8.4 Hz, 1H). ^13^C NMR (101 MHz, DMSO-*d*_6_) δ 173.26, 170.55, 170.12, 169.39, 167.76, 163.46, 155.57, 150.28, 146.86, 137.23, 136.68, 135.67, 132.73, 132.56, 131.15, 130.61 (2C), 130.29, 130.01, 128.91 (2C), 117.90, 111.12, 109.72, 70.18, 70.10, 69.71, 69.37, 54.29, 49.01, 42.17, 39.09, 37.97, 31.44, 22.60, 14.52, 13.14, 11.77. HRMS (ESI) *m*/*z* [M + H]+ calcd for C_38_H_39_ClN_8_O_7_S 787.24237, found 787.24302. UPLC retention time: 5.053 min.

##### 4-((3-(4-(3-Aminopropyl)piperazin-1-yl)propyl)amino)-2-(2,6-dioxopiperidin-3-yl)isoindoline-1,3-dione Hydrochloride (**49**)

Under a nitrogen atmosphere, a solution of **62** (0.246 g, 0.891 mmol), *tert*-butyl (3-(4-(3-aminopropyl)piperazin-1-yl)propyl)carbamate^91^ (0.295 g, 0.979 mmol), and DIPEA (0.3 mL, 1.780 mmol) in dry DMSO (2.0 mL) was stirred at 60 °C for 3 h. After cooling to room temperature, the reaction mixture was poured in ice-water and extracted with EA (×3). The reunited organic phases were washed with brine, dried over Na_2_SO_4_, and evaporated to dryness. The crude residue was purified by flash column chromatography on SiO_2_ (DCM/MeOH, 97:3) to give *tert*-butyl(3-(4-(3-((2-(2,6-dioxopiperidin-3-yl)-1,3-dioxoisoindolin-4yl)amino) propyl)piperazin-1-yl)propyl)carbamate (0.085 g, 56% yield) as a yellow oil. ^1^H NMR (400 MHz, CDCl_3_) δ 8.70 (bs, 1H), 7.48 (t, *J* = 7.8 Hz, 1H), 7.08 (d, *J* = 7.1 Hz, 1H), 6.91 (d, *J* = 8.5 Hz, 1H), 6.77 (bs, 1H), 5.35 (bs, 1H), 4.91 (dd, *J* = 12.0, 5.4 Hz, 1H), 3.43–3.29 (m, 2H), 3.26–3.13 (m, 2H), 2.93–2.36 (m, 14H), 2.19–2.06 (m, 1H), 1.92–1.59 (m, 5H), 1.44 (s, 9H). HRMS (ESI) *m*/*z* [M + H]+ calcd for C_28_H_40_N_6_O_6_ 557.30876, found 557.31024. UPLC retention time: 2.993 min.

Then, the solution of the obtained compound (0.078 g, 0.140 mmol) in 4.0N HCl in dioxane (0.78 mL) was stirred at room temperature overnight. The solvent was evaporated to dryness, and the solid was tritured with diethyl ether (DEE) and collected by filtration, yielding **49** as a white solid (0.068 g, 99% yield). ^1^H NMR (400 MHz, DMSO-*d*_6_) δ 11.92 (bs, 2H), 11.11 (s, 1H), 8.04 (bs, 3H), 7.61 (t, *J* = 7.8 Hz, 1H), 7.19 (d, *J* = 8.6 Hz, 1H), 7.06 (d, *J* = 7.0 Hz, 1H), 6.79 (bs, 1H), 5.06 (dd, *J* = 12.7, 5.3 Hz, 1H), 3.90–3.47 (m, 8H), 3.37–3.02 (m, 8H), 2.97–2.83 (m, 3H), 2.70–2.54 (m, 1H), 2.10–1.92 (m, 4H). HRMS (ESI) *m*/*z* [M + H]+ calcd for C_23_H_32_N_6_O_4_ 457.25633, found 457.25670. UPLC retention time: 1.78 min.

##### 2-((*S*)-4-(4-Chlorophenyl)-2,3,9-trimethyl-6*H*-thieno[3,2-*f]*[1,2,4]triazolo[4,3-*a*][1,4]diazepin-6-yl)-*N*-(3-(4-(3-((2-(2,6-dioxopiperidin-3-yl)-1,3-dioxoisoindolin-4-yl)amino)propyl)piperazin-1-yl)propyl)acetamide (**6**)

General Procedure A (overnight) was followed using **49** (0.069 g, 0.140 mmol) and **43** (0.055 g, 0.140 mmol) to afford the title compound as a fluorescent-yellow solid (0.023 g, 20% yield) after purification by flash column chromatography on SiO_2_ (DCM/MeOH, 95:5). ^1^H NMR (400 MHz, DMSO-*d*_6_) δ 11.10 (s, 1H), 8.29 (bs, 1H), 7.59 (t, *J* = 7.8 Hz, 1H), 7.50 (d, *J* = 8.1 Hz, 2H), 7.43 (d, *J* = 8.3 Hz, 2H), 7.13 (d, *J* = 8.6 Hz, 1H), 7.04 (d, *J* = 7.1 Hz, 1H), 6.81 (bs, 1H), 5.06 (dd, *J* = 13.0, 5.2 Hz, 1H), 4.51 (t, *J* = 7.0 Hz, 1H), 3.43–3.35 (m, 3H), 3.30 (s, 2H), 3.28–3.02 (m, 6H), 2.94–2.84 (m, 2H), 2.65–2.53 (m, 5H), 2.42 (s, 3H), 2.08–2.01 (m, 1H), 1.86–1.67 (m, 3H), 1.63 (s, 3H). HRMS (ESI) *m*/*z* [M + H]+ calcd for C_42_H_47_ClN_10_O_5_S 839.32184, found 839.32312. UPLC retention time: 4.118 min.

##### (2*S*,4*R*)-1-((*S*)-2-(7-(2-((*S*)-4-(4-Chlorophenyl)-2,3,9-trimethyl-6*H*-thieno[3,2-*f*][1,2,4]triazolo[4,3-*a*][1,4]diazepin-6-yl)acetamido)heptanamido)-3,3-dimethylbutanoyl)-4-hydroxy-*N*-((*S*)-1-(4-(4-methylthiazol-5-yl)phenyl)ethyl)pyrrolidine-2-carboxamide (**8**)

General Procedure A (2 h) was followed using **43** (0.023 g, 0.057 mmol) and **50** (0.035 g, 0.057 mmol) to afford the title compound as a white solid (0.024 g, 43% yield) after purification by flash column chromatography on SiO_2_ (DCM/MeOH, 97:3 to 95:5). ^1^H NMR (400 MHz, DMSO-*d*_6_) δ 8.98 (s, 1H), 8.36 (d, *J* = 7.7 Hz, 1H), 8.15 (t, *J* = 5.4 Hz, 1H), 7.79 (d, *J* = 9.2 Hz, 1H), 7.49 (d, *J* = 8.6 Hz, 2H), 7.42 (dd, *J* = 8.1, 5.0 Hz, 4H), 7.37 (d, *J* = 8.2 Hz, 2H), 5.09 (d, *J* = 3.5 Hz, 1H), 4.96–4.85 (m, 1H), 4.56–4.47 (m, 2H), 4.43 (t, *J* = 7.9 Hz, 1H), 4.27 (s, 1H), 3.60 (s, 2H), 3.26–3.02 (m, 4H), 2.59 (s, 3H), 2.45 (s, 3H), 2.41 (s, 3H), 2.30–2.20 (m, 1H), 2.16–2.06 (m, 1H), 2.04–1.95 (m, 1H), 1.84–1.75 (m, 1H), 1.61 (s, 3H), 1.53–1.40 (m, 4H), 1.37 (d, *J* = 7.0 Hz, 3H), 1.32–1.24 (m, 4H), 0.93 (s, 9H). ^13^C NMR (101 MHz, DMSO-*d*_6_) δ 172.50, 171.09, 170.09, 169.77, 163.47, 155.59, 150.28, 148.22, 145.12, 137.24, 135.71, 132.72, 131.58, 131.17, 130.57 (2C), 130.29, 130.16, 130.05, 129.28 (2C), 128.93 (2C), 126.84 (2C), 126.74, 69.22, 59.00, 56.79, 56.70, 54.39, 48.16, 38.95, 38.19, 38.12, 35.64, 35.32, 29.60, 28.87, 26.92 (3C), 26.61, 25.85, 22.90, 16.45, 14.51, 13.15, 11.75. HRMS (ESI) *m*/*z* [M + H]+ calcd for C_49_H_60_ClN_9_O_5_S_2_ 954.39201, found 954.39400. UPLC retention time: 5.427 min.

##### (2*S*,4*R*)-1-((*S*)-2-(*tert*-Butyl)-14-((*S*)-4-(4-chlorophenyl)-2,3,9-trimethyl-6*H*-thieno[3,2-*f*][1,2,4]triazolo[4,3-*a*][1,4]diazepin-6-yl)-4,13-dioxo-6,9-dioxa-3,12-diazatetradecanoyl)-4-hydroxy-*N*-((*S*)-1-(4-(4-methylthiazol-5-yl)phenyl)ethyl)pyrrolidine-2-carboxamide (**9**)

General Procedure A (2 h) was followed using **43** (0.056 g, 0.14 mmol) and **51** (0.089 g, 0.014 mmol) to afford the title compound as a white solid (0.025 g, 18% yield) after purification by flash column chromatography on SiO_2_ (DCM/MeOH 95:5). ^1^H NMR (400 MHz, DMSO-*d*_6_) δ 8.98 (s, 1H), 8.44 (d, *J* = 7.6 Hz, 1H), 8.27 (t, *J* = 5.7 Hz, 1H), 7.49 (d, *J* = 8.6 Hz, 2H), 7.46–7.38 (m, 5H), 7.36 (d, *J* = 8.3 Hz, 2H), 5.14 (d, *J* = 3.5 Hz, 1H), 4.96–4.86 (m, 1H), 4.57 (d, *J* = 9.6 Hz, 1H), 4.54–4.48 (m, 1H), 4.46 (d, *J* = 8.0 Hz, 1H), 4.29 (s, 1H), 3.98 (s, 2H), 3.60 (dd, *J* = 17.0, 12.0 Hz, 6H), 3.54–3.46 (m, 2H), 3.31–3.19 (m, 4H), 2.60 (s, 3H), 2.45 (s, 3H), 2.41 (s, 3H), 2.11–2.00 (m, 1H), 1.84–1.75 (m, 1H), 1.63 (s, 3H), 1.35 (d, *J* = 7.0 Hz, 3H), 0.95 (s, 9H). ^13^C NMR (101 MHz, DMSO-*d*_6_) δ 170.90, 170.13, 169.53, 169.03, 163.42, 155.61, 150.27, 148.21, 145.14, 137.23, 135.68, 132.70, 131.57, 131.17, 130.61 (2C), 130.30, 130.15, 130.07, 129.36, 129.27 (2C), 128.93 (2C), 126.79 (2C), 126.74, 70.90, 70.05, 69.88, 69.79, 69.24, 59.02, 57.01, 56.17, 54.28, 48.23, 39.07, 38.20, 37.92, 36.69, 36.23, 26.71 (3C), 22.87, 16.45, 14.51, 13.15, 11.75. HRMS (ESI) *m*/*z* [M + H]+ calcd for C_48_H_58_ClN_9_O_7_S_2_ 972.36619, found 972.36785. UPLC retention time: 5.344 min.

##### (2*S*,4*R*)-1-((*S*)-3,3-Dimethyl-2-(3-(piperazin-1-yl)propanamido)butanoyl)-4-hydroxy-*N*-((*S*)-1-(4-(4-methylthiazol-5-yl)phenyl)ethyl)pyrrolidine-2-carboxamide Hydrochloride (**52**)

Under a nitrogen atmosphere, a solution of **42** (0.200 g, 0.416 mmol), 3-(1-*tert*-butoxycarbonylpiperazin-4-yl)propionic acid (0.124 g, 0.458 mmol), HATU (0.209 g, 0.520 mmol), and DIPEA (0.3 mL, 1.664 mmol) in dry DMF (2.0 mL) was stirred at room temperature for 1 h. Then, the reaction mixture was poured in ice-water and extracted with EA (×3). The reunited organic phases were washed with water (×2) and brine, dried over Na_2_SO_4_, and evaporated to dryness. The crude residue was purified by flash column chromatography on SiO_2_ (DCM/acetone/MeOH, 75:20:5) to give *tert*-butyl-4-(3-(((*S*)-1-((2*S*,4*R*)-4-hydroxy-2-(((*S*)-1-(4-(4-methylthiazol-5-yl)phenyl)ethyl)carbamoyl)pyrrolidin-1-yl)-3,3-dimethyl-1-oxobutan-2-yl)amino)-3-oxopropyl)piperazine-1-carboxylate (0.145 g, 51% yield) as a colorless oil. ^1^H NMR (400 MHz, CDCl_3_) δ 9.02 (bs, 1H), 8.67 (s, 1H), 7.57 (d, *J* = 7.9 Hz, 1H), 7.56–7.30 (m, 4H), 5.13–5.01 (m, 1H), 4.78 (t, *J* = 8.0 Hz, 1H), 4.47 (s, 1H), 4.44–4.36 (m, 1H), 4.21 (d, *J* = 11.2 Hz, 1H), 3.74–3.14 (m, 6H), 2.91–2.30 (m, 11H), 2.14–2.04 (m, 1H), 1.78–1.63 (m, 1H), 1.53–1.41 (m, 12H), 1.07 (s, 9H). HRMS (ESI) *m*/*z* [M + H]+ calcd for C_35_H_52_N_6_O_6_S 685.37473, found 685.37551. UPLC retention time: 3.716 min.

Then, the solution of the obtained compound (0.140 g, 0.204 mmol) in 4.0N HCl in dioxane (1.5 mL) was stirred at room temperature for 2 h. The solvent was evaporated to dryness, and the solid was tritured with DEE and collected by filtration, yielding **52** as a white solid (0.124 g, 98% yield). ^1^H NMR (400 MHz, MeOD) δ 9.86 (s, 1H), 7.68–7.46 (m, 4H), 5.09–4.99 (m, 1H), 4.66–4.53 (m, 2H), 4.50–4.38 (m, 1H), 3.96 (d, *J* = 10.9 Hz, 1H), 3.82–3.47 (m, 12H), 2.93 (t, *J* = 6.5 Hz, 2H), 2.61 (s, 3H), 2.25 (dd, *J* = 12.8, 7.8 Hz, 1H), 2.01–1.90 (m, 1H), 1.67–1.47 (m, 3H), 1.18–0.95 (m, 9H). HRMS (ESI) *m*/*z* [M + H]+ calcd for C_30_H_44_N_6_O_4_S 585.32230, found 585.32540. UPLC retention time: 2.852 min.

##### (2*S*,4*R*)-1-((*S*)-2-(3-(4-(2-((*S*)-4-(4-Chlorophenyl)-2,3,9-trimethyl-6*H*-thieno[3,2-*f*][1,2,4]triazolo[4,3-*a*][1,4]diazepin-6-yl)acetyl)piperazin-1-yl)propanamido)-3,3-dimethylbutanoyl)-4-hydroxy-*N*-((*S*)-1-(4-(4-methylthiazol-5-yl)phenyl)ethyl)pyrrolidine-2-carboxamide (**11**)

General Procedure A (2 h) was followed using **52** (0.062 g, 0.099 mmol) and **43** (0.040 g, 0.099 mmol) to afford the title compound as a white solid (0.020 g, 21% yield) after purification by flash column chromatography on SiO_2_ (DCM/MeOH, 93:7). ^1^H NMR (400 MHz, DMSO-*d*_6_) δ 8.98 (s, 1H), 8.40 (t, *J* = 8.5 Hz, 2H), 7.52–7.35 (m, 8H), 5.13 (d, *J* = 3.0 Hz, 1H), 4.99–4.86 (m, 1H), 4.61–4.53 (m, 2H), 4.45 (t, *J* = 7.9 Hz, 1H), 4.29 (s, 1H), 3.78–3.55 (m, 5H), 3.55–3.46 (m, 2H), 3.46–3.36 (m, 2H), 2.71–2.53 (m, 5H), 2.48–2.39 (m, 8H), 2.38–2.23 (m, 2H), 2.09–2.98 (m, 1H), 1.85–1.74 (m, 1H), 1.63 (s, 3H), 1.38 (d, *J* = 6.9 Hz, 3H), 0.97 (s, 9H). ^13^C NMR (101 MHz, DMSO-*d*_6_) δ 171.26, 171.06, 169.99, 168.54, 163.31, 155.71, 150.22, 148.20, 145.16, 137.22, 135.64, 132.65, 131.57, 131.13, 130.61 (2C), 130.33, 130.13, 130.09, 129.33, 129.27 (2C), 128.93(2C), 126.82 (2C), 69.22, 58.98, 56.71, 54.63, 54.37, 52.99, 52.64, 48.20, 45.43, 41.63, 38.22, 35.92 (2C), 35.19, 32.85, 26.92 (3C), 22.92, 16.45, 14.49, 13.16, 11.75. HRMS (ESI) *m*/*z* [M + H]+ calcd for C_49_H_59_ClN_10_O_5_S_2_ 967.38781, found 967.38655. UPLC retention time: 4.492 min

##### 5-((3-Chlorophenyl)amino)-*N*-(8-(2-((2-(2,6-dioxopiperidin-3-yl)-1,3-dioxoisoindolin-4-yl)oxy)acetamido)octyl)benzo[*c*][2,6]naphthyridine-8-carboxamide (**12**)

General Procedure A (overnight) was followed using **44** (0.028 g, 0.081 mmol) and **53** (0.040 g, 0.081 mmol) to afford the title compound as a yellow solid (0.027 g, 42% yield) after purification by flash column chromatography on SiO_2_ (DCM/MeOH 95:5 to 93:7). ^1^H NMR (400 MHz, DMSO-*d*_6_) δ 11.11 (s, 1H), 10.19 (s, 1H), 9.66 (s, 1H), 8.98 (d, *J* = 5.6 Hz, 1H), 8.84 (d, *J* = 8.5 Hz, 1H), 8.73 (t, *J* = 5.3 Hz, 1H), 8.58 (d, *J* = 5.6 Hz, 1H), 8.27 (s, 1H), 8.22 (s, 1H), 8.10 (d, *J* = 8.1 Hz, 1H), 7.99–7.89 (m, 2H), 7.79 (t, *J* = 7.9 Hz, 1H), 7.53–7.41 (m, 2H), 7.37 (d, *J* = 8.5 Hz, 1H), 7.14 (d, *J* = 7.9 Hz, 1H), 5.11 (dd, *J* = 12.9, 5.3 Hz, 1H), 4.75 (s, 2H), 3.31–3.26 (m, 2H), 3.21–3.06 (m, 2H), 2.97–2.82 (m, 1H), 2.69–2.52 (m, 2H), 2.11–1.96 (m, 1H), 1.63–1.51 (m, 2H), 1.48–1.38 (m, 2H), 1.38–1.19 (m, 8H). ^13^C NMR (101 MHz, DMSO-*d*_6_) δ 173.23, 170.34, 167.19, 167.06, 166.11, 165.96, 155.50, 150.54, 148.18, 147.72, 143.78, 142.39, 137.36, 136.16, 133.48, 133.27, 130.58, 127.56, 126.25, 124.19, 123.39, 122.78, 122.68, 121.44, 120.82, 120.70, 119.70, 117.27, 116.83, 116.49, 68.09, 49.27, 38.79 (2C), 31.41, 29.57, 29.45, 29.22, 29.16, 26.97, 26.75, 22.46. HRMS (ESI) *m*/*z* [M + H]+ calcd for C_42_H_40_ClN_7_O_7_ 790.27560, found 790.27511. UPLC retention time: 5.247 min.

##### 5-((3-Chlorophenyl)amino)-*N*-(10-(2-((2-(2,6-dioxopiperidin-3-yl)-1,3-dioxoisoindolin-4-yl)oxy)acetamido)decyl)benzo[*c*][2,6]naphthyridine-8-carboxamide (**13**)

General Procedure A (overnight) was followed using **44** (0.019 g, 0.053 mmol) and **54** (0.028 g, 0.053 mmol) to afford the title compound as a yellow solid (0.023 g, 51% yield) after purification by flash column chromatography on SiO_2_ (DCM/MeOH 95:5). ^1^H NMR (400 MHz, DMSO-*d*_6_) δ 11.11 (s, 1H), 10.19 (s, 1H), 9.65 (s, 1H), 8.98 (d, *J* = 5.6 Hz, 1H), 8.84 (d, *J* = 8.8 Hz, 1H), 8.73 (t, *J* = 5.6 Hz, 1H), 8.58 (d, *J* = 5.7 Hz, 1H), 8.28 (s, 1H), 8.22 (s, 1H), 8.09 (d, *J* = 8.3 Hz, 1H), 7.96–7.87 (m, 2H), 7.82–7.75 (m, 1H), 7.48 (d, *J* = 7.3 Hz, 1H), 7.44 (t, *J* = 8.0 Hz, 1H), 7.37 (d, *J* = 8.7 Hz, 1H), 7.14 (d, *J* = 7.5 Hz, 1H), 5.11 (dd, *J* = 12.8, 5.4 Hz, 1H), 4.75 (s, 2H), 3.32–3.26 (m, 2H), 3.18–3.07 (m, 2H), 2.96–2.83 (m, 1H), 2.67–2.51 (m, 2H), 2.11–1.98 (m, 1H), 1.61–1.52 (m, 2H), 1.46–1.37 (m, 2H), 1.36–1.16 (m, 12H). ^13^C NMR (101 MHz, DMSO-*d*_6_) δ 173.22, 170.33, 167.18, 167.05, 166.11, 165.96, 155.50, 150.53, 148.18, 147.72, 143.77, 142.39, 137.36, 136.17, 133.48, 133.27, 130.57, 127.56, 126.25, 124.19, 123.38, 122.77, 122.67, 121.44, 120.82, 120.69, 119.68, 117.27, 116.82, 116.49, 68.10, 49.27, 38.78 (2C), 31.42, 29.57, 29.44 (3C), 29.26, 29.18, 27.01, 26.77, 22.46. HRMS (ESI) *m*/*z* [M + Na]+ calcd for C_44_H_44_ClN_7_O_7_ 840.28830, found 840.28881. UPLC retention time: 5.71 min.

##### 5-((3-Chlorophenyl)amino)-*N*-(1-((2-(2,6-dioxopiperidin-3-yl)-1,3-dioxoisoindolin-4-yl)oxy)-2-oxo-6,9,12-trioxa-3-azatetradecan-14-yl)benzo[*c*][2,6]naphthyridine-8-carboxamide (**14**)

General Procedure A (overnight) was followed using **44** (0.014 g, 0.040 mmol) and **55** (0.022 g, 0.040 mmol) to afford the title compound as a yellow solid (0.014 g, 42% yield) after purification by flash column chromatography on SiO_2_ (DCM/MeOH 98:2 to 95:5). ^1^H NMR (400 MHz, DMSO-*d*_6_) δ 11.12 (s, 1H), 10.19 (s, 1H), 9.66 (s, 1H), 8.99 (d, *J* = 5.5 Hz, 1H), 8.89–8.76 (m, 2H), 8.58 (d, *J* = 5.6 Hz, 1H), 8.28 (s, *J* = 13.7 Hz, 1H), 8.24 (s, 1H), 8.11 (d, *J* = 8.0 Hz, 1H), 8.03–7.90 (m, 2H), 7.78 (t, *J* = 7.9 Hz, 1H), 7.51–7.40 (m, 2H), 7.37 (d, *J* = 8.5 Hz, 1H), 7.15 (d, *J* = 7.6 Hz, 1H), 5.11 (dd, *J* = 12.8, 5.1 Hz, 1H), 4.76 (s, 2H), 3.64–3.38 (m, 12H), 3.32–3.24 (m, 4H), 2.99–2.80 (m, 1H), 2.69–2.54 (m, 2H), 2.11–1.95 (m, 1H). ^13^C NMR (101 MHz, DMSO-*d*_6_) δ 173.21, 170.32, 167.30, 167.15, 166.31, 165.85, 155.38, 150.54, 148.17, 147.74, 143.75, 142.36, 137.34, 135.81, 133.45, 133.26, 130.55, 127.52, 126.33, 124.20, 123.35, 122.80, 122.67, 121.53, 120.73, 120.66, 119.67, 117.17, 116.81, 116.45, 70.23, 70.21, 70.08 (3C), 69.31, 69.26, 67.91, 49.25, 38.85, 31.41, 22.45. HRMS (ESI) *m*/*z* [M + Na]+ calcd for C_42_H_40_ClN_7_O_10_ 860.24174, found 860.24314. UPLC retention time: 4.355 min.

##### 5-((3-Chlorophenyl)amino)-*N*-(2-(2-(2-((2-(2,6-dioxopiperidin-3-yl)-1,3-dioxoisoindolin-4-yl)amino)ethoxy)ethoxy)ethyl)benzo[*c*][2,6]naphthyridine-8-carboxamide (**15**)

General Procedure A (1 h) was followed using **44** (0.020 g, 0.057 mmol) and **48**(90) (0.028 g, 0.057 mmol) to afford the title compound as a yellow solid (0.024 g, 57% yield) after purification by flash column chromatography on SiO_2_ (DCM/MeOH, 97:3). ^1^H NMR (400 MHz, DMSO-*d*_6_) δ 11.08 (s, 1H), 10.16 (s, 1H), 9.65 (s, 1H), 8.98 (d, *J* = 5.4 Hz, 1H), 8.81 (d, *J* = 8.4 Hz, 1H), 8.77 (t, *J* = 5.6 Hz, 1H), 8.57 (d, *J* = 5.3 Hz, 1H), 8.27 (s, 1H), 8.23 (s, 1H), 8.09 (d, *J* = 8.8 Hz, 1H), 7.93 (d, *J* = 8.6 Hz, 1H), 7.45 (q, *J* = 8.0 Hz, 2H), 7.14 (d, *J* = 7.4 Hz, 1H), 7.01 (d, *J* = 8.6 Hz, 1H), 6.92 (d, *J* = 7.1 Hz, 1H), 6.53 (t, *J* = 5.8 Hz, 1H), 5.02 (dd, *J* = 13.1, 5.4 Hz, 1H), 3.70–3.55 (m, 8H), 3.54–3.45 (m, 2H), 3.45–3.36 (m, 2H), 3.17 (d, *J* = 5.3 Hz, 1H), 2.93–2.80 (m, 1H), 2.62–2.53 (m, 1H), 2.07–1.97 (m, 1H). ^13^C NMR (101 MHz, DMSO-*d*_6_) δ 173.24, 170.54, 169.32, 167.67, 166.30, 150.53, 148.16, 147.73, 146.73, 143.74, 142.38, 136.49, 135.81, 133.26, 132.43, 130.55, 127.51, 126.30, 124.19, 123.33, 122.76, 122.66, 121.51, 120.67, 119.67, 117.70, 116.81, 110.98, 109.63, 70.18, 70.12, 69.33 (3C), 48.98, 42.15, 31.44, 22.58. HRMS (ESI) *m*/*z* [M + H]+ calcd for C_38_H_34_ClN_7_O_7_ 736.22865, found 736.22880. UPLC retention time: 4.820 min.

##### 5-((3-Chlorophenyl)amino)-*N*-(6-((2-(2,6-dioxopiperidin-3-yl)-1,3-dioxoisoindolin-4-yl)amino)hexyl)benzo[*c*][2,6]naphthyridine-8-carboxamide (**16**)

General Procedure A (2 h) was followed using **44** (0.011 g, 0.032 mmol) and **56** (0.013 g, 0.032 mmol) to afford the title compound as a yellow solid (0.012 g, 53% yield) after purification by flash column chromatography on SiO_2_ (DCM/MeOH, 97:3). ^1^H NMR (400 MHz, DMSO-*d*_6_) δ 11.08 (s, 1H), 10.19 (s, 1H), 9.66 (s, 1H), 8.99 (d, *J* = 5.6 Hz, 1H), 8.85 (d, *J* = 8.6 Hz, 1H), 8.74 (t, *J* = 5.4 Hz, 1H), 8.58 (d, *J* = 5.8 Hz, 1H), 8.27 (s, 1H), 8.22 (s, 1H), 8.10 (d, *J* = 8.1 Hz, 1H), 7.93 (d, *J* = 8.3 Hz, 1H), 7.60–7.52 (m, 1H), 7.44 (t, *J* = 8.1 Hz, 1H), 7.14 (d, *J* = 7.6 Hz, 1H), 7.09 (d, *J* = 8.6 Hz, 1H), 6.99 (d, *J* = 7.1 Hz, 1H), 6.54 (t, *J* = 5.8 Hz, 1H), 5.04 (dd, *J* = 12.8, 5.4 Hz, 1H), 3.30 (s, 2H), 2.93–2.80 (m, 1H), 2.68–2.53 (m, 2H), 2.05–1.97 (m, 1H), 1.65–1.36 (m, 8H), 1.26–1.20 (m, 2H). ^13^C NMR (101 MHz, DMSO-*d*_6_) δ 173.28, 170.57, 169.41, 167.76, 166.12, 150.55, 148.19, 147.73, 146.89, 143.78, 142.39, 136.73, 136.15, 136.10, 133.27, 132.66, 130.59, 127.56, 126.26, 124.20, 123.39, 122.79, 122.69, 121.45, 120.70, 119.70, 117.64, 116.84, 110.81, 109.48, 48.99, 42.27, 31.44, 29.50, 29.13, 26.74, 26.56, 22.61. HRMS (ESI) *m*/*z* [M + H]+ calcd for C_38_H_34_ClN_7_O_5_ 704.23882, found 704.23804. UPLC retention time: 5.690 min.

##### 5-((3-Chlorophenyl)amino)-*N*-(5-(((*S*)-1-((2*S*,4*R*)-4-hydroxy-2-(((*S*)-1-(4-(4-methylthiazol-5-yl)phenyl)ethyl)carbamoyl)pyrrolidin-1-yl)-3,3-dimethyl-1-oxobutan-2-yl)amino)-5-oxopentyl)benzo[*c*][2,6]naphthyridine-8-carboxamide (**17**)

General Procedure A (overnight) was followed using **44** (0.036 g, 0.103 mmol) and **57** (0.060 g, 0.103 mmol) to afford the title compound as a yellow solid (0.036 g, 40% yield) after purification by flash column chromatography on SiO_2_ (DCM/MeOH, 93:7 to 9:1). ^1^H NMR (400 MHz, DMSO-*d*_6_) δ 10.20 (s, 1H), 9.66 (s, 1H), 9.01–8.95 (m, 2H), 8.85 (d, *J* = 8.5 Hz, 1H), 8.76 (t, *J* = 5.8 Hz, 1H), 8.59 (d, *J* = 5.9 Hz, 1H), 8.36 (d, *J* = 8.1 Hz, 1H), 8.26 (s, 1H), 8.23 (s, 1H), 8.11 (d, *J* = 8.2 Hz, 1H), 7.94 (d, *J* = 8.5 Hz, 1H), 7.82 (d, *J* = 9.2 Hz, 1H), 7.48–7.40 (m, 3H), 7.37 (d, *J* = 8.3 Hz, 2H), 7.15 (d, *J* = 8.5 Hz, 1H), 5.10 (s, 1H), 4.96–4.85 (m, 1H), 4.52 (d, *J* = 9.3 Hz, 1H), 4.42 (t, *J* = 8.0 Hz, 1H), 4.27 (s, 1H), 3.60 (s, 2H), 3.32–3.27 (m, 2H), 2.45 (s, 3H), 2.35–2.26 (m, 1H), 2.23–2.13 (m, 1H), 2.04–1.96 (m, 1H), 1.83–1.74 (m, 1H), 1.61–1.53 (m, 4H), 1.36 (d, *J* = 7.0 Hz, 3H), 0.93 (s, 9H). ^13^C NMR (101 MHz, DMSO-*d*_6_) δ 172.43, 171.09, 170.06, 166.13, 150.55, 148.22, 148.19, 148.17, 147.74, 145.12, 143.79, 142.40, 136.11, 133.28, 131.58, 130.59, 130.15, 129.28 (2C), 127.57, 126.84 (2C), 126.28, 124.21, 123.40, 122.79, 122.68, 121.47, 120.69, 119.70, 116.84, 69.24, 59.01, 56.85, 56.73, 48.15, 38.17, 35.67 (2C), 35.18, 29.39, 26.93 (3C), 23.64, 22.91, 16.45. HRMS (ESI) *m*/*z* [M + Na]+ calcd for C_47_H_51_ClN_8_O_5_S 897.32893, found 897.32891. UPLC retention time: 5.092 min.

##### 5-((3-Chlorophenyl)amino)-*N*-(7-(((*S*)-1-((2*S*,4R)-4-hydroxy-2-(((*S*)-1-(4-(4-methylthiazol-5-yl)phenyl)ethyl)carbamoyl)pyrrolidin-1-yl)-3,3-dimethyl-1-oxobutan-2-yl)amino)-7-oxoheptyl)benzo[*c*][2,6]naphthyridine-8-carboxamide (**18**)

General Procedure A (overnight) was followed using **44** (0.020 g, 0.057 mmol) and **50** (0.035 g, 0.057 mmol) to afford the title compound as a yellow solid (0.018 g, 35% yield) after purification by flash column chromatography on SiO_2_ (DCM/MeOH, 94:6). ^1^H NMR (400 MHz, DMSO-*d*_6_) δ 10.20 (s, 1H), 9.66 (s, 1H), 9.07–8.94 (m, 2H), 8.85 (d, *J* = 8.6 Hz, 1H), 8.79–8.69 (m, 1H), 8.59 (d, *J* = 5.3 Hz, 1H), 8.37 (d, *J* = 7.8 Hz, 1H), 8.27 (s, 1H), 8.23 (s, 1H), 8.11 (d, *J* = 7.3 Hz, 1H), 7.94 (d, *J* = 8.2 Hz, 1H), 7.79 (d, *J* = 9.2 Hz, 1H), 7.48–7.39 (m, 8.0 Hz, 3H), 7.37 (d, *J* = 8.0 Hz, 2H), 7.14 (d, *J* = 7.6 Hz, 1H), 5.08 (bs, 1H), 4.96–4.87 (m, 1H), 4.52 (d, *J* = 9.2 Hz, 1H), 4.42 (t, *J* = 7.8 Hz, 1H), 4.27 (s, 1H), 3.60 (s, 2H), 3.33–3.26 (m, 2H), 2.44 (s, 3H), 2.29–2.21 (m, 1H), 2.17–2.09 (m, 1H), 2.03–1.96 (m, 1H), 1.82–1.75 (m, 1H), 1.59–1.43 (m, 4H), 1.39–1.20 (m, 7H), 0.93 (s, 9H). ^13^C NMR (101 MHz, DMSO-*d*_6_) δ 172.51, 171.09, 170.08, 166.11, 150.54, 148.21, 148.18 (2C), 147.71, 145.13, 143.78, 142.39, 136.16, 133.28, 131.57, 130.60, 130.14, 129.28 (2C), 127.57, 126.84 (2C), 126.27, 124.21, 123.40, 122.79, 122.68, 121.45, 120.69, 119.70, 116.85, 69.21, 59.00, 56.81, 56.71, 48.15, 38.17, 35.64 (2C), 35.34, 29.53, 28.93, 26.92 (3C), 26.80, 25.90, 22.91, 16.45. HRMS (ESI) *m*/*z* [M + H]+ calcd for C_49_H_55_ClN_8_O_5_S 941.33417, found 941.33469. UPLC retention time: 5.413 min.

##### 5-((3-Chlorophenyl)amino)-*N*-(3-(2-(((*S*)-1-((2*S*,4*R*)-4-hydroxy-2-(((*S*)-1-(4-(4-methylthiazol-5-yl)phenyl)ethyl)carbamoyl)pyrrolidin-1-yl)-3,3-dimethyl-1-oxobutan-2-yl)amino)-2-oxoethoxy)propyl)benzo[*c*][2,6]naphthyridine-8-carboxamide (**19**)

General Procedure A (2 h) was followed using **44** (0.020 g, 0.070 mmol) and **58** (0.042 g, 0.070 mmol) to afford the title compound as a white solid (0.017 g, 27% yield) after purification by flash column chromatography on SiO_2_ (DCM/MeOH, 94:6). ^1^H NMR (400 MHz, DMSO-*d*_6_) δ 10.20 (s, 1H), 9.66 (s, 1H), 9.05–8.94 (m, 2H), 8.90–8.79 (m, 2H), 8.59 (d, *J* = 5.6 Hz, 1H), 8.44 (d, *J* = 7.5 Hz, 1H), 8.27 (s, 1H), 8.24 (s, 1H), 8.12 (d, *J* = 8.1 Hz, 1H), 7.95 (d, *J* = 8.4 Hz, 1H), 7.48–7.28 (m, 6H), 7.14 (d, *J* = 7.6 Hz, 1H), 5.13 (d, *J* = 3.1 Hz, 1H), 4.94–4.85 (m, 1H), 4.55 (d, *J* = 9.6 Hz, 1H), 4.45 (t, *J* = 8.1 Hz, 1H), 4.27 (s, *J* = 15.7 Hz, 1H), 3.97 (s, 2H), 3.66–3.35 (m, 6H), 2.45 (s, 3H), 2.09–1.97 (m, 1H), 1.94–1.83 (m, 2H), 1.82–1.71 (m, 1H), 1.36 (d, *J* = 7.0 Hz, 3H), 0.93 (s, 9H). ^13^C NMR (101 MHz, DMSO-*d*_6_) δ 170.91, 169.50, 168.95, 166.26, 150.55, 148.21, 148.20, 147.75, 145.21, 143.79, 142.40, 136.02, 133.28, 131.57, 130.59, 130.14, 129.30 (2C), 127.56, 126.78 (2C), 126.72, 126.34, 124.22, 123.39, 122.79, 122.67, 121.51, 120.67, 119.68, 116.84, 69.96, 59.01, 57.02, 56.15, 48.23, 38.20, 37.00, 36.31, 29.81, 26.71 (3C), 22.96, 16.45. HRMS (ESI) *m*/*z* [M + H]+ calcd for C_47_H_51_ClN_8_O_6_S 913.32385, found 913.32523. UPLC retention time: 5.260 min.

##### (2*S*,4*R*)-1-((*S*)-3,3-Dimethyl-2-(2-(piperazin-1-yl)acetamido)butanoyl)-4-hydroxy-*N*-((*S*)-1-(4-(4-methylthiazol-5-yl)phenyl)ethyl)pyrrolidine-2-carboxamide Hydrochloride (**59**)

Under a nitrogen atmosphere, a solution of **42** (0.200 g, 0.416 mmol), 2-(1-*tert*-butoxycarbonylpiperazin-4-yl)acetic acid (0.117 g, 0.458 mmol), HATU (0.209 g, 0.520 mmol), and DIPEA (0.3 mL, 1.664 mmol) in dry DMF (2.0 mL) was stirred at room temperature for 1 h. Then, the reaction mixture was poured in ice-water and extracted with EA (×3). The reunited organic phases were washed with water (×2) and brine, dried over Na_2_SO_4_, and evaporated to dryness. The crude residue was purified by flash column chromatography on SiO_2_ (DCM/acetone/MeOH, 63:30:7) to give *tert*-butyl-4-(2-(((*S*)-1-((2*S*,4*R*)-4-hydroxy-2-(((*S*)-1-(4-(4-methylthiazol-5-yl)phenyl)ethyl)carbamoyl)pyrrolidin-1-yl)-3,3-dimethyl-1-oxobutan-2-yl)amino)-2-oxoethyl)piperazine-1-carboxylate (0.137 g, 46% yield) as a colorless oil. ^1^H NMR (400 MHz, CDCl_3_) δ 8.67 (s, 1H), 7.89 (bs, 1H), 7.44 (d, *J* = 7.7 Hz, 1H), 7.40 (d, *J* = 8.1 Hz, 2H), 7.36 (d, *J* = 8.0 Hz, 2H), 5.12–5.02 (m, 1H), 4.76 (t, *J* = 7.9 Hz, 1H), 4.50 (s, 1H), 4.45 (d, *J* = 8.3 Hz, 1H), 4.15 (d, *J* = 11.4 Hz, 1H), 3.68–2.98 (m, 8H), 2.62–2.41 (m, 6H), 2.14–2.02 (m, 1H), 1.66–1.58 (m, 1H), 1.50–1.42 (m, 12H), 1.07 (s, 9H). HRMS (ESI) *m*/*z* [M + H]+ calcd for C_34_H_50_N_6_O_6_S 671.35908, found 671.36080. UPLC retention time: 3.804 min

Then, the solution of the obtained compound (0.130 g, 0.194 mmol) in 4.0N HCl in dioxane (1.3 Ml) was stirred at room temperature for 2 h. The solvent was evaporated to dryness, and the solid was tritured with DEE and collected by filtration, yielding **59** as a white solid (0.115 g, 98% yield). ^1^H NMR (400 MHz, MeOD) δ 10.00–9.85 (m, 1H), 7.57 (d, *J* = 7.7 Hz, 2H), 7.53 (d, *J* = 8.1 Hz, 2H), 5.11–5.00 (m, 1H), 4.67 (s, 1H), 4.63–4.55 (m, 1H), 4.50–4.36 (m, 1H), 4.25–3.99 (m, 2H), 3.92 (d, *J* = 10.9 Hz, 1H), 3.80–3.59 (m, 11H), 2.62 (s, 3H), 2.34–2.19 (m, 1H), 2.01–1.89 (m, 1H), 1.66–1.50 (m, 3H), 1.13–1.03 (m, 9H). HRMS (ESI) *m*/*z* [M + H]+ calcd for C_29_H_42_N_6_O_4_S 571.30665, found 571.30783. UPLC retention time: 3.013 min.

##### (2*S*,4*R*)-1-((*S*)-2-(2-(4-(5-((3-Chlorophenyl)amino)benzo[*c*][2,6]naphthyridine-8-carbonyl)piperazin-1-yl)acetamido)-3,3-dimethylbutanoyl)-4-hydroxy-*N*-((*S*)-1-(4-(4-methylthiazol-5-yl)phenyl)ethyl)pyrrolidine-2-carboxamide (**20**)

General Procedure A (2 h) was followed using **59** (0.067 g, 0.110 mmol) and **44** (0.040 g, 0.110 mmol) to afford the title compound as a yellow solid (0.054 g, 55% yield) after purification by flash column chromatography on SiO_2_ (DCM/MeOH, 95:5). ^1^H NMR (400 MHz, DMSO-*d*_6_) δ 10.18 (s, 1H), 9.69 (s, 1H), 8.98 (s, 2H), 8.89–8.81 (m, 1H), 8.60 (d, *J* = 5.6 Hz, 1H), 8.43 (d, *J* = 7.5 Hz, 1H), 8.31 (s, 1H), 8.12 (d, *J* = 8.1 Hz, 1H), 7.81–7.71 (m, 2H), 7.50 (d, *J* = 8.2 Hz, 1H), 7.48–7.29 (m, 5H), 7.14 (d, *J* = 7.8 Hz, 1H), 5.11 (d, *J* = 2.9 Hz, 1H), 4.96–4.84 (m, 1H), 4.51 (d, *J* = 9.6 Hz, 1H), 4.44 (t, *J* = 8.1 Hz, 1H), 4.28 (s, 1H), 3.85–3.36 (m, 8H), 3.16–2.98 (m, 2H), 2.69–2.53 (m, 2H), 2.46 (s, 3H), 2.10–1.96 (m, 1H), 1.83–1.71 (m, 1H), 1.50–1.33 (m, 3H), 0.94 (s, 9H). ^13^C NMR (101 MHz, DMSO-*d*_6_) δ 170.94, 169.63, 168.98, 168.83, 150.56, 148.22, 148.18, 148.05, 147.58, 145.23, 143.81, 142.35, 137.72, 133.24, 131.58, 130.60, 130.15, 129.30 (2C), 127.60, 126.77 (2C), 125.73, 124.06, 123.12, 123.09, 122.64, 120.55, 120.19, 119.58, 116.81, 69.22, 65.38, 60.87, 58.99, 56.97 (2C), 56.32, 48.22, 38.20, 36.57, 36.17 (2C), 26.77 (3C), 22.97, 16.46. HRMS (ESI) *m*/*z* [M + H]+ calcd for C_48_H_52_ClN_9_O_5_S 902.35789, found 902.35808. UPLC retention time: 4.494 min.

##### *tert*-Butyl (11-(4-(2-Fluoro-5-((4-oxo-3,4-dihydrophthalazin-1-yl)methyl)benzoyl)piperazin-1-yl)-11-oxoundecyl)carbamate (**60**)

Under a nitrogen atmosphere, a solution of **45**(50) (0.267 g, 0.663 mmol), methyl 11-((*tert*-butoxycarbonyl)amino)undecanoate^92^ (0.200 g, 0.663 mmol), HBTU (0.314 g, 0.829 mmol), and Et_3_N (0.185 mL, 0.1.327 mmol) in dry DMF (2.5 mL) was stirred at room temperature for 3 h. The reaction mixture was poured in ice-water and extracted with EA (×3). The reunited organic phases were washed with water (×2) and brine, dried over Na_2_SO_4_, and evaporated to dryness. The crude residue was purified by flash column chromatography on SiO_2_ (DCM/MeOH, 97:3) to give **60** (0.266 g, 62% yield) as a white solid. ^1^H NMR (400 MHz, CDCl_3_) δ 10.66 (s, 1H), 8.47 (d, *J* = 6.2 Hz, 1H), 7.85–7.67 (m, 3H), 7.37–7.29 (m, 2H), 7.04 (t, *J* = 8.6 Hz, 1H), 4.54 (bs, 1H), 4.29 (s, 2H), 3.51 (dd, *J* = 125.0, 74.0 Hz, 8H), 3.09 (t, *J* = 7.0 Hz, 2H), 2.41–2.23 (m, 2H), 1.44 (s, 12H), 1.27 (s, 13H).

##### 2-(2,6-Dioxopiperidin-3-yl)-4-((11-(4-(2-fluoro-5-((4-oxo-3,4-dihydrophthalazin-1-yl)methyl)benzoyl)piperazin-1-yl)-11-oxoundecyl)amino)isoindoline-1,3-dione (**21**)

A solution of **60** (0.260 g, 0.400 mmol) in 4.0N HCl in dioxane was stirred at room temperature for 3 h. The solvent was evaporated to dryness, and the solid was tritured with DEE and collected by filtration, yielding **61** as a white solid (0.218 g, 93% yield).

Under a nitrogen atmosphere, a mixture of **61** (0.117 g, 0.199 mmol), **62**(51) (0.055 g, 0.199 mmol), and DIPEA (87 μL, 0.498 mmol) in dry DMF (1.0 mL) was stirred at 70 °C for 2 h. After cooling to room temperature, the reaction mixture was poured in ice-water, yielding a yellow precipitate, which was collected by filtration and purified by flash column chromatography on SiO_2_ (DCM/acetone, 7:3 to 6:4) followed by preparative TLC purification on SiO_2_ (DCM/MeOH, 97:3) to give **21** as a fluorescent-yellow solid (0.005 g, 3% yield). ^1^H NMR (400 MHz, CDCl_3_) δ 10.30 (s, 1H), 10.12 (s, 1H), 9.02 (s, 1H), 8.63 (s, 1H), 8.46 (s, 1H), 7.87–7.67 (m, 3H), 7.55–7.43 (m, 1H), 7.34–7.30 (m, 1H), 7.09 (d, *J* = 7.1 Hz, 1H), 6.88 (d, *J* = 8.5 Hz, 1H), 6.23 (s, 1H), 4.93 (dd, *J* = 12.0, 5.2 Hz, 1H), 4.28 (s, 2H), 3.86–3.52 (m, 6H), 3.44–3.30 (m, 2H), 3.30–3.21 (m, 2H), 2.94–2.69 (m, 3H), 2.39–2.25 (m, 2H), 2.15–2.07 (m, 1H), 1.71–1.59 (m, 4H), 1.27 (d, *J* = 11.5 Hz, 12H). HRMS (ESI) *m*/*z* [M + H]+ calcd for C_44_H_48_FN_7_O_7_ 806.367752, found 806.36739. UPLC retention time: 5.535 min.

##### Methyl 12-(4-(2-Fluoro-5-((4-oxo-3,4-dihydrophthalazin-1-yl)methyl)benzoyl)piperazin-1-yl)-12-oxododecanoate (**63**)

Under a nitrogen atmosphere, a solution of **45**(50) (0.150 g, 0.372 mmol), 12-methoxy-12-oxododecanoic acid (0.091 g, 0.372 mmol), HBTU (0.176 g, 0.465 mmol), and Et_3_N (0.1 mL, 0.745 mmol) in dry DMF (1.5 mL) was stirred at room temperature for 3 h. The reaction mixture was poured in ice-water and extracted with EA (×3). The reunited organic phases were washed with water (×2) and brine, dried over Na_2_SO_4_, and evaporated to dryness. The crude residue was purified by flash column chromatography on SiO_2_ (DCM/MeOH, 97:3) to give **63** (0.113 g, 51% yield) as a yellow oil, which solidified upon standing. ^1^H NMR (400 MHz, CDCl_3_) δ 10.68 (bs, 1H), 8.52–8.40 (m, 1H), 7.83–7.67 (m, 3H), 7.40–7.28 (m, 2H), 7.04 (t, *J* = 8.8 Hz, 1H), 4.29 (s, 2H), 3.89–3.69 (m, 2H), 3.66 (s, 3H), 3.61–3.22 (m, 6H), 2.39–2.26 (m, 4H), 1.67–1.55 (m, 4H), 1.33–1.21 (m, 12H). HRMS *m*/*z* [M + Na]^+^ calcd for C_33_H_41_FN_4_O_5_ 615.29532, found 615.29651. UPLC retention time: 5.408 min.

##### Methyl 8-(4-(2-Fluoro-5-((4-oxo-3,4-dihydrophthalazin-1-yl)methyl)benzoyl)piperazin-1-yl)-8-oxooctanoate (**64**)

Compound **64** was prepared from **45**(50) (0.300 g, 0.745 mmol) and 8-methoxy-8-oxooctanoic acid (0.140 g, 0.745 mmol) in a similar manner to that described for compound **63** and obtained as a white solid (0.279 g, 70% yield) after purification by flash column chromatography on SiO_2_ (DCM/MeOH, 98:2 to 97:3). ^1^H NMR (400 MHz, CDCl_3_) δ 12.09 (d, *J* = 22.4 Hz, 1H), 8.45 (d, *J* = 5.3 Hz, 1H), 7.82–7.62 (m, 3H), 7.39–7.25 (m, 2H), 7.08–6.93 (m, 1H), 4.28 (s, 2H), 3.89–3.19 (m, 11H), 2.40–2.20 (m, 4H), 1.67–1.52 (m, 4H), 1.36–1.25 (m, 4H). HRMS *m*/*z* [M + Na]^+^ calcd for C_29_H_33_FN_4_O_5_ 559.23327, found 559.23345. UPLC retention time: 4.304 min.

##### 12-(4-(2-Fluoro-5-((4-oxo-3,4-dihydrophthalazin-1-yl)methyl)benzoyl)piperazin-1-yl)-12-oxododecanoic Acid (**65**)

To a stirring solution of **63** (0.097 g, 0.164 mmol) in THF (1.0 mL) at 0 °C was added a solution of LiOH monohydrate (0.069 g, 1.636 mmol) in water (0.5 mL). The reaction mixture was stirred at room temperature for 6 h. The organic solvent was evaporated under reduced pressure, the residue was diluted with water (10 mL), and at 0 °C it was acidified with 2 N HCl (pH = 3) to afford **65** as a white precipitate, collected by filtration and dried (0.084 g, 88% yield). ^1^H NMR (400 MHz, CDCl_3_) δ 11.79 (bs, 1H), 8.44 (d, *J* = 6.8 Hz, 1H), 7.89–7.63 (m, 3H), 7.41–7.28 (m, 2H), 7.03 (t, *J* = 8.5 Hz, 1H), 4.29 (s, 2H), 3.90–3.18 (m, 8H), 2.46–2.18 (m, 4H), 1.67–1.53 (m, 4H), 1.26 (s, 12H). HRMS *m*/*z* [M + H]^+^ calcd for C_32_H_39_FN_4_O_5_ 579.29773, found 579.29912. UPLC retention time: 4.676 min.

##### 8-(4-(2-Fluoro-5-((4-oxo-3,4-dihydrophthalazin-1-yl)methyl)benzoyl)piperazin-1-yl)-8-oxooctanoic Acid (**66**)

Compound **66** was prepared from **64** (0.120 g, 0.224 mmol) in a similar manner to that described for compound **65** and obtained as a white solid (0.076 g, 65% yield). ^1^H NMR (400 MHz, DMSO-*d*_6_) δ 12.61 (s, 1H), 11.99 (bs, 1H), 8.26 (d, *J* = 7.7 Hz, 1H), 7.96 (d, *J* = 7.9 Hz, 1H), 7.90 (t, *J* = 7.5 Hz, 1H), 7.83 (t, *J* = 7.4 Hz, 1H), 7.48–7.41 (m, 1H), 7.39–7.32 (m, 1H), 7.24 (t, *J* = 8.7 Hz, 1H), 4.33 (s, 2H), 3.69–3.45 (m, 4H), 3.22–2.86 (m, 4H), 2.37–2.12 (m, 4H), 1.54–1.38 (m, 4H), 1.31–1.18 (m, 4H).

##### (2*S*,4*R*)-1-((*S*)-2-(12-(4-(2-Fluoro-5-((4-oxo-3,4-dihydrophthalazin-1-yl)methyl)benzoyl)piperazin-1-yl)-12-oxododecanamido)-3,3-dimethylbutanoyl)-4-hydroxy-*N*-((*S*)-1-(4-(4-methylthiazol-5-yl)phenyl)ethyl)pyrrolidine-2-carboxamide (**22**)

General Procedure A (2 h) was followed using **65** (0.050 g, 0.086 mmol) and **42**(52) (0.041 g, 0.086 mmol) to afford the title compound as a white solid (0.034 g, 40% yield) after purification by flash column chromatography on SiO_2_ (DCM/MeOH, 95:5 to 94:6). ^1^H NMR (400 MHz, CDCl_3_) δ 10.75 (d, *J* = 55.3 Hz, 1H), 8.81 (s, 1H), 8.45 (d, *J* = 9.1 Hz, 1H), 7.74 (d, *J* = 29.9 Hz, 3H), 7.60–7.48 (m, 1H), 7.43–7.37 (m, 4H), 7.37–7.28 (m, 2H), 7.08–6.96 (m, 1H), 6.50–6.32 (m, 1H), 5.14–5.04 (m, 1H), 4.75 (t, *J* = 8.0 Hz, 1H), 4.65–4.57 (m, 1H), 4.52 (s, 1H), 4.27 (s, 2H), 4.16 (d, *J* = 10.5 Hz, 1H), 3.77 (s, 2H), 3.61 (d, *J* = 12.1 Hz, 1H), 3.57–3.50 (m, 2H), 3.48–3.19 (m, 4H), 2.59–2.48 (m, 4H), 2.40–2.28 (m, 2H), 2.27–2.19 (m, 2H), 2.13–2.03 (m, 2H), 1.67–1.54 (m, 4H), 1.48 (d, *J* = 6.5 Hz, 3H), 1.39–1.17 (m, 12H), 1.05 (s, 9H). HRMS *m*/*z* [M + Na]^+^ calcd for C_55_H_69_FN_8_O_7_S 1027.48862, found 1027.49135. UPLC retention time: 5.230 min.

##### (2*S*,4*R*)-1-((*S*)-2-(8-(4-(2-Fluoro-5-((4-oxo-3,4-dihydrophthalazin-1-yl)methyl)benzoyl)piperazin-1-yl)-8-oxooctanamido)-3,3-dimethylbutanoyl)-4-hydroxy-*N*-((*S*)-1-(4-(4-methylthiazol-5-yl)phenyl)ethyl)pyrrolidine-2-carboxamide (**23**)

General Procedure A (2 h) was followed using **66** (0.114 g, 0.217 mmol) and **42**(52) (0.105 g, 0.217 mmol) to afford the title compound as a white solid (0.010 g, 5% yield) after purification by flash column chromatography on SiO_2_ (DCM/MeOH, 95:5) followed by preparative TLC purification on SiO_2_ (DCM/MeOH, 9:1). ^1^H NMR (400 MHz, CDCl_3_) δ 10.79 (d, *J* = 64.4 Hz, 1H), 8.72 (s, 1H), 8.46 (d, *J* = 5.7 Hz, 1H), 7.83–7.66 (m, 3H), 7.56–7.47 (m, 1H), 7.36 (d, *J* = 17.2 Hz, 5H), 7.05 (t, *J* = 8.8 Hz, 1H), 6.57–6.42 (m, 1H), 5.14–5.04 (m, 1H), 4.75 (t, *J* = 7.8 Hz, 1H), 4.62 (d, *J* = 8.5 Hz, 1H), 4.51 (s, 1H), 4.27 (s, 2H), 4.15 (d, *J* = 10.9 Hz, 1H), 3.81–3.20 (m, 10H), 2.53 (s, 4H), 2.38–2.21 (m, 4H), 2.13–2.06 (m, 1H), 1.67–1.56 (m, 4H), 1.47 (d, *J* = 6.7 Hz, 3H), 1.35–1.27 (m, 4H), 1.05 (s, 9H). HRMS *m*/*z* [M + H]^+^ calcd for C_51_H_61_FN_8_O_7_S 949.44462, found 949.44427. UPLC retention time: 4.612 min.

##### 4-(4-Fluoro-3-(4-(prop-2-yn-1-yl)piperazine-1-carbonyl)benzyl)phthalazin-1(2*H*)-one (**67**)

To a solution of **45** (0.100 g, 0.248 mmol) in ACN (3.0 mL), K_2_CO_3_ (0.086 g, 0.620 mmol), KI (0.021 g, 0.124 mmol), and propargyl bromide solution (80 wt.% in toluene) (0.031 g, 0.258 mmol) were added, and the mixture was refluxed for 4 h. Then, the solvent was evaporated to dryness and the crude residue was diluted with water, yielding a solid, which was collected by filtration, tritured by DEE, and filtered off to afford the title compound as a yellow-orange solid (0.069 g, 69% yield). ^1^H NMR (400 MHz, DMSO-*d*_6_) δ 12.59 (s, 1H), 8.27 (d, *J* = 7.6 Hz, 1H), 7.98 (d, *J* = 7.8 Hz, 1H), 7.90 (t, *J* = 7.4 Hz, 1H), 7.83 (t, *J* = 7.3 Hz, 1H), 7.46–7.38 (m, 1H), 7.33 (d, *J* = 5.7 Hz, 1H), 7.22 (t, *J* = 8.9 Hz, 1H), 4.33 (s, 2H), 3.63 (s, 2H), 3.31 (s, 2H), 3.19 (s, 3H), 2.48 (s, 1H), 2.34 (s, 2H).

^13^C NMR (101 MHz, DMSO-*d*_6_) δ 164.24, 159.83, 156.76 (d, *J* = 244.4 Hz), 145.36, 135.31 (d, *J* = 3.2 Hz), 133.96, 132.04, 132.00, 129.53, 129.23 (d, *J* = 3.8 Hz), 128.36, 126.55, 125.95, 124.25 (d, *J* = 18.5 Hz), 116.35 (d, *J* = 21.6 Hz), 79.29, 76.54 (2C), 51.43 (d, *J* = 50.4 Hz), 46.57 (d, *J* = 51.4 Hz), 41.58, 36.85. HRMS (ESI) *m*/*z* [M + H]+ calcd for C_23_H_21_FN_4_O_2_ 405.17268, found 405.17332. UPLC retention time: 2.569 min.

##### 4-(4-Fluoro-3-(4-(hex-5-ynoyl)piperazine-1-carbonyl)benzyl)phthalazin-1(2*H*)-one (**68**)

Under a nitrogen atmosphere, a solution of **45** (0.150 g, 0.372 mmol), 5-hexynoic acid (0.042 g, 0.372 mmol), HBTU (0.175 g, 0.465 mmol), and Et_3_N (0.1 mL, 0.744 mmol) in dry DMF (2.0 mL) was stirred at room temperature for 2 h. Then, the reaction mixture was poured in ice-water and extracted with EA (×3). The reunited organic phases were washed with water (×2) and brine, dried over Na_2_SO_4_, and evaporated to dryness. The crude residue was purified by flash column chromatography on SiO_2_ (DCM/MeOH, 96:4) to afford the title compound (0.112 g, 66% yield) as a colorless oil. ^1^H NMR (400 MHz, CDCl_3_) δ 11.70–11.51 (m, 1H), 8.46 (d, *J* = 6.2 Hz, 1H), 7.83–7.65 (m, 3H), 7.40–7.28 (m, 2H), 7.02 (t, *J* = 9.0 Hz, 1H), 4.29 (s, 2H), 3.91–3.66 (m, 3H), 3.67–3.50 (m, 3H), 3.45 (s, 1H), 3.39–3.16 (m, 2H), 2.50 (t, *J* = 7.3 Hz, 2H), 2.33–2.22 (m, 2H), 1.91–1.78 (m, 2H). HRMS (ESI) *m*/*z* [M + H]+ calcd for C_26_H_25_FN_4_O_3_ 461.19889, found 461.19983. UPLC retention time: 3.763 min.

##### (2*S*,4*R*)-1-((*S*)-2-(2-Azidoacetamido)-3,3-dimethylbutanoyl)-4-hydroxy-*N*-((*S*)-1-(4-(4-methylthiazol-5-yl)phenyl)ethyl)pyrrolidine-2-carboxamide (**69**)

Under a nitrogen atmosphere, a solution of **42** (0.150 g, 0.372 mmol), 5-azidopentanoic acid (0.053 g, 0.372 mmol), HOBt (0.011 g, 0.074 mmol), EDC hydrochloride (0.142 g, 0.744 mmol), and NMM (0.40 mL, 3.700 mmol) in dry DMF (2.0 mL) was stirred at room temperature for 5 h. Then, the reaction mixture was poured in ice-water and extracted with EA (×3). The reunited organic phases were washed with water (×2) and brine, dried over Na_2_SO_4_, and evaporated to dryness. The crude residue was purified by flash column chromatography on SiO_2_ (DCM/acetone/MeOH, 75:20:5) to afford the title compound (0.086 g, 44% yield) as a colorless oil. ^1^H NMR (400 MHz, DMSO-*d*_6_) δ 8.99 (s, 1H), 8.38 (d, *J* = 7.7 Hz, 1H), 7.86 (d, *J* = 9.2 Hz, 1H), 7.44 (d, *J* = 8.0 Hz, 2H), 7.38 (d, *J* = 8.1 Hz, 2H), 5.10 (d, *J* = 3.3 Hz, 1H), 4.98–4.86 (m, 1H), 4.53 (d, *J* = 9.2 Hz, 1H), 4.43 (t, *J* = 8.0 Hz, 1H), 4.28 (s, 1H), 3.66–3.55 (m, 2H), 3.38–3.34 (m, 1H), 3.32–3.28 (m, 1H), 2.46 (s, 3H), 2.36–2.24 (m, 1H), 2.23–2.10 (m, 1H), 2.07–1.97 (m, 1H), 1.86–1.75 (m, 1H), 1.61–1.44 (m, 4H), 1.38 (d, *J* = 6.9 Hz, 3H), 0.94 (s, 9H). ^13^C NMR (101 MHz, DMSO-*d*_6_) δ 172.16, 171.08, 170.02, 148.22, 145.13, 131.58, 130.16, 129.29 (2C), 126.85 (2C), 126.73, 69.22, 59.00, 56.84, 56.73, 50.78, 48.15, 38.19, 35.66, 34.67, 28.29, 26.91, 23.06, 22.91, 16.45. HRMS (ESI) *m*/*z* [M + H]+ calcd for C_28_H_39_N_7_O_4_S 570.28625, found 570.28767. UPLC retention time: 4.475 min.

##### (2*S*,4*R*)-1-((*S*)-2-(5-(4-((4-(2-Fluoro-5-((4-oxo-3,4-dihydrophthalazin-1-yl)methyl)benzoyl)piperazin-1-yl)methyl)-1*H*-1,2,3-triazol-1-yl)pentanamido)-3,3-dimethylbutanoyl)-4-hydroxy-*N*-((*S*)-1-(4-(4-methylthiazol-5-yl)phenyl)ethyl)pyrrolidine-2-carboxamide (**24**)

To a solution of **67** (0.041 g, 0.101 mmol) and **69** (0.057 g, 0.101 mmol) in a mixture of DMF/*t*BuOH/H_2_O (1:1:1), CuSO_4_ (0.013 g, 0.050 mmol) and sodium ascorbate (0.059 g, 0.300 mmol) were added, and the reaction mixture was stirred at room temperature for 2 h. Then, the reaction mixture was poured in ice-water, yielding a gray precipitate, which was collected by filtration and purified by flash column chromatography on SiO_2_ (DCM/MeOH, 95:5 to 90:10) to give **24** (0.025 g, 26% yield) as a white solid. ^1^H NMR (400 MHz, DMSO-*d*_6_) δ 12.59 (s, 1H), 8.99 (s, 1H), 8.37 (d, *J* = 7.7 Hz, 1H), 8.27 (d, *J* = 7.7 Hz, 1H), 8.01–7.94 (m, 2H), 7.92–7.78 (m, 3H), 7.48–7.35 (m, 5H), 7.32 (d, *J* = 6.3 Hz, 1H), 7.21 (t, *J* = 9.0 Hz, 1H), 5.10 (d, *J* = 3.2 Hz, 1H), 4.97–4.87 (m, 1H), 4.50 (d, *J* = 9.3 Hz, 1H), 4.42 (t, *J* = 8.1 Hz, 1H), 4.36–4.31 (m, 4H), 4.28 (s, 1H), 3.66–3.54 (m, 4H), 3.19–3.09 (m, 2H), 2.48–2.38 (m, 4H), 2.34–2.25 (m, 2H), 2.21–2.11 (m, 1H), 2.05–1.96 (m, 1H), 1.83–1.74 (m, 2H), 1.50–1.40 (m, 2H), 1.38 (d, *J* = 7.0 Hz, 3H), 0.92 (s, 9H). ^13^C NMR (101 MHz, DMSO-*d*_6_) δ 172.10, 171.07, 170.01, 164.19, 159.84, 156.76 (d, *J* = 244.5 Hz), 148.22, 145.37, 145.12, 143.31, 135.27 (d, *J* = 3.1 Hz), 133.97, 132.04, 131.58, 130.16, 129.52, 129.29 (2C), 129.21, 128.35, 126.84 (2C), 126.70, 126.54, 125.94, 124.27 (d, *J* = 18.5 Hz), 124.21, 116.34 (d, *J* = 21.6 Hz), 69.22, 59.01, 56.85, 56.74, 52.80, 52.30 (2C), 49.40, 48.15, 46.96, 41.72, 38.19, 36.86, 35.63 (2C), 34.55, 29.79, 26.88 (3C), 22.90, 22.79, 16.45. HRMS (ESI) *m*/*z* [M + H]+ calcd for C_51_H_60_FN_11_O_6_S 974.45110, found 974.45129. UPLC retention time: 3.76 min.

##### (2*S*,4*R*)-1-((*S*)-2-(5-(4-(4-(4-(2-Fluoro-5-((4-oxo-3,4-dihydrophthalazin-1-yl)methyl)benzoyl)piperazin-1-yl)-4-oxobutyl)-1*H*-1,2,3-triazol-1-yl)pentanamido)-3,3-dimethylbutanoyl)-4-hydroxy-*N*-((*S*)-1-(4-(4-methylthiazol-5-yl)phenyl)ethyl)pyrrolidine-2-carboxamide (**25**)

Compound **25** was prepared from **68** (0.083 g, 0.180 mmol) and **69** (0.098 g, 0.180 mmol) following the same procedure described for compound **24** and obtained as a crystalline pale-green solid (0.067 g, 36% yield) after purification by flash column chromatography on SiO_2_ (DCM/MeOH, 95:5 to 91:9). ^1^H NMR (400 MHz, DMSO-*d*_6_) δ 12.60 (s, 1H), 9.03 (s, 1H), 8.37 (d, *J* = 7.7 Hz, 1H), 8.27 (d, *J* = 7.7 Hz, 1H), 7.97 (d, *J* = 7.9 Hz, 1H), 7.93–7.77 (m, 4H), 7.49–7.40 (m, 3H), 7.40–7.32 (m, 3H), 7.24 (t, *J* = 9.0 Hz, 1H), 5.10 (d, *J* = 3.3 Hz, 1H), 4.99–4.88 (m, 1H), 4.55–4.46 (m, 1H), 4.42 (t, *J* = 8.1 Hz, 1H), 4.36–4.24 (m, 5H), 3.68–3.56 (m, 4H), 3.55–3.45 (m, 2H), 3.42–3.36 (m, 2H), 3.22–3.10 (m, 2H), 2.71–2.55 (m, 2H), 2.46 (s, 3H), 2.42–2.24 (m, 3H), 2.22–2.14 (m, 1H), 2.04–1.97 (m, 1H), 1.89–1.71 (m, 5H), 1.52–1.40 (m, 2H), 1.37 (d, *J* = 6.9 Hz, 3H), 0.93 (s, 9H). HRMS (ESI) *m*/*z* [M + H]+ calcd for C_54_H_64_FN_11_O_7_S 1030.47732, found 1030.47773. UPLC retention time: 4.292 min.

##### *tert*-Butyl 2-(2,3-Difluoro-6-(2-morpholinothiazol-4-yl)phenoxy)acetate (**70**)

*tert*-Butyl bromoacetate (0.434 mL, 0.575 g, 2.95 mmol) was added to a stirred suspension of **46**(53) (0.800 g, 2.68 mmol) and K_2_CO_3_ (0.927 g, 6.71 mmol). The suspension was stirred for 18 h at rt and filtered, and the filtrate was evaporated to dryness. The residue was purified by flash column chromatography on SiO_2_ (PE/EA, 95:5 to 90:10) to afford **70** (0.800 g, 73% yield) as a light-yellow solid. ^1^H NMR (400 MHz, CDCl_3_) δ 7.92 (ddd, *J* = 2.3, 6.2, 8.8 Hz, 1H), 7.68 (s, 1H), 7.10–6.87 (m, 1H), 4.67 (d, *J* = 1.7 Hz, 2H), 4.00–3.79 (m, 4H), 3.68–3.47 (m, 4H), 1.53 (s, 9H). ^13^C NMR (101 MHz, CDCl_3_) δ 169.77, 167.40, 150.32 (dd, *J* = 11.8, 249.8 Hz), 145.30, 144.64–144.23 (m), 143.98 (dd, *J* = 14.4, 246.1 Hz), 124.35, 124.04 (dd, *J* = 3.9, 7.8 Hz), 111.42 (d, *J* = 17.0 Hz), 107.71, 82.55, 70.00 (d, *J* = 7.5 Hz), 66.22 (2C), 48.61 (2C), 28.09 (3C).

##### 2-(2,3-Difluoro-6-(2-morpholinothiazol-4-yl)phenoxy)acetic Acid (**71**)

A solution of 4.0N HCl in dioxane (15 mL) was added to **70** (0.780 g, 1.89 mmol), and the resulting suspension was stirred at room temperature for 16 h. The solvent was evaporated to dryness, and the residue was tritured with DEE and collected by filtration to afford **71** (0.672 g, 91% yield) as a light-yellow solid. ^1^H NMR (400 MHz, CD_3_OD) δ 7.48 (ddd, *J* = 2.3, 5.7, 8.1 Hz, 1H), 7.27 (s, 1H), 7.18 (td, *J* = 7.5, 9.2 Hz, 1H), 5.01 (d, *J* = 1.5 Hz, 2H), 4.02–3.86 (m, 4H), 3.86–3.67 (m, 4H). ^13^C NMR (101 MHz, CD_3_OD) δ 171.72, 169.44, 152.57 (dd, *J* = 11.6, 252.2 Hz), 144.55 (d, *J* = 1.7 Hz), 143.30 (dd, *J* = 15.0, 248.0 Hz), 136.41 (d, *J* = 13.3 Hz), 124.82 (dd, *J* = 3.9, 8.6 Hz), 118.47, 111.66 (d, *J* = 18.2 Hz), 106.15, 69.24 (d, *J* = 9.0 Hz), 65.05 (2C), 49.17 (2C).

##### 2-(2,3-Difluoro-6-(2-morpholinothiazol-4-yl)phenoxy)-*N*-(8-(2-((2-(2,6-dioxopiperidin-3-yl)-1,3-dioxoisoindolin-4-yl)oxy)acetamido)octyl)acetamide (**26**)

General Procedure A (16 h) was followed using **71** (0.032 g, 0.091 mmol) and **53** (0.045 g, 0.091 mmol) to afford the title compound as a yellow solid (0.012 g, 17% yield) following purification by flash column chromatography on SiO_2_ (DCM/acetone, 75:25). ^1^H NMR (400 MHz, CDCl_3_) δ 8.66 (bs, 1H), 7.76 (t, *J* = 7.8 Hz, 1H), 7.66–7.53 (m, 2H), 7.49–7.39 (m, 1H), 7.21 (d, *J* = 8.4 Hz, 1H), 7.08–6.90 (m, 3H), 4.98 (dd, *J* = 12.0, 5.0 Hz, 1H), 4.65 (s, 2H), 4.60 (s, 2H), 3.94–3.78 (m, 4H), 3.60–3.47 (m, 4H), 3.40–3.25 (m, 4H), 3.02–2.73 (m, 3H), 2.26–2.12 (m, 1H), 1.56–1.47 (m, 2H), 1.45–1.28 (m, 10H). ^13^C NMR (101 MHz, CDCl_3_) δ 170.91, 170.82, 168.05, 167.97, 166.64, 166.57, 165.99, 154.51, 150.69 (dd, *J* = 251.69, 11.5 Hz), 146.51–146.44 (m), 144.19 (dd, *J* = 47.3, 14.0 Hz), 144.63 (d, *J* = 10.6 Hz), 137.04, 133.60, 125.25 (d, *J* = 3.5 Hz), 124.47 (dd, *J* = 7.6, 4.3 Hz), 119.47, 118.14, 117.38, 112.41 (d, *J* = 17.1 Hz), 106.11, 72.41 (d, *J* = 5.1 Hz), 68.01, 66.13 (2C), 49.34, 48.55 (2C), 39.16, 39.10, 31.49, 29.44, 29.23, 29.13, 29.06, 26.70, 26.68, 22.59. HRMS (ESI) *m*/*z* [M + H]+ calcd for C_38_H_42_F_2_N_6_O_9_S 797.27748, found 797.27834. UPLC retention time: 5.208 min.

##### 2-(2,3-Difluoro-6-(2-morpholinothiazol-4-yl)phenoxy)-*N*-(2-(2-(2-(2-((2-(2,6-dioxopiperidin-3-yl)-1,3-dioxoisoindolin-4-yl)oxy)acetamido)ethoxy)ethoxy)ethyl)acetamide (**27**)

General Procedure A (16 h) was followed using **71** (0.025 g, 0.070 mmol) and **72** to afford the title compound as a light-yellow solid (0.009 g, 17% yield) after purification by flash column chromatography on SiO_2_ (DCM/MeOH, 97:3). ^1^H NMR (400 MHz, CDCl_3_) δ 8.75 (bs, 1H), 7.75 (t, *J* = 7.8 Hz, 1H), 7.68–7.52 (m, 3H), 7.40 (bs, 1H), 7.18 (d, *J* = 8.3 Hz, 1H), 7.07–6.91 (m, 2H), 4.96 (dd, *J* = 11.7, 5.1 Hz, 1H), 4.63 (s, 2H), 4.58 (s, 2H), 3.94–3.80 (m, 4H), 3.79–3.63 (m, 8H), 3.60–3.43 (m, 8H), 2.97–2.64 (m, 3H), 2.22–2.10 (m, 1H). HRMS (ESI) *m*/*z* [M + H]+ calcd for C_36_H_38_F_2_N_6_O_11_S 801.23601, found 801.23745. UPLC retention time: 4.333 min.

##### 2-(2,3-Difluoro-6-(2-morpholinothiazol-4-yl)phenoxy)-*N*-(1-((2-(2,6-dioxopiperidin-3-yl)-1,3-dioxoisoindolin-4-yl)oxy)-2-oxo-6,9,12-trioxa-3-azatetradecan-14-yl)acetamide (**28**)

General Procedure A (16 h) was followed using **71** (0.033 g, 0.092 mmol) and **55** to afford the title compound as a light-yellow solid (0.012 g, 16% yield) after purification by flash column chromatography on SiO_2_ (DCM/MeOH, 97:3). ^1^H NMR (400 MHz, CDCl_3_) δ 8.96 (bs, 1H), 7.75 (t, *J* = 7.9 Hz, 1H), 7.70–7.59 (m, 2H), 7.55 (d, *J* = 7.3 Hz, 1H), 7.46 (bs, 1H), 7.18 (d, *J* = 8.4 Hz, 1H), 7.08 (s, 1H), 6.98 (dd, *J* = 16.7, 9.0 Hz, 1H), 4.92 (dd, *J* = 12.1, 5.3 Hz, 1H), 4.64 (s, 2H), 4.56 (s, 2H), 3.90–3.82 (m, 4H), 3.71–3.47 (m, 20H), 2.92–2.61 (m, 3H), 2.19–2.07 (m, 1H). HRMS (ESI) *m*/*z* [M + H]+ calcd for C_38_H_42_F_2_N_6_O_12_S 845.26222, found 845.26303. UPLC retention time: 4.395 min.

##### 2-(2,3-Difluoro-6-(2-morpholinothiazol-4-yl)phenoxy)-*N*-(6-((2-(2,6-dioxopiperidin-3-yl)-1,3-dioxoisoindolin-4-yl)amino)hexyl)acetamide (**29**)

General Procedure A (16 h) was followed using **71** (0.048 g, 0.122 mmol) and **56** (0.050 g, 0.122 mmol) to afford the title compound as a yellow solid (0.015 g, 18% yield) after purification by flash column chromatography on SiO_2_ (DCM/acetone/MeOH, 90:10:0 to 89:10:1). ^1^H NMR (400 MHz, CDCl_3_) δ 8.09 (s, 1H), 7.63–7.56 (m, 1H), 7.56–7.48 (m, 1H), 7.11 (d, *J* = 7.0 Hz, 1H), 7.05 (s, 1H), 7.04–6.96 (m, 1H), 6.93 (s, 1H), 6.90 (d, *J* = 8.5 Hz, 1H), 6.25 (s, 1H), 4.93 (dd, *J* = 5.3, 11.9 Hz, 1H), 4.60 (s, 2H), 3.89–3.78 (m, 4H), 3.64–3.51 (m, 4H), 3.36 (q, *J* = 6.8 Hz, 2H), 3.33–3.26 (m, 2H), 2.97–2.68 (m, 3H), 2.18–2.11 (m, 1H), 1.79–1.35 (m, 8H). ^13^C NMR (101 MHz, CDCl_3_) δ 170.87, 170.74, 169.50, 168.25, 167.92, 167.59, 150.76 (dd, *J* = 11.6, 251.4 Hz), 146.96, 146.37–146.09 (m), 144.62 (dd, *J* = 1.5, 9.1 Hz), 144.15 (dd, *J* = 14.0, 247.3 Hz), 136.15, 132.48, 125.04, 124.53 (dd, *J* = 4.0, 7.8 Hz), 116.63, 112.43 (d, *J* = 17.2 Hz), 111.46, 109.90, 105.96, 72.42 (d, *J* = 4.9 Hz), 66.11 (2C), 48.87, 48.62 (2C), 42.52, 38.95, 31.41, 29.42, 29.13, 26.62, 26.55, 22.82. HRMS (ESI) *m*/*z* [M + H]+ calcd for C_34_H_36_F_2_N_6_O_7_S 711.2407, found 711.2412. UPLC retention time: 5.564 min.

##### 2-(2,3-Difluoro-6-(2-morpholinothiazol-4-yl)phenoxy)-*N*-(8-((2-(2,6-dioxopiperidin-3-yl)-1,3-dioxoisoindolin-4-yl)amino)octyl)acetamide (**30**)

General Procedure A (16 h) was followed using **71** (0.020 g, 0.056 mmol) and **47** (0.024 g, 0.056 mmol) to afford the title compound as a yellow solid (0.016 g, 50% yield) after purification by flash column chromatography on SiO_2_ (DCM/MeOH, 99:1 to 96:4) followed by further HPLC purification (Agilent Technologies 1200; column, Eclipse XDB-C18 4.6 mm × 150 mm (5 μm); flow rate, 1.0 mL/min; DAD 190-650 nm; isocratic eluent, ACN/H_2_O 70:30). ^1^H NMR (400 MHz, CDCl_3_) δ 8.14 (s, 1H), 7.64–7.55 (m, 1H), 7.55–7.45 (m, 1H), 7.11 (d, *J* = 7.1 Hz, 1H), 7.04 (s, 1H), 7.04–6.97 (m, 1H), 6.93 (s, 1H), 6.90 (d, *J* = 8.6 Hz, 1H), 6.25 (s, 1H), 5.02–4.86 (m, 1H), 4.60 (s, 2H), 3.97–3.76 (m, 4H), 3.64–3.48 (m, 4H), 3.34 (q, *J* = 6.8 Hz, 2H), 3.28 (q, *J* = 6.6 Hz, 2H), 2.99–2.67 (m, 3H), 2.21–2.08 (m, 1H), 1.81–1.31 (m, 12H). ^13^C NMR (101 MHz, CDCl_3_) δ 170.90, 170.71, 169.50, 168.28, 167.88, 167.61, 150.78 (dd, *J* = 10.5, 249.5 Hz), 147.01, 146.45–145.86 (m), 144.63 (d, *J* = 10.5 Hz), 144.14 (dd, *J* = 14.0, 247.4 Hz), 136.12, 132.49, 124.99, 124.52 (dd, *J* = 3.8, 7.8 Hz), 116.65, 112.41 (d, *J* = 17.2 Hz), 111.39, 109.86, 105.96, 72.41 (d, *J* = 5.1 Hz), 66.11 (2C), 48.86, 48.62 (2C), 42.59, 39.10, 31.42, 29.45, 29.14, 29.12, 29.11, 26.78, 26.72, 22.84. HRMS (ESI) *m*/*z* [M + H]+ calcd for C_36_H_40_F_2_N_6_O_7_S 739.27200, found 739.27369. UPLC retention time: 6.040 min.

##### 2-(2,3-Difluoro-6-(2-morpholinothiazol-4-yl)phenoxy)-*N*-(8-((2-(2,6-dioxopiperidin-3-yl)-1,3-dioxoisoindolin-4-yl)amino)octyl)acetamide (**31**)

General Procedure A (16 h) was followed using **71** (0.018 g, 0.049 mmol) and **48**(90) (0.022 g, 0.049 mmol) to afford the title compound as a yellow solid (4.5 mg, 12% yield) after purification by flash column chromatography on SiO_2_ (DCM/MeOH, 97:3) followed by HPLC purification (Agilent Tecnologies 1200; column, Eclipse XDB-C18 4.6 mm × 150 mm (5 μm); flow rate, 0.8 mL/min; DAD 190-650 nm; isocratic eluent, ACN/H_2_O 70:30). ^1^H NMR (400 MHz, CDCl_3_) δ 8.44 (bs, 1H), 7.64 (t, *J* = 6.5 Hz, 1H), 7.46 (t, *J* = 7.8 Hz, 1H), 7.36 (bs, 1H), 7.09 (d, *J* = 7.1 Hz, 1H), 7.05–6.95 (m, 2H), 6.83 (d, *J* = 8.5 Hz, 1H), 6.51 (bs, 1H), 4.98–4.85 (m, 1H), 4.56 (s, 2H), 3.93–3.80 (m, 4H), 3.77–3.64 (m, 8H), 3.62–3.51 (m, 6H), 3.45–3.34 (m, 2H), 2.96–2.66 (m, 3H), 2.20–2.05 (m, 1H). HRMS (ESI) *m*/*z* [M + H]+ calcd for C_34_H_36_F_2_N_6_O_9_S 743.23053, found 743.23191. UPLC retention time: 4.945 min.

##### *tert*-Butyl (2-(2-(2-(2,3-difluoro-6-(2-morpholinothiazol-4-yl)phenoxy)ethoxy)ethoxy)ethyl)carbamate (**73**)

DIAD (0.087 mL, 0.442 mmol) was slowly added to a stirred ice-cooled solution of **46**(53) (0.120 g, 0.402 mmol), *tert*-butyl (2-(2-(2-hydroxyethoxy)ethoxy)ethyl)carbamate (0.110 g, 0.442 mmol), and PPh_3_ (0.116 g, 0.442 mmol) in dry THF (5.0 mL). The solution was stirred at 0 °C for 30 min and then at room temperature for 16 h. The reaction mixture was quenched with water and extracted with EA (×3). The reunited organic phases were dried over anhydrous Na_2_SO_4_ and concentrated to dryness, affording a crude residue, which was purified by flash column chromatography on SiO_2_ (DCM/EA, 9:1 to 8:2) to give **73** (0.100 g, 70% yield) as a yellow oil. ^1^H NMR (400 MHz, DMSO-*d*_6_) δ 7.89 (ddd, *J* = 8.8, 6.3, 2.3 Hz, 1H), 7.58 (s, 1H), 7.00–6.88 (m, 1H), 5.10–4.94 (m, 1H), 4.35–4.24 (m, 2H), 3.92–3.79 (m, 6H), 3.72–3.61 (m, 4H), 3.60–3.48 (m, 6H), 3.39–3.25 (m, 2H), 1.43 (s, 9H). ^13^C NMR (101 MHz, CDCl_3_) δ 169.71, 155.99, 150.35 (dd, *J* = 11.6, 249.5 Hz), 145.52, 144.65 (dd, *J* = 13.6, 246.2 Hz), 125.04 (d, *J* = 2.1 Hz), 123.84 (dd, *J* = 4.0, 7.8 Hz), 111.42 (d, *J* = 17.0 Hz), 107.47, 79.17, 72.56 (d, *J* = 6.0 Hz), 70.46, 70.34, 70.30 (2C), 66.22 (2C), 48.60 (2C), 40.35, 28.39 (3C).

##### 2-(2-(2-(2,3-Difluoro-6-(2-morpholinothiazol-4-yl)phenoxy)ethoxy)ethoxy)ethan-1-amine hydrochloride (**74**)

A solution of 4.0N HCl in dioxane (2.0 mL) was added to **73** (0.100 g, 0.189 mmol), and the resulting suspension was stirred at room temperature overnight. The solvent was evaporated to dryness, and the residue was tritured with DEE and collected by filtration to afford **74** (0.080 g, 91% yield) as a white solid. ^1^H NMR (400 MHz, MeOD) δ 7.49–7.40 (m, 1H), 7.28 (s, 1H), 7.04 (dd, *J* = 16.9, 9.0 Hz, 1H), 4.25 (s, 1H), 3.86–3.50 (m, 18H), 3.02 (s, 2H).

##### 4-((2-(2-(2-(2,3-Difluoro-6-(2-morpholinothiazol-4-yl)phenoxy)ethoxy)ethoxy)ethyl)amino)-2-(2,6-dioxopiperidin-3-yl)isoindoline-1,3-dione (**32**)

General Procedure A (6 h) was followed using **74** (0.044 g, 0.095 mmol) and **62**(51) (0.024 g, 0.086 mmol), to afford the title compound as a yellow solid (0.06 g, 54% yield) following purification by flash column chromatography on SiO_2_ (DCM/acetone, 9:1). ^1^H NMR (400 MHz, CDCl_3_) δ 8.21 (s, 1H), 7.89 (ddd, *J* = 8.7, 6.3, 2.1 Hz, 1H), 7.58 (s, 1H), 7.52–7.44 (m, 1H), 7.11 (d, *J* = 7.1 Hz, 1H), 6.99–6.88 (m, 2H), 6.51 (t, *J* = 5.3 Hz, 1H), 4.90 (dd, *J* = 12.1, 5.3 Hz, 1H), 4.40–4.24 (m, 2H), 3.98–3.81 (m, 6H), 3.79–3.62 (m, 6H), 3.59–3.38 (m, 6H), 2.96–2.65 (m, 3H), 2.19–2.03 (m, 1H). HRMS (ESI) *m*/*z* [M + H]+ calcd for C_32_H_33_F_2_N_5_O_8_S 686.20907, found 686.20855. UPLC retention time: 5.749 min.

### Metabolic Stability in Cryopreserved Human Hepatocytes

Cryopreserved human hepatocytes (pooled suspension hepatocytes, Gibco) were thawed placing in a 37 °C shaking water bath according to the manufacturer’s specifications and resuspended in Williams E medium (WEM) to have 1 × 10^6^ cells/mL. Samples with the test compound at 1 μM were incubated at 37 °C, and aliquots of 50 μL were collected at 0, 10, 30, 60, 120, and 240 min. The incubations were quenched 1:3 with ice-cold acetonitrile (containing 1 μM labetalol as the internal standard). Samples were then centrifuged at 12000 rpm for 5 min at 4 °C. The supernatant was concentrated by evaporation under a nitrogen stream and reconstituted with DMSO. Blank was prepared similarly but in the absence of the investigated compounds.

### Metabolic Stability in CYP3A4 and Human Liver Cytosol

Metabolism of the selected compounds was evaluated upon incubation with human liver cytosol (Gibco) and supersome recombinant CYP isoforms 3A4 expressed in baculovirus-infected insect cells (Gibco). For metabolism in CYP3A4, test compounds (1 μM) were incubated in 0.1 M potassium phosphate buffer (pH 7.4) and allowed to equilibrate at 37 °C. Freshly prepared 25 mM nicotinamide adenine dinucleotide phosphate (NADPH, 1 mM final concentration) was added to the incubation mixture to start the reaction. For metabolism in human liver cytosol, reactions were initiated by adding test compounds (1 μM) to the mix reaction constituted by the human liver cytosol (1 mg/mL) prewarmed in potassium phosphate buffer (pH 7.4). Similarly, in the inhibition experiments of the AOX1, test compounds were incubated in the presence or absence of the selective inhibitor hydralazine (50 μM). In both procedures, aliquots (50 μL) of the incubation mix were removed at 0, 10, 20, 30, and 60 min (CYP3A4) or 0, 30, 60, and 90 min (human liver cytosol) and added to 50 μL of ice-cold acetonitrile quench solution (internal standard, 1 μM labetalol) to stop the reaction. Samples were then centrifuged at 12 000 rpm for 5 min at 4 °C. The supernatant was concentrated by evaporation under a nitrogen stream and reconstituted in DMSO. The blank was prepared similarly but in the absence of the investigated compounds. Samples were then analyzed by LC–MS/MS (see the Analytical procedure).

### Stability of Compound **1** (**dBet1**) in Four Different Solutions during LC–MS Acquisitions

**dBet1 (1)** (10 mM in DMSO) was diluted 1:1000 in PBS/ACN (50:50, vol/vol) to afford a final concentration of 10 μM; 100 μL was drawn and dried under a gentle nitrogen stream at 40 °C for 30 min. After that evaporation was completed, the dry residue was reconstituted with 100 μL of DMSO. Additional three solutions of **dBet1** (1) (10 μM) were prepared in PBS, PBS/ACN (50:50, vol/vol), and DMSO. The four solutions were stored into the autosampler of the LC apparatus at 37 °C for 12 h. Injections (2 μL) were programmed every 3 h. At each time point (0, 3, 6, and 12 h), the samples underwent analyses with a Thermo Q-exactive mass spectrometer (Thermo Fisher Scientific, Waltham, MA) as described in the dedicated section.

### UHPLC-MS Analysis

A Thermo Q-exactive mass spectrometer (Thermo Fisher Scientific, Waltham, MA) was used. The LC system, governed by Chromeleon X-press software, consists of a Binary pump, a thermostated autosampler, and a column compartment, all Dionex Ultimate 3000 series modules (Thermo Fisher Scientific, Waltham, MA). A volume of 2 μL was injected for each sample. Chromatographic separation of analytes was conducted in reverse-phase chromatography. In brief, a Luna Omega 1.6 μm Polar (C18, 2.1 mm × 150 mm) was used, and the mobile phases consisted of water (A) and acetonitrile (B), both containing formic acid at 0.1%. The LC flow was set at 0.400 mL/min in a 12 min gradient elution as follows: 99.5:0.5 (A/B) to 5:95 (A/B) over 10 min, 5:95 (A/B) for 2 min, and then reversion back to 99.5:0.5 (A/B) over 2.5 min. The column was operating at a constant temperature of 40 °C. The LC effluents were introduced into the Q-Exactive mass spectrometer by an H-ESI source that operated in the positive mode with a sheath gas flow rate of 45; an auxiliary gas flow rate of 15; a spray voltage of 3.5 kV; capillary temperature and auxiliary gas heater temperature, respectively, of 320 and 350 °C; and S-lens RF level 50. The Q-Exactive mass spectrometer operates in the data-dependent scan (DDS) mode, with a resolution of 70.000 in full mass and 17.500 in MS/MS, in the scan mass range of 100–1500 at collision energies of 15, 60, and 120 V. The MS/MS data were processed using “MetaSite 5.1.8 Mass 3.3.5” and “WebMetabase release-4.0.4” (MolecularDiscovery, Ltd.).

## Supporting Information Available

PROTACs’ chemical structures and half-life values (t1/2) upon incubation in cryopreserved human hepatocytes (Table S1); structure and half-life values (t1/2) of the constituent ligands upon incubation in cryopreserved human hepatocytes (Table S2); metabolism identification data upon incubation in cryopreserved human hepatocytes (Table S3); metabolic profile for compound **7** (MZ1) (Figure S1); kinetics of the metabolism of compounds **33** and **34** in human liver cytosol and their chromatograms, respectively (Figures S2 and S3) (PDF)
Molecular formula strings (CSV)

## Abbreviations Used

ACN: acetonitrile
ADME: absorption, distribution, metabolism, and excretion
AR: androgen receptor
BET: bromodomain and extraterminal
Boc: *tert*-butoxycarbonyl
BRD4: bromodomain-containing protein 4
cIAP: cell inhibitor of apoptosis protein
CK2: casein kinase 2
CRBN: cereblon
CYP3A4: human cytochrome P450 3A4
Da: Dalton unified atomic mass unit
DAD: diode-array detection
DCM: dichloromethane
DEE: diethyl ether
DIAD: diisopropyl azodicarboxylate
DIPEA: *N,N*-diisopropylethylamine
DMF: dimethylformamide
DMSO: dimethyl sulfoxide
DNA: deoxyribonucleic acid
EA: ethyl acetate
EDC: *N*-(3-dimethylaminopropyl)-*N*′-ethylcarbodiimide
ESI: electrospray ionization
Et_3_N: triethylamine
h: hour
*h*AOX: human aldehyde oxidase
HATU: 1-[bis(dimethylamino)methylene]-1*H*-1,2,3-triazolo[4,5-*b*]pyridinium 3-oxid hexafluorophosphate
H-ESI: heated electrospray ionization
HRMS: high-resolution mass spectroscopy
Hz: Hertz
LC–MS: liquid chromatography–mass spectrometry
LC–MS/MS: liquid chromatography with tandem mass spectrometry
logP: negative logarithm of the partition coefficient
MDM2: mouse double minute 2 homolog
MeOH: methanol
HBTU: *N,N,N*′*,N*′*-*tetramethyl-*O*-(1*H*-benzotriazol-1-yl)uronium hexafluorophosphate
MoCo: molybdenum pyranopterin cofactor
MS/MS: tandem mass spectrometry
NMM: *N*-methyl morpholine
PARP: poly(ADP-ribose) polymerase
PBS: phosphate-buffered saline
PE: petroleum ether
PEG: poly(ethylene glycol)
POI: protein of interest
PPh_3_: triphenylphosphine
PROTACs: proteolysis targeting chimeras
Q-TOF LC–MS: quadrupole time-of-flight liquid chromatography–mass spectrometry
RF: radio frequency
rt: room temperature
t1/2: half life
THF: tetrahydrofuran
TLC: thin-layer chromatography
TMS: tetramethylsilane
UPLC-MS: ultraperformance liquid chromatography-tandem mass spectrometry
VHL: von Hippel-Lindau
WEM: Williams E medium
