# Supplementary material for: Understanding the Metabolism of Proteolysis Targeting Chimeras (PROTACs): The Next Step toward Pharmaceutical Applications
Source: J Med Chem. 2020 Oct 7;63(20):11615–38. doi: 10.1021/acs.jmedchem.0c00793 (PMC8015227; doi:10.1021/acs.jmedchem.0c00793)
Supplement: Supplementary file 1 — jm0c00793_si_001.pdf [file jm0c00793_si_001.pdf]

Supporting Information

Understanding the metabolism of Proteolysis Targeting Chimeras (PROTACs): the next step toward pharmaceutical applications

Laura Goracci,[a‡] Jenny Desantis,[a‡] Aurora Valeri,[b] Beatrice Castellani,[a] Michela Eleuteri,[c] Gabriele Cruciani[a]*

[a]*Department of Chemistry, Biology and Biotechnology, University of Perugia, 06123 Perugia, Italy;*

[b]*Molecular Horizon, srl, Bettona 06084, Italy.*

[c]*Montelino Therapeutics, LLC,.7 Powdermill Lane, Southborough, MA 01772.*

Table of Contents

Table S1. PROTACs t1/2 values upon human hepatocytes incubation. S2-S4

Table S2. Free ligands t1/2 values upon human hepatocytes incubation. S5

Table S3. Metabolism identification data. S6-S12

Figure S1. Metabolic profile for compound **7** (**MZ1**). S13

Figure S2. Kinetics of the metabolism of compounds **33** and **34** in human liver cytosol. S14

Figure S3. Chromatogram and MS/MS spectra of compounds **33** and **34** after incubation in human liver cytosol in the absence and in the presence of hydralazine. S15

Table S1. PROTACs' chemical structures and half-life values (t1/2) upon incubation in cryopreserved human hepatocytes.

Structure	Entry	R		t1/2 (min)
		Linker	E3 ligase ligand	
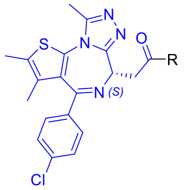	**1 (dBet1)**	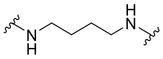	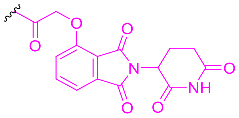	135
	**2 (dBet6)**	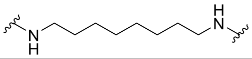	"	18.2
	**3 (ARV-825)**	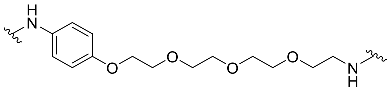	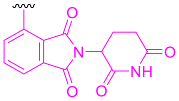	27.7
	**4**	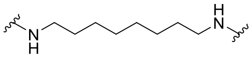	"	55.1
	**5**	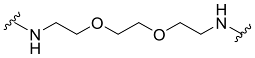	"	49.4
	**6**	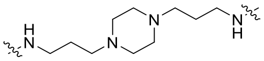	"	87.0
	**7 (MZ1)**	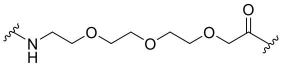	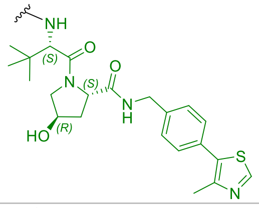	210
	**8**	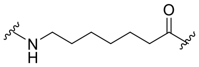	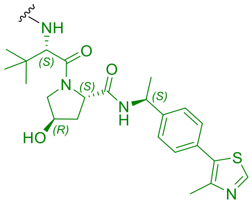	81.8
	**9**	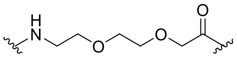	"	137
	**10 (ARV-771)**	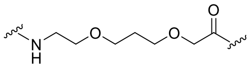	"	151
	**11**	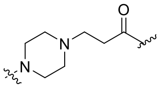	"	>>240
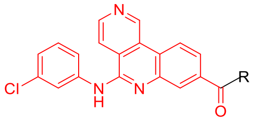	**12**	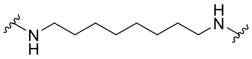	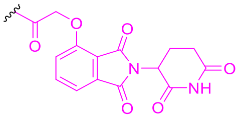	41.4
	**13**	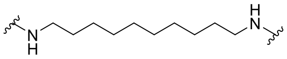	"	33.6
	**14**	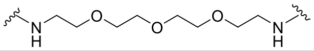	"	76.8
	**15**	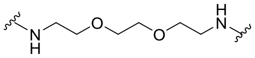	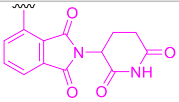	26.8
	**16**	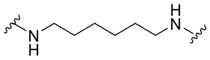	"	34.7

	**17**	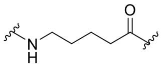	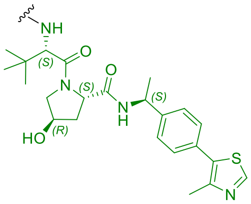	204
	**18**	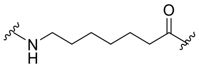	“	209
	**19**	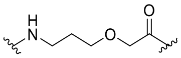	“	159
	**20**	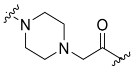	“	218
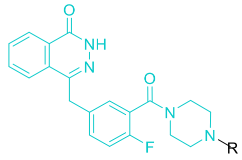	**21**	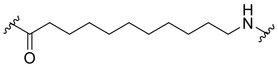	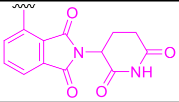	15.7
	**22**	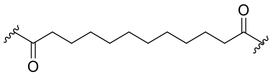	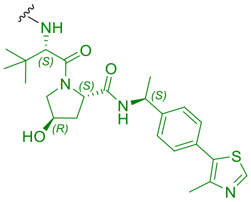	95
	**23**	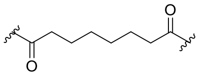	“	91.2
	**24**	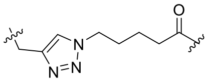	“	>>240
	**25**	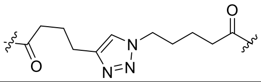	“	>>240
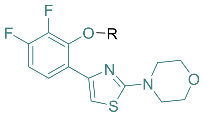	**26**	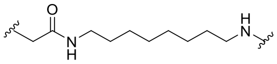	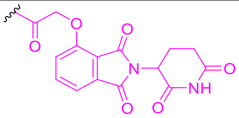	8.4
	**27**	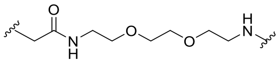	“	39.4
	**28**	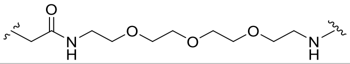	“	66.4
	**29**	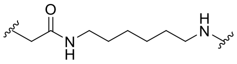	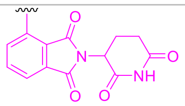	53.5
	**30**	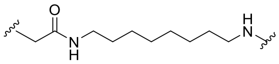	“	47.6
	**31**	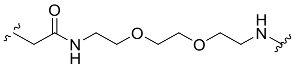	“	38.4
	**32**	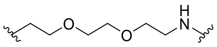	“	45.3

	**33**	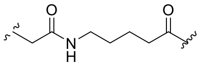	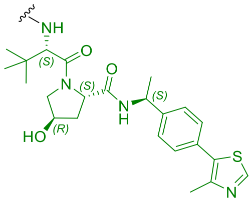	74.3
	**34**	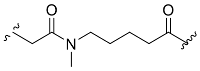	“	86.1
	**35**	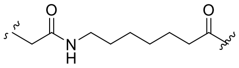	“	25
	**36**	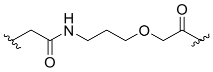	“	25.2
	**37**	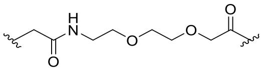	“	37.6
	**38**	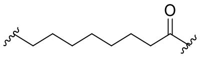	“	67.6
	**39**	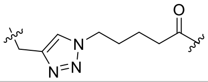	“	40.1
	**40**	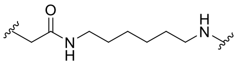	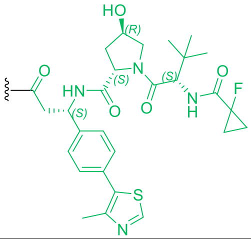	66.7

Table S2. Structure and half-life values (t1/2) of the free ligands upon incubation in cryopreserved human hepatocytes.

Entry	Structure	t1/2 (min)
**41 (pomalidomide)**	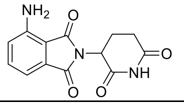	140
**42 (VHL ligand)**	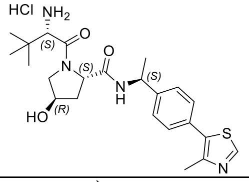	92
**43 ((+)-JQ1)**	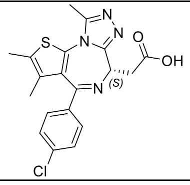	>>240
**44 (CX4945)**	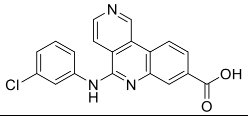	99.7
**45 (Olaparib-analogue)**	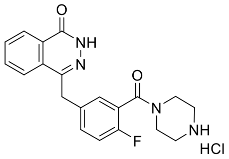	>>240
**46 (AR ligand)**	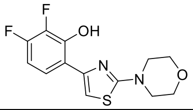	18.3

Table S3. Metabolism upon incubation in cryopreserved human hepatocytes, including the parent compounds (cp), their metabolites (Met ID), the underlying biotransformation, retention time (RT), chemical formula, accurate mass of the precursor [M+H]+ and the most informative MS/MS fragments (a-e).

cp	Met. ID	Biotransformation	RT (min)	Formula	M+H+	a	b	c	d	e
**1**			7.11							
	M+16	Aliphatic Hydroxylation	6.17	[C38H37ClN8O8S+H]+	801.2225	399.0687	287.0668	357.0575	176.0345	_
	M+18	Amide Hydrolysis	6.36	[C38H39ClN8O8S+H]+	803.2354	675.1774	383.0720	341.0622	218.0741	_
**2**			7.93							
	M-509	*N*-Dealkylation	4.15	[C15H13N3O6+H]+	332.0884	287.0671	75.0240			
	M-441	*N*-Dealkylation	6.59	[C19H18ClN5OS+H]+	400.0995	383.0732	341.0628	_	_	_
	M+18	Amide Hydrolysis	7.06	[C42H47ClN8O8S+H]+	859.3002	731.2382	383.0732	246.0705	_	_
	M+18	Amide Hydrolysis	7.04	[C42H47ClN8O8S+2H]++	430.1536	731.2446	366.1247	383.0717	527.2327	129.0661
	M-2	Dehydrogenation	7.42	[C42H43ClN8O7S+H]+	839.2748	383.0735	341.0631	_	_	_
	M+16	Aliphatic Hydroxylation	7.41	[C42H45ClN8O8S+H]+	857.2845	399.0685	357.0581	839.2729	_	_
**3**			8.48							
	M-561	*O*-Dealkylation	5.06	[C17H19N3O6+H]+	362.1341	300.0979	251.1026	189.0657	_	_
	M-547	*O*-Dealkylation	5.12	[C17H17N3O7+H]+	376.1139	330.1084	300.0985	265.0819	_	_
	M-517	*O*-Dealkylation	5.19	[C19H23N3O7+H]+	406.1602	300.0979	344.1238	388.1503	362.1347	_
	M-503	*O*-Dealkylation	5.24	[C19H21N3O8+H]+	420.1392	374.1347	300.0974	362.1334	_	_
	M-459	*O*-Dealkylation	5.39	[C21H25N3O9+H]+	464.1665	418.1609	300.0978	189.0661	_	_
	M-522	Amide Hydrolysis	6.96	[C19H17ClN4O2S+H]+	401.0827	383.0728	357.0935	_	_	_
	M-373	*O*-Dealkylation	7.28	[C27H24ClN5O4S+H]+	550.1308	383.0728	341.0624	355.0781	152.0706	_
	M-431	*O*-Dealkylation	7.32	[C25H22ClN5O2S+H]+	492.1258	383.0728	341.0622	152.0706	_	_
	M-387	*O*-Dealkylation	7.33	[C27H26ClN5O3S+H]+	536.1526	518.1412	383.0728	341.0628	_	_
	M-329	*O*-Dealkylation	7.37	[C29H28ClN5O5S+H]+	594.1584	341.0622	383.0735	152.0628	_	_
	M-285	*O*-Dealkylation	7.43	[C31H32ClN5O6S+H]+	638.1843	341.063	383.0715	_	_	_
	M-241	*N*-Dealkylation	7.47	[C33H36ClN5O7S+H]+	682.2109	341.063	383.0715	_	_	_
**4**			9.00							
	M-383	*N*-Dealkylation	6.58	[C19H18ClN5OS+H]+	400.0985	383.0725	341.0622	289.0554	75.0237	_
	M-241	*N*-Dealkylation	7.49	[C27H32ClN5O3S+H]+	542.1999	524.1907	341.0627	218.0753	383.0739	_
	M+16	Aliphatic Hydroxylation	7.76	[C40H43ClN8O6S+H]+	799.2796	341.0632	383.0736	314.0528	84.0818	_
	M-2	Dehydrogenation	8.30	[C40H41ClN8O5S+H]+	781.2682	341.0628	86.0972	383.0727	60.0818	_
**5**			7.71							
	M-425	*O*-Dealkylation	5.03	[C17H19N3O6+H]+	362.1340	300.0976	251.1018	172.0391	189.0659	_
	M-411	*O*-Dealkylation+Ox	5.12	[C17H17N3O7+H]+	376.1134	330.1082	358.1037	265.0820	300.0978	172.0392
	M-343	*O*-Dealkylation	6.39	[C21H22ClN5O2S+H]+	444.1254	426.1151	341.0622	246.0138	210.0378	_
	M-329	*O*-Dealkylation+Ox	6.42	[C21H20ClN5O3S+H]+	458.1043	383.0725	289.0561	_	_	_
	M-285	*O*-Dealkylation+Ox	6.46	[C23H24ClN5O4S+H]+	502.1303	383.0722	341.0609	75.0236	_	_
	M-241	*N*-Dealkylation+Ox	6.54	[C25H28ClN5O5S+H]+	546.1579	341.0627	314.0518	272.0291	246.0694	_
	M+16	Aliphatic Hydroxylation	6.72	[C38H39ClN8O8S+H]+	803.2354	357.0567	399.0675	172.0392	330.0463	_
**6**			5.96							
	M-493	*N*-Dealkylation+Ox	5.08	[C16H15N3O6+H]+	346.1033	175.0502	_	_	_	_
	M-241	*N*-Dealkylation+Ox	5.16	[C29H36ClN7O3S+2H]++	299.6217	383.0720	269.6112	139.1228	341.0622	199.1441
	M+16	Aliphatic Hydroxylation	5.17	[C42H47ClN10O6S+2H]++	428.1618	419.1539	399.0684	305.0492	291.6243	277.6086
	M+18	Amide Hydrolysis	5.51	[C42H49ClN10O6S+2H]++	429.1696	440.1306	420.1643	383.0728	283.6268	127.1230
	M+16	Aliphatic Hydroxylation	5.61	[C42H47ClN10O6S+2H]++	428.1618	457.2558	542.2099	399.0677	291.6243	277.6086
	M-367	*N*-Dealkylation+Ox	6.40	[C22H22ClN5O3S+H]+	472.1184	383.0728	341.0622	314.0513	289.0542	218.0746
	M-439	*N*-Dealkylation	6.42	[C19H18ClN5OS+H]+	400.0992	383.0724	355.0782	289.0553	_	_
**7**			7.74							
	M-513	*O*-Dealkylation	5.45	[C24H32N4O5S+H]+	489.2157	318.1267	188.0527	86.0606	_	_
	M-455	*O*-Dealkylation	5.55	[C26H34N4O7S+H]+	547.2211	318.1264	188.0526	86.0605	205.0793	_
	M-411	*O*-Dealkylation	5.60	[C28H38N4O8S+H]+	591.2471	318.1266	188.0527	86.0606	103.0392	_
	M-425	*O*-Dealkylation	5.65	[C28H40N4O7S+H]+	577.2681	260.1488	318.1267	188.0526	86.0606	_

	M-558	*O*-Dealkylation	6.38	[C21H22ClN5O2S+H]+	444.1247	426.1146	383.0721	341.0622	_	_
	M-544	*O*-Dealkylation-Ox	6.41	[C21H20ClN5O3S+H]+	458.1040	383.0723	341.0622	412.0993	218.0746	75.0236
	M-470	*O*-Dealkylation	6.52	[C25H30ClN5O4S+H]+	532.1771	341.0611	383.0720	218.0744	289.0555	_
	M-456	*O*-Dealkylation	6.55	[C25H28ClN5O5S+H]+	546.1563	341.0618	383.0729	218.0744	75.0235	_
	M+16	Aliphatic Hydroxylation	6.82	[C49H60ClN9O9S2+2H]+2	509.6889	318.1269	128.0621	357.0568	234.0691	75.0237
	M-2	Dehydrogenation	7.43	[C49H59ClN9O8S+H]+2	500.6834	318.1267	188.0528	683.2411	618.2902	_
**8**			8.26							
	M-554	*N*-Dealkylation	6.58	[C19H18ClN5OS+H]+	400.0988	383.0728	75.0236	_	_	_
	M+14	Aliphatic Carbonylation	6.95	[C49H60ClN9O6S2]+2	484.6885	399.0678	357.0568	202.0683	332.1422	115.0545
	M+16	Aliphatic Hydroxylation	7.33	[C49H60ClN9O6S2+H2]+2	485.6982	639.2521	202.0688	332.1429	234.0700	_
	M+16	Aliphatic Hydroxylation	7.68	[C49H60ClN9O6S2+H2]+2	485.6979	639.2530	202.0690	332.1426	526.1680	102.0922
	M+16	Aliphatic Hydroxylation	7.74	[C49H60ClN9O6S2+H2]+2	485.6972	639.2546	611.2582	202.0695	526.1682	_
	M-2	Dehydrogenation	7.84	[C49H59ClN9O5S2+H]+2	476.6925	570.3131	202.0695	621.2436	332.1442	_
**9**			8.11							
	M-640	Amide Hydrolysis	4.80	[C17H21N3O2S+H]+	332.1426	202.0686	91.0549	147.0266	_	_
	M-469	*O*-Dealkylation	5.72	[C25H34N4O5S+H]+	503.2332	332.1433	202.0690	175.0581	147.0266	_
	M-425	*O*-Dealkylation+Ox	5.78	[C27H38N4O6S+H]+	547.2592	332.1432	202.0688	175.0579	188.1281	216.1231
	M-411	*O*-Dealkylation	5.81	[C27H36N4O7S+H]+	561.2386	202.0687	332.1431	175.0582	147.0272	_
	M-486	*O*-Dealkylation	6.15	[C23H24ClN5O3S+H]+	486.1367	400.1007	383.0737	341.0639	_	_
	M-471	*O*-Dealkylation	6.24	[C25H32N4O5S+H]+	501.2172	330.1274	202.0687	175.0578	115.0546	_
	M-528	*O*-Dealkylation+Ox	6.40	[C21H22ClN5O2S+H]+	444.1258	426.1153	383.0729	246.0141	218.0747	_
	M-514	*O*-Dealkylation	6.41	[C21H20ClN5O3S+H]+	458.1053	383.0725	412.0988	246.0143	218.0747	_
	M-484	*O*-Dealkylation+Ox	6.46	[C23H26ClN5O3S+H]+	488.1521	383.0737	341.0629	246.0143	218.0751	_
	M-572	*N*-Dealkylation	6.58	[C19H18ClN5OS+H]+	400.0995	383.0735	75.0237	_	_	_
	M+14	Aliphatic Carbonylation	6.81	[C48H58ClN9O8S2]+2	493.6788	202.0689	655.2108	399.0675	357.0560	_
	M+16	Aliphatic Hydroxylation	7.16	[C48H58ClN9O8S2+H2]+2	494.6844	202.0687	657.2262	629.2318	219.0949	332.1430
	M+14	Aliphatic Carbonylation	7.57	[C48H58ClN9O8S2]+2	493.6770	202.0684	332.14301	115.0545	_	_
	M-2	Dehydrogenation	7.69	[C48H57ClN9O7S2+H2]+2	485.6794	202.0688	639.2160	332.1432	769.2915	526.1322
**10**			8.09							
	M-654	Amide Hydrolysis	4.81	[C17H21N3O2S+H]+	332.1427	219.0950	131.0863	115.0546	_	_
	M-483	*O*-Dealkylation	5.73	[C25H34N4O5S+H]+	503.2315	202.0683	141.0698	86.0964	_	_
	M-411	*O*-Dealkylation	5.90	[C28H38N4O7S+H]+	575.2525	332.1427	216.1230	244.1179	_	_
	M-425	*O*-Dealkylation	5.92	[C28H40N4O6S+H]+	561.2745	332.1427	202.0686	230.1387	_	_
	M-486	*O*-Dealkylation	6.23	[C24H26ClN5O3S+H]+	500.1250	400.0093	341.0622	_	_	_
	M-542	*O*-Dealkylation	6.39	[C21H22ClN5O2S+H]+	444.1261	426.1156	102.0471	383.0728	_	_
	M-528	*O*-Dealkylation	6.39	[C21H20ClN5O3S+H]+	458.1039	412.0992	383.0720	300.0337	_	_
	M-586	*N*-Dealkylation	6.58	[C19H18ClN5OS+H]+	400.0998	383.0733	341.0622	_	_	_
	M-470	*O*-Dealkylation	6.62	[C24H26ClN5O4S+H]+	516.1458	383.0729	341.0612	300.0342	_	_
	M-426	Amide Hydrolysis	6.72	[C26H30ClN5O5S+H]+	560.1735	341.0630	426.1150	218.0746	_	_
	M+16	Aliphatic Hydroxylation	7.15	[C49H60ClN9O8S2+2H]2+	501.6932	202.0685	671.2435	399.0689	332.1438	_
	M+16	Aliphatic Hydroxylation	7.59	[C49H60ClN9O8S2+2H]2+	501.6919	202.0682	671.2400	357.0569	332.1414	_
	M+16	Aliphatic Hydroxylation	7.75	[C49H60ClN9O8S2+2H]2+	501.6917	400.0990	653.2299	202.0683	332.1421	_
**11**			6.39							
	M-498	*N*-Dealkylation	5.73	[C23H25ClN6OS+2H]++	235.0825	97.0106	_	_	_	_
	M+16	Aliphatic Hydroxylation	5.77	[C49H59ClN10O6S2+2H]++	492.1949	782.3201	652.2467	497.1521	202.0685	332.1427
	M+16	Aliphatic Hydroxylation	6.09	[C49H59ClN10O6S2+2H]++	492.1949	766.3260	481.1572	383.0721	115.0543	218.0634
**12**			8.34							
	M-458	N-Dealkylation	4.19	[C15H13N3O6+H]+	332.0871	287.0661	75.0237	_	_	_
	M+16	Aliphatic Hydroxylation	7.14	[C42H40ClN7O8+H]+	806.2688	332.0582	287.0658	303.0563	_	_
	M-440	Amide Hydrolysis	7.51	[C19H12ClN3O2+H]+	350.0697	_	_	_	_	_
**13**			8.97							
	M-486	*N*-Dealkylation	4.17	[C15H13N3O6+H]+	332.0871	287.0659	75.0236	_	_	_

	M-469	*N*-Dealkylation	6.75	[C19H13ClN4O+H]+	349.0848	268.0863	_	_	_	_
	M-468	Amide Hydrolysis	7.51	[C19H12ClN3O2+H]+	350.0698	269.0940	223.0510	_	_	_
**14**			7.02							
	M-462	*O*-Dealkylation	4.10	[C17H17N3O7+H]+	376.1137	358.1033	287.0669	133.0287	_	_
	M-374	*O*-Dealkylation	4.42	[C21H25N3O9+H]+	464.1665	358.1034	287.0662	133.0284	_	_
	M-448	*O*-Dealkylation+Ox	4.15	[C17H15N3O8+H]+	390.0927	315.0608	287.0670	176.0344	_	_
	M-404	*O*-Dealkylation+Ox	4.30	[C19H19N3O9+H]+	434.1190	388.1141	315.0618	358.1028	287.0657	176.0237
	M-360	*O*-Dealkylation+Ox	4.46	[C21H23N3O10+H]+	478.1454	358.1035	287.0665	176.0346	_	_
	M-445	*O*-Dealkylation	6.44	[C21H17ClN4O2+H]+	393.1110	269.0943	304.0633	332.0588	_	_
	M-431	*O*-Dealkylation+Ox	6.50	[C21H15ClN4O3+H]+	407.0898	304.0630	269.0944	332.0578	_	_
	M-401	*O*-Dealkylation	6.52	[C23H21ClN4O3+H]+	437.1376	375.1016	269.0948	332.0589	304.0636	_
	M-387	*O*-Dealkylation+Ox	6.58	[C23H19ClN4O4+H]+	451.1165	269.0949	304.0640	332.0582	_	_
	M-343	*O*-Dealkylation+Ox	6.61	[C25H23ClN4O5+H]+	495.1421	304.0634	269.0941	332.0579	375.0996	_
	M+18	Amide Hydrolysis	6.34	[C42H42ClN7O11+H]+	856.2697	728.2129	375.1009	269.0945	332.0586	304.0634
**15**			7.62							
	M-418	*O*-Dealkylation	4.85	[C15H15N3O5+H]+	318.1080	300.0981	207.0764	189.0661	84.0450	_
	M-374	*O*-Dealkylation	5.03	[C17H19N3O6+H]+	362.1339	300.0975	251.1024	172.0391	362.1338	189.0658
	M-360	*O*-Dealkylation+Ox	5.12	[C17H17N3O7+H]+	376.1142	300.0981	330.1094	265.0822	172.0391	189.0650
	M-343	*O*-Dealkylation	6.44	[C21H17ClN4O2+H]+	393.1104	269.0941	304.0628	_	_	_
	M-329	*O*-Dealkylation+Ox	6.5	[C21H15ClN4O3+H]+	407.0910	304.0643	269.0949	_	_	_
	M-285	*O*-Dealkylation+Ox	6.57	[C23H19ClN4O4+H]+	451.1157	269.0941	304.0630	332.0576	_	_
	M-241	*N*-Dealkylation+Ox	6.61	[C25H23ClN4O5+H]+	495.1422	304.0633	269.0943	332.0583	375.1009	_
	M-386	Amide Hydrolysis	7.51	[C19H12ClN3O2+H]+	350.0694	_	_	_	_	_
**16**			8.86							
	M-241	*N*-Dealkylation	7.29	[C25H23ClN4O3+H]+	463.1536	269.0951	304.0641	332.0591	179.0607	_
	M-354	Amide Hydrolysis	7.50	[C19H12ClN3O2+H]+	350.0700	179.0602	_	_	_	_
**17**			8.02							
	M+30	Aliphatic Hydroxylation/ Alcoholic Oxidation	7.31	[C47H49ClN8O7S+H2]+2	453.1642	202.0686	141.0701	268.0869	_	_
	M+16	Aliphatic Hydroxylation	7.40	[C47H51ClN8O6S+H2]+2	446.1750	332.1427	304.0636	202.0685	115.0546	_
	M+16	Aromatic Hydroxylation	7.58	[C47H51ClN8O6S+H2]+2	446.1741	532.2098	202.0684	332.1427	447.1202	_
	M-525	Amide Hydrolysis	7.51	[C19H12ClN3O2+H]+	_	_	_	_	_	_
**18**			8.52							
	M-554	*N*-Dealkylation	6.76	[C19H13ClN4O+H]+	303.278	_	_	_	_	_
	M+30	Aliphatic Hydroxylation/ Alcoholic Oxidation	7.73	[C49H53ClN8O7S+H2]+2	467.1802	202.0689	304.0637	269.0954	_	_
	M-2	Dehydrogenatio n	7.96	[C49H54ClN8O5S+H2]+2	451.1849	202.0686	332.1425	570.2274	542.2335	_
	M+16	Aromatic Hydroxylation	7.98	[C49H55ClN8O6S+H2]+2	460.1912	332.1433	202.0689	560.2423	588.2366	475.1549
**19**			8.17							
	M-559	Amide Hydrolysis	4.81	[C17H21N3O2S+H]+	332.1423	302.1425	91.0548	147.0260	_	_
	M-388	*O*-Dealkylation	5.72	[C25H34N4O5S+H]+	503.2314	332.1423	202.0683	160.0341	175.0575	_
	M-470	*O*-Dealkylation+Ox	6.60	[C22H17ClN4O3+H]+	421.1064	269.0954	332.0581	304.0659	_	_
	M-484	*O*-Dealkylation	6.66	[C22H19ClN4O2+H]+	407.1271	269.0949	304.0645	332.0579	_	_
	M+30	Aliphatic Hydroxylation/ Alcoholic Oxidation	7.41	[C47H49ClN8O8S+H2]+2	461.1627	_	_	_	_	_
	M+16	Aliphatic Hydroxylation	7.55	[C47H51ClN8O7S+H2]+2	454.1724	576.2029	202.0691	332.1443	175.0580	160.0342
	M+16	Aromatic Hydroxylation	7.70	[C47H51ClN8O7S+H2]+2	454.1720	576.2018	548.2061	202.0685	320.0585	405.1110
**20**			6.63							

	M+16	Aliphatic Hydroxylation	5.81	[C48H52ClN9O6S+2H]++	459.6795	430.1429	332.1427	304.0636	202.0685	160.0341
	M+16	Aliphatic Hydroxylation	6.02	[C48H52CLN9O6S+2H]++	459.6797	587.2168	446.1378	332.1427	304.0636	202.0685
	M+30	Aliphatic Hydroxylation/ Alcoholic Oxidation	6.13	[C48H50ClN9O7S+2H]++	466.6695	430.1429	304.0636	269.0947	99.0917	91.0542
	M+16	Aromatic Hydroxylation	6.24	[C48H52ClN9O6S+2H]++	459.6798	587.2168	446.1378	320.0585	285.0897	202.0685
	M+16	Aromatic Hydroxylation	6.33	[C48H52ClN9O6S+2H]++	459.6802	332.1427	446.1378	320.0585	332.1427	202.0682
	M-552	Amide Hydrolysis+Ox	7.40	[C19H12ClN3O2+H]+	350.0690	268.0869	179.0604	_	_	_
**21**			8.46							
	M-439	Amide Hydrolysis	3.85	[C20H19FN4O2+H]+	367.1554	281.0711	208.0545	_	_	_
	M-241	*N*-Dealkylation+Ox	6.86	[C31H37FN4O5+H]+	565.2821	547.2725	281.0722	208.0558	253.0772	_
**22**			8.06							
	M+32	*N*-Dealkylation	6.52	[C48H57ClN9O7S2+H2]+2	519.2519	332.1429	202.0686	281.0723	706.3598	_
	M+16	Aliphatic Hydroxylation	7.04	[C55H69FN8O8S+H2]+2	511.2539	332.1425	202.0684	281.0719	115.0544	86.0607
	M+16	Aliphatic Hydroxylation	7.08	[C55H69FN8O8S+H2]+2	511.2544	332.1432	202.0687	281.0724	175.0576	445.2272
	M+16	Aliphatic Hydroxylation	7.20	[C55H69FN8O8S+H2]+2	511.2545	332.1424	202.0683	281.0719	367.1559	147.0262
	M+16	Aliphatic Hydroxylation	7.40	[C55H69FN8O8S+H2]+2	511.2549	348.1384	281.0728	141.0720	261.0661	_
	M-2	Dehydrogenatio n	7.76	[C55H68FN8O7S+H2]+2	502.2491	559.2720	332.1428	202.0687	281.0725	253.0773
**23**			7.10							
	M+16	Aliphatic Hydroxylation	6.56	[C51H61FN8O8S+H2]+2	483.2236	332.1429	202.0689	281.0719	503.2087	_
	M+16	Aliphatic Hydroxylation	6.71	[C51H61FN8O8S+H2]+2	483.2236	634.3036	332.1427	_	_	_
	M-2	Dehydrogenatio n	6.90	[C51H61FN8O7S+H]+2	474.2183	202.0689	503.2097	616.2950	332.1429	_
**24**			5.57							
	M-607	*N*-Dealkylation	3.73	[C20H19FN4O2+H]+	367.1566	281.0721	208.0557	107.0292	_	_
	M+16	Aliphatic Hydroxylation	5.28	[C51H60FN11O7S+H]+	990.4461	643.3151	530.2310	115.0542	218.0628	281.0721
	M+32	*N*-Dealkylation+Ox	5.40	[C51H60FN11O8S+2H]++	503.7235	805.3792	399.1463	332.1427	202.0685	160.0341
	M+16	Aliphatic Hydroxylation	5.64	[C51H60FN11O7S+2H]++	495.7253	789.3842	383.1514	332.1427	281.0721	202.0685
	M-334	*N*-Dealkylation+Ox	5.74	[C31H41N7O6S+H]+	640.2908	332.1427	202.0685	187.0450	175.0576	309.1557
	M-415	*N*-Dealkylation+Ox	5.78	[C28H38N4O6S+H]+	559.2583	332.1427	147.0263	115.0542	202.0685	160.0341
	M-2	Dehydrogenatio n	6.62	[C51H59N11O6S+H]+	486.7212	332.1427	613.3045	641.2995	202.0685	253.0772
**25**			6.48							
	M-526	*N*-Dealkylation	5.03	[C26H26FN7O3+H]+	504.2156	367.1565	281.0721	261.0659	253.0750	_
	M-698	Amide Hydrolysis	5.78	[C17H21N3O2S+H]+	332.1429	202.0685	160.0341	147.0263	115.0542	_
	M+32	*N*-Dealkylation+Ox	5.93	[C54H64FN11O9S+2H]++	531.7374	332.1427	202.0685	175.0576	219.0950	_
	M+16	Aliphatic Hydroxylation	6.11	[C54H64FN11O8S+H]+	1046.472 0	168.1383	333.1921	218.0634	196.1332	543.2636
	M+16	Aliphatic Hydroxylation	6.23	[C54H64FN11O8S+2H]++	523.7397	715.3362	697.3257	367.1565	332.1424	202.0685
**26**	-		8.11							
	M-338	Amide hydrolysis	4.50	[C23H30N4O6+H]+	459.2238	287.0672	176.0343	_	_	_
	M-323	*N*-Dealkylation+Ox	5.80	[C23H27N3O8+H]+	474.1871	287.0658	176.0343	456.1765	_	_
	M-440	Amide hydrolysis+Ox	6.65	[C15H14F2N2O4S+H]+	357.0715	298.0580	154.0225	143.0306	139.0014	_
	M+32	*O*-Dealkylation+Ox (Morph)	6.97	[C38H42F2N2O11S+H]+	829.2673	313.0461	287.0672	267.0410	253.0250	_
**27**			6.88							
	M-383	*O*-Dealkylation	4.43	[C19H19N3O8+H]+	418.1245	_	_	_	_	_
	M-403	*O*-Dealkylation	5.54	[C17H17F2N3O4S+H]+	398.0981	267.0390	_	_	_	_
	M+32	*O*-Dealkylation	5.56	[C36H38F2N6O+H]+	833.2258	414.0940	371.0507	358.1031	287.0660	267.0397

	M-359	*O*-Dealkylation+Ox	5.62	[C19H21F23O5S+H]+	442.1243	267.0404	184.9869	154.0227	139.0015	_
	M-401	*O*-Dealkylation	6.19	[C17H19F2N3O4S+H]+	400.1137	382.1026	139.0012	154.0223	199.0025	_
	M-357	*O*-Dealkylation	6.28	[C19H23F2N3O5S+H]+	444.1400	299.0674	241.0221	199.0026	184.9874	139.0013
	M-343	*O*-Dealkylation+Ox	6.31	[C19H21F2N3O6S+H]+	458.1198	241.0227	139.0016	199.0029	130.0326	267.0398
	M+16	Aliphatic Hydroxylation	6.33	[C36H38F2N6O12S+H+]+	817.2305 and 799.2196	380.0875	358.1034	309.0508	296.0435	176.0344
	M-444	Amide Hydrolysis	6.65	[C15H14F2N2O4S+H]+	357.0716	298.0584	154.0227	139.0015	_	_
**28**			6.97							
	M-469	*O*-Dealkylation	4.13	[C17H17N3O7+H]+	376.1139	287.0657	176.0345	_	_	_
	M-471	*O*-Dealkylation	4.27	[C17H15N3O7+H]+	374.0983	_	_	_	_	_
	M-359	*O*-Dealkylation	4.47	[C21H25F2N3O6S+H]+	486.1505	_	_	_	_	_
	M-447	*O*-Dealkylation	5.54	[C17H17F2N3O4S+H]+	398.0981	154.0228	_	_	_	_
	M+32	*O*-Dealkylation	5.62	[C38H42F2N6O14S+H]+	877.2521	414.0930	371.0490	358.1025	343.0558	287.0665
	M-359	*O*-Dealkylation	5.71	[C21H25F2N3O6S+H]+	486.1505	267.0393	184.9863	_	_	_
	M+18	Amide hydrolysis	6.20	[C38H44F2N6O13S+ H]+	863.2728	735.2151	488.1682	382.1034	358.1040	339.0616
	M-401	*O*-Dealkylation	6.27	[C19H23F2N3O5S+H]+	444.1399	241.0253	184.9868	199.0035	139.0016	_
	M-387	*O*-Dealkylation	6.29	[C19H21F2N3O6S+H]+	458.1192	241.0243	139.0018	_	_	_
	M-431	*O*-Dealkylation	6.31	[C17H17F2N3O5S+H]+	253.0232	199.0023	139.0015	127.0357	_	_
	M-343	*O*-Dealkylation	6.36	[C21H25F2N3O7S+H]+	502.1454	299.0665	253.0248	241.0244	199.0023	139.0012
	M-488	Amide hydrolysis	6.68	[C15H14F2N2O4S+H]+	357.0715	169.0338	75.0050	298.0581	154.0227	139.0016
**29**	-	-	5.88							
	M-338	Amide hydrolysis	4.67	[C19H24N4O4+H]+	373.1872	_	_	_	_	_
	M-354	Amide hydrolysis	6.67	[C15H14F2O4S+H]+	357.0715	298.0581	187.0026	154.0226	139.0230	_
	M-241	Aliphatic Hydroxylation *N*-Dealkylation	7.10	[C21H25F2N3O5S+H]+	470.1556	299.0675	241.0217	199.0030	139.0018	_
	M+176	Glucoronidation	5.15	[C40H45F2N6O13S+2H]++	444.1400	241.0246	169.0333	139.0015	127.0353	_
	M+32	*O*-Dealkylation	6.98	[C34H36F2N6O9S+H]+	743.2305	343.0565	267.0401	241.0248	199.0027	127.0357
	M-323	*N*-Dealkylation	6.25	[C19H21N3O6+H]+	370.1396	274.0818	_	_	_	_
	M+18	Amide hydrolysis	7.70	[C34H38F2N6O8S+H]+	729.2513	311.0662	299.0635	241.0236	130.0321	86.0070
**30**			9.18							
	M-383	*N*-Dealkylation	6.48	[C15H15F2N3O3S+H]+	356.0875	75.0238	298.0578	154.0229	139.0014	127.0354
	M-382	Amide Hydrolysis+Ox	6.56	[C15H14F2N2O4S+H]+	357.0715	75.005	298.0587	187.0022	154.0226	169.0338
	M-323	*N*-Dealkylation+Ox	6.96	[C21H25N3O6+H]+	416.1816	398.1716	201.0664	175.0504	84.0452	_
	M+16	Aliphatic Hydroxylation	7.81	[C36H40F2N6O8S+H]+	755.2669	311.0649	240.0288	199.0029	175.0501	154.0230
	M+32	*O*-Dealkylation+Ox (Morph)	7.90	[C36H40F2N6O9S+H]+	771.2618	313.0453	343.0572	267.0407	253.0249	199.0033
**31**	-		7.79							
	M-338	Amide hydrolysis	4.19	[C19H24N4O6+H]+	405.1769	369.1341	_	_	_	_
	M-381	*O*-Dealkylation	5.10	[C17H19N3O6+H]+	362.1347	251.1027	300.0982	227.0824	189.0657	172.0392
	M-367	*O*-Dealkylation+Ox	5.15	[C17H17N3O7+H]+	376.1139	358.1035	330.1083	265.0823	300.0988	189.0662
	M-345	*O*-Dealkylation	5.56	[C17H17F2N3O4S+H]+	398.0981	154.0224	139.0015	_	_	_
	M-301	*O*-Dealkylation	5.65	[C19H21F2N3O5S+H]+	442.1243	139.0013	_	_	_	_
	M+32	*O*-Dealkylation+Ox (Morph)	6.25	[C34H36F2N6O11S+H]+	371.0504	343.0565	_	_	_	_
	M-285	*O*-Dealkylation+Ox	6.34	[C19H21F2N3O6S+H]+	458.1192	241.0217	199.0021	139.0013	253.0242	143.0310
	M-241	*N*-Dealkylation+Ox	6.39	[C21H25F2N3O7S+H]+	502.1454	199.0030	139.0019	130.0327	299.0664	267.0413
	M-386	Amide hydrolysis+Ox	6.66	[C15H14F2N2O4S+H]+	357.0715	298.0603	154.0228	139.0016	143.0308	169.0334
**32**			8.83							
	M-368	*O*-Dealkylation	4.86	[C15H15N3O5+H]+	318.1088	207.0768	300.0985	172.0395	134.0607	116.0499
	M-324	*O*-Dealkylation	5.07	[C17H19N3O6+H]+	362.1344	251.1027	300.0980	227.0818	189.0659	172.0394
	M-310	*O*-Dealkylation	5.16	[C17H17N3O7+H]+	376.1139	358.1036	330.1084	265.0826	300.0982	189.0664
	M-345	*O*-Dealkylation	6.44	[C15H14F2N2O3S+H]+	341.0766	297.0501	154.0225	143.0304	139.0012	_
	M-301	*O*-Dealkylation	6.54	[C17H18F2N2O4S+H]+	385.1027	297.0507	139.0015	_	_	_
	M-329	*O*-Dealkylation	6.64	[C15H14F2N2O4S+H]+	357.0715	298.0579	187.0030	154.0226	143.0305	139.0018
	M+32	*O*-Dealkylation	6.73	[C32H33F2N5O10S+H]+	718.1987	227.0814	189.0656	172.0394	144.0446	116.0498
	M-285	*O*-Dealkylation	7.34	[C17H18F2N2O5S+H]+	401.0980	299.0662	240.0294	154.0228	143.0308	139.0014
	M-241	*N*-Dealkylation	7.5	[C19H22F2N2O6S+H]+	445.1239	103.0398	139.0018	299.0674	151.0358	130.0321
	M+18	Amide Hydrolysis	7.89	[C32H35F2N5O9S+H]+	704.2196	299.0063	227.0821	189.0666	172.0399	116.0499
	M-2	Dehydrogenatio n	8.03	[C32H31F2N5O8S+H]+	684.1934	297.0509	227.0817	189.0661	172.0395	116.0499

	M-2	Dehydrogenation	9.18	[C32H31F2N5O8S+H]+	648.1934	297.0517	227.082	189.0664	172.0399	116.0502
**33**			7.96							
	M-338	Amide Hydrolysis	4.94	[C28H41N5O4S+H2]++	272.6512	_	_	_	_	_
	M-525	Amide Hydrolysis	6.63	[C15H14F2N2O4S+H]+	357.0715	_	_	_	_	_
	M+32	*O*-Dealkylation+Ox (Morph)	6.58	[C43H53F2N7O9S2+H2]++	457.673	_	_	_	_	_
	M+16	Aliphatic Hydroxylation	7.43	[C43H53F2N7O8S2+H]+	898.3438	_	_	_	_	_
**34**			7.91							
	M-338	Amide Hydrolysis	4.98	[C29H43N5O4S+H2]++	279.6591	264.138	166.5751	332.1428	202.0687	141.0917
	M-564	Amide Hydrolysis	5.91	[C17H21N3O2S+H]+	332.1428	202.0691	91.0546	_	_	_
	M-337	*N*-Dealkylation	5.91	[C28H38N4O6S+H]+	559.2585	332.1428	202.0686	175.0578	131.0861	_
	M+32	*O*-Dealkylation+Ox (Morph)	6.4	[C44H55F2N7O9S2+H2]++	464.6808	727.2936	597.2191	332.1429	202.0685	141.0699
	M-200	Amide Hydrolysis	6.55	[C32H43F2N5O8S+H]+	696.2873	452.1454	311.0659	339.0616	199.0025	_
	M-539	Amide Hydrolysis	6.63	[C15H14F2N2O4S+H]+	357.0715	298.0588	139.0202	125.0202	_	_
	M+32	*O*-Dealkylation+Ox (Morph)	6.76	[C44H55F2N7O9S2+H2]++	464.6808	727.2929	597.2195	332.1428	202.0686	141.0699
	M-526	*N*-Dealkylation	6.99	[C16H17F2N3O3S+H]+	370.1031	139.012	125.0198	_	_	_
	M+14	Aliphatic Carbonylation	7.20	[C44H55F2N7O8S2]++	455.6755	446.6711	332.1444	202.0685	141.0699	115.0547
	M+16	Aliphatic Hydroxylation	7.38	[C44H55F2N7O8S2+H]+	912.3594	537.2369	452.1482	339.0617	311.0683	218.0644
	M+16	Aliphatic Hydroxylation	7.52	[C44H55F2N7O8S2+H2]++	456.6834	563.2145	447.6786	332.1423	202.0686	115.0546
	M-14	*N*-Dealkylation	7.97	[C43H53F2N7O7S2+H2]++	441.6781	681.29	551.2139	332.1436	202.0687	
**35**			8.38							
	M-323	*N*-Dealkylation	6.27	[C30H42N4O6S+H]+	587.2898	202.0687	175.058	131.0859	_	_
	M-553	Amide Hydrolysis	6.63	[C15H14F2N2O4S+H]+	357.0715	298.0595	187.0038	139.0229	125.02	71.9913
	M+32	*O*-Dealkylation+Ox (Morph)	6.95	[C45H57F2N7O9S2+H2]++	471.6886	741.3095	611.2361	332.1427	202.0678	141.0702
**36**			8.02							
	M-566	Amide Hydrolysis	4.81	[C17H21N3O2S+H]+	332.1427	202.0687	91.0551	_	_	_
	M-338	Amide Hydrolysis	4.92	[C28H41N5O5S+H2]++	280.6487	332.1433	202.0688	141.0704	86.0972	_
	M-395	*O*-Dealkylation	5.74	[C25H34N4O5S+H]+	503.2323	332.143	202.0687	175.0578	141.0701	_
	M-470	*O*-Dealkylation	6.35	[C18H19F2N3O5S+H]+	428.1086	241.022	199.0017	139.0016	125.0201	71.9912
	M+32	*O*-Dealkylation+Ox (Morph)	6.66	[C43H53F2N7O10S2+H2]++	465.6704	729.2722	599.199	465.6715	202.0687	141.0701
	M+16	Aliphatic Hydroxylation	7.45	[C43H53F2N7O9S2+H2]++	457.6730	695.2706	565.1933	448.6696	202.0691	332.1427
**37**										
	M-338	Amide Hydrolysis	4.94	[C29H43N5O6S+H2]++	295.654	332.1426	231.1704	202.0684	141.0698	91.0549
	M-530	*O*-Dealkylation+Ox	5.51	[C17H17F2N3O4S+H]+	398.0981	125.0196	_	_		_
	M-486	*O*-Dealkylation+Ox	5.61	[C19H21F2N3O5S+H]+	442.1243	267.0403	184.9865	125.0203	71.991	_
	M-425	*O*-Dealkylation	5.74	[C25H34N4O5S+H]+	503.2323	285.1446	332.1423	202.0683	175.0575	141.0698
	M-381	*O*-Dealkylation	5.77	[C27H38N4O6S+H]+	547.2585	332.1429	202.0686	141.0701	91.055	_
	M-367	*O*-Dealkylation+Ox	5.82	[C27H36N4O7S+H]+	561.2377	332.1427	202.0685	141.0698	_	_
	M-528	*O*-Dealkylation	6.16	[C17H19F2N3O4S+H]+	400.1137	143.0302	139.0014	75.0236	71.9909	
	M-484	*O*-Dealkylation	6.24	[C19H23F2N3O5S+H]+	444.1399	299.0674	253.0260	241.0220	199.0023	139.0015
	M-427	*O*-Dealkylation+Ox	6.25	[C25H32N4O5S+H]+	501.2166	330.1268	172.0965	142.0781	202.0683	141.0702
	M-470	*O*-Dealkylation+Ox	6.27	[C19H21F2N3O6S+H]+	458.1192	241.0215	199.0023	139.0011	130.0321	125.0198
	M+32	*O*-Dealkylation+Ox (Morph)	6.52	[C44H55F2N7O11S2+H2]++	480.6757	187.0448	759.2848	629.2091	332.1419	202.0684
	M-571	Amide Hydrolysis	6.6	[C15H14F2N2O4S+H]+	357.0715	75.0047	298.058	125.0198	184.9866	139.0228

	M+176	Glucuronidation (OH)	7.19	[C50H64F2N715S2+H2]++	552.6969	903.3298	597.2212	508.1758	202.0691	141.0702
	M-2	Dehydrogenation	7.35	[C44H54F2N7O9S2+H2]++	463.673	725.2774	595.2038	463.6737	332.1432	202.0687
**38**			9.79							
	M+32	*O*-Dealkylation+Ox (Morph)	8.01	[C44H56F2N6O8S2+H2]++	450.1857	698.3037	568.2289	332.1428	202.0685	_
	M-2	Dehydrogenation	9	[C44H55F2N6O6S2+H]++	433.183	297.0513	534.2233	506.2292	332.1429	202.0687
**39**			8.25							
	M-607	*O*-Dealkylation	5.06	[C13H12F2N2O2S+H]+	299.0661	86.0059	_	_	_	_
	M-266	*O*-Dealkylation+Ox	5.74	[C31H41N7O6S+H]+	640.2912	439.2300	332.1427	202.0685	168.1383	115.0542
	M+32	*O*-Dealkylation+Ox (Morph)	6.64	[C44H53F2N9O8S+2H]++	469.6787	607.2145	332.1427	219.0950	202.0685	115.0542
	M+32	*N*-Dealkylation+Ox (Morph)	7.08	[C44H53F2N9O8S+2H]++	469.6788	607.2145	494.1304	332.1427	219.0950	202.0685
	M-426	Amide Hydrolysis+Ox	7.22	[C21H23F2N5O4S+H]+	480.1512	_	_	_	_	_
	M-2	Dehydrogenation	7.64	[C44H52F2N9O6S2+H]++	452.6759	573.2090	545.2141	332.1427	297.0504	202.0685
**40**			8.04							
	M-323	*N*-Dealkylation	5.98	[C34H46FN5O7S+H]+	688.3175	115.0545	489.2162	228.0474	202.0683	172.113
	M-655	*N*-Dealkylation	6.51	[C15H15F2N3O3S+H]+	356.0875	240.0283	166.996	143.0299	125.0198	298.0568
	M-654	Amide Hydrolysis	6.59	[C15H14F2N2O4S+H]+	357.0715	298.0576	240.0290	166.9963	139.0230	125.0200
	M+32	*O*-Dealkylation	6.68	[C49H61F3N8O10S2+H2]++	522.2025	422.6524	202.0686	712.1133	141.0700	115.0546
	M-2	Dehydrogenation	7.46	[C49H60F3N8O8S2+H2]++	505.1997	405.6499	267.0399	202.0687	680.2171	712.1134
	M+16	Aliphatic Hydroxylation	7.53	[C49H61F3N8O9S2+H2]++	514.205	356.0875	158.0974	414.6546	202.0684	_
**41**			4.80							
	M+18	Amide Hydrolysis	2.69	[C13H13N3O5+H]+	292.0928	201.0656	_	_	_	_
	M+18	Amide Hydrolysis	2.82	[C13H13N3O5+H]+	292.0928	201.0657	164.0337	129.0660	84.0452	_
	M+18	Amide Hydrolysis	3.65	[C13H13N3O5+H]+	292.0928	247.0714	229.0604	201.0656	163.0540	_
	M+18	Amide Hydrolysis	3.73	[C13H13N3O5+H]+	292.0928	274.0811	246.0867	229.0603	201.0655	163.0497
**42**			4.72							
	M-227	*N*-Dealkylation	3.24	[C12H11NOS+H]+	218.0634	115.0545	_	_	_	_
	M+16	Aromatic Hydroxylation	4.35	[C23H32N4O4S+H]+	461.2217	348.1374	218.0635	115.0548	_	_
	M+16	Aliphatic Hydroxylation	5.21	[C23H32N4O4S+H]+	461.2217	332.1426	243.1348	202.0686	141.0698	115.0547
	M+14	Aliphatic Carbonylation	5.97	[C23H30N4O4S+H]+	459.2061	245.0735	217.0800	202.0688	147.0269	115.0548
	M-227	*N*-Dealkylation	6.71	[C12H11NOS+H]+	218.0634	147.0265	115.0545	_	_	_
**43**			6.95							
	M+16	Aliphatic Hydroxylation	5.68	[C19H17ClN4O3S+H]+	417.0783	399.0674	_	_	_	_
**44**			7.50							
	M+16	Anilinic Hydroxylation	5.79	[C19H12ClN3O3+H]+	366.064	330.0882	_	_	_	
	M+176	Glucoronidation	5.94	[C25H20ClN3O8+H]+	526.1012	350.0694	314.0927	508.0910	304.0637	223.0503
	M+176	Glucoronidation	5.98	[C25H21ClN3O8]+	526.1012	304.0633	350.0698	314.0921	223.0500	195.0552
**45**			3.85							
	M+16	Aliphatic Hydroxylation	4.56	[C20H19FN4O3+H]+	385.1516	281.0727	208.0561	130.0289	233.0711	_
**46**			8.50							
	M+176	Glucoronidation (OH)	5.07	[C19H20F2N2O8S+H]+	475.0981	299.0660	255.0400	166.9962	139.0013	130.0323
	M+16	Aliphatic Hydroxylation	6.32	[C13H12F2N2O3S+H]+	315.0609	154.9967	141.0151	130.0328	114.0282	101.0205
	M+32	*O*-Dealkylation	6.42	[C13H12F2N2O4S+H]+	331.0559	285.0501	312.0446	166.9958	154.0227	125.0203
	M+32	*O*-Dealkylation	6.94	[C13H12F2N2O4S+H]+	331.0559	166.9959	125.0196	115.0356	_	_

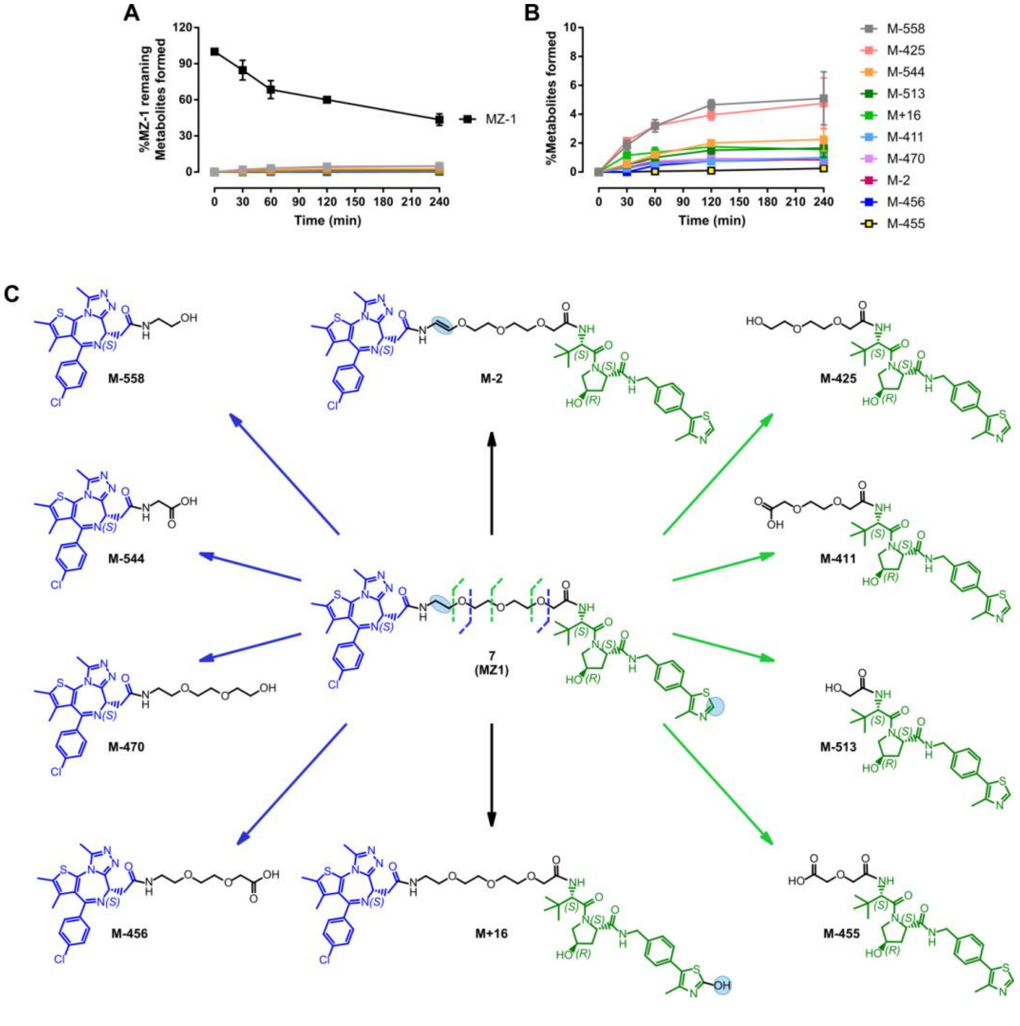

Figure S1. Metabolic profile for compound **7** (**MZ1**). A) Observed behavior for substrate degradation is shown in black; B) formation of metabolites, whose names are expressed referring to the mass variation compared to the one of substrate. Data were acquired in triplicate and standard deviation is provided; C) chemical structures of identified metabolites.

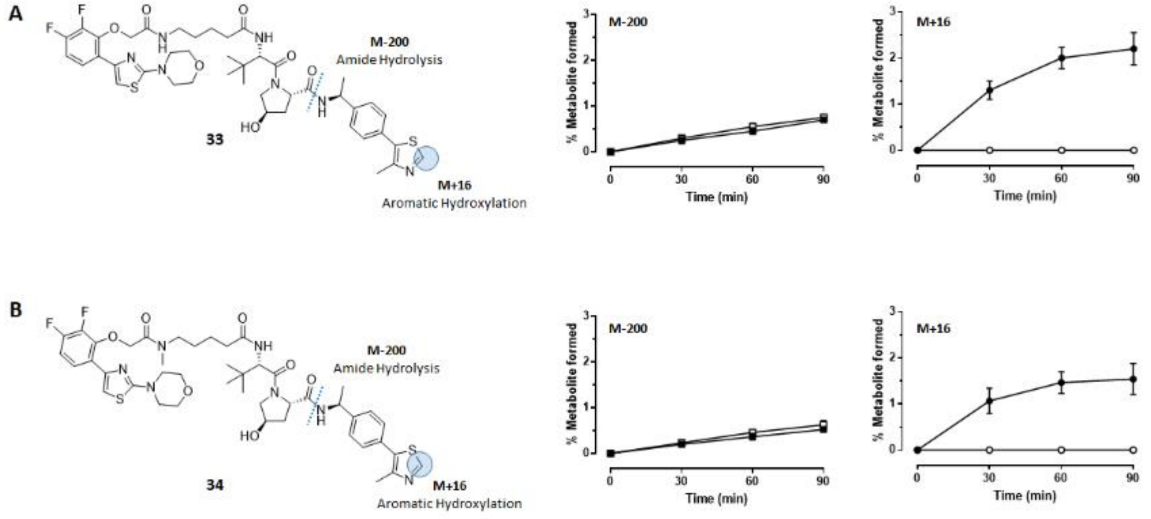

Figure S2. Kinetics of the metabolism of compounds **33** and **34** in human liver cytosol. Concerning the chemical structures, bond cleavages are illustrated as dotted lines, while blue circles indicate the atom subjected to oxidation. In the kinetic plots, the behavior of the hydrolyzed metabolite (M-200) is shown as squares, while the hydroxylated metabolite (M+16) is shown as circles. Filled or empty points refers to measures conducted in the absence or in the presence of hydralazine as *h*AOX inhibitor.

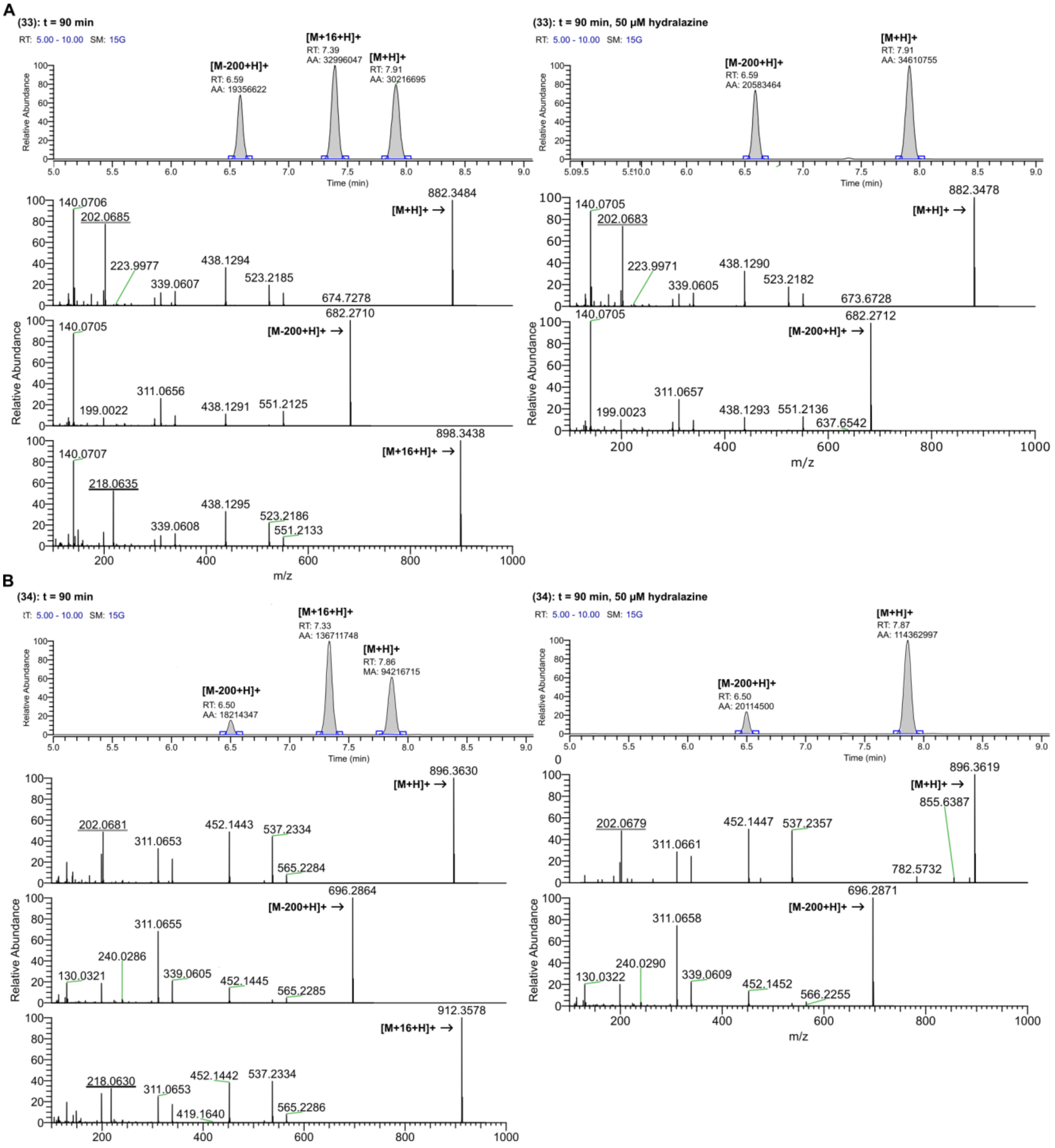

Figure S3. Chromatogram of **33** (A) and **34** (B) after 90 min incubation in human liver cytosol in the absence (left) and in the presence of hydralazine (50 µM) (right); the MS/MS spectra of the [M+H]+, [M-200+H]+ and [M+16+H]+ ions are reported with a mass tolerance of 5 ppm for the base peak.
